# Twenty-six new species of *Hoploscopa* (Lepidoptera, Crambidae) from South-East Asia revealed by morphology and DNA barcoding

**DOI:** 10.3897/zookeys.907.36563

**Published:** 2020-01-29

**Authors:** Théo Léger, Christian Kehlmaier, Charles S. Vairappan, Matthias Nuss

**Affiliations:** 1 Museum für Naturkunde – Leibniz-Institut für Evolutions- und Biodiversitätsforschung, Invalidenstr., Berlin, Germany Museum für Naturkunde Berlin Germany; 2 Senckenberg Museum für Tierkunde Dresden, Königsbrücker Landstr., Dresden, Germany Senckenberg Museum für Tierkunde Dresden Dresden Germany; 3 Institute for Tropical Biology and Conservation, Universiti Malaysia Sabah, 88400 Kota Kinabalu, Sabah, Malaysia Universiti Malaysia Sabah Sabah Malaysia

**Keywords:** Indo-malayan, integrative taxonomy, historic DNA, Melanesia, Pyraloidea, taxonomy

## Abstract

*Hoploscopa* Meyrick (Lepidoptera: Crambidae) is a fern-feeding genus found in montane areas of South-East Asia and Melanesia, eastwards up to the Samoan Islands. It includes sixteen described species, with at least 70 further undescribed species known from scientific collections. An iterative approach including morphological and molecular characters was used in order to explore the diversity of *Hoploscopa*. The hitherto described species are revised, and descriptions authored by T. Léger and M. Nuss are provided for an additional 26 new species: *H. agtuuganonensis***sp. nov.**, *H. albipuncta***sp. nov.**, *H. albomaculata***sp. nov.**, *H. anacantha***sp. nov.**, *H. boleta***sp. nov.**, *H. cynodonta***sp. nov.**, *H. danaoensis***sp. nov.**, *H. gombongi***sp. nov.**, *H. gracilis***sp. nov.**, *H. ignitamaculae***sp. nov.**, *H. isarogensis***sp. nov.**, *H. jubata***sp. nov.**, *H. kelama***sp. nov.**, *H. kinabaluensis***sp. nov.**, *H. mallyi***sp. nov.**, *H. marijoweissae***sp. nov.**, *H. matheae***sp. nov.**, *H. niveofascia***sp. nov.**, *H. pangrangoensis***sp. nov.**, *H. parvimacula***sp. nov.**, *H. pseudometacrossa***sp. nov.**, *H. sepanggi***sp. nov.**, *H. sumatrensis***sp. nov.**, *H. titika***sp. nov.**, *H. tonsepi***sp. nov.**, *H. ypsilon***sp. nov.** Using a protocol specific for the amplification of DNA from old museum specimens, we recovered 101 COI barcodes for all but one of the newly described species, with 76 being barcode compliant (>487 bp). Species delimitation analyses suggest cryptic diversity, with six cases reflecting allopatric divergence, and two further cases found in sympatry.

## Introduction

South-East Asia is home to a rich biodiversity encompassing three of the 25 world’s biodiversity hotspots (Myers et al. 2000). Pyraloidea is one of the largest superfamilies of Lepidoptera in the Oriental region, with 3,771 known species (Heppner 1998). Much of its diversity for this region remains to be described in this group (Nuss 1998, Sutton et al. 2015). *Hoploscopa* Meyrick best illustrates this knowledge gap, with 16 species currently described and more than 70 species awaiting description (Robinson et al. 1994). *Hoploscopa* moths display elongated brown forewings often with median and postmedian diagonal pale yellow to red markings. The genus is distributed across the Oriental and Australasian regions, ranging from the North of Thailand eastwards to the Samoa Islands, but is virtually absent from the tropical rainforests of Northern Queensland, Australia. A recent study reported the larvae to feed on ferns (Mally et al. 2017).

Nuss (1998) synonymised *Syncrotaula* Meyrick, a replacement name for *Eudorina* Snellen, with *Hoploscopa*, and transferred twelve species to it. The author also provided a checklist of the genus and described one new species. Robinson et al. (1994) erected the tribe Hoploscopini within Scopariinae for *Hoploscopa* (= *Syncrotaula*) and *Perimeceta* Turner. Nuss (1998) transferred the Hoploscopini to the Heliothelinae based on the shared inwardly directed spine of the corpus bursae in the female genitalia. *Perimeceta*, the only other genus included in the Hoploscopini, is distributed from Java to South Australia and its larvae also feed on ferns (Miller et al. 2015, Mally et al. 2017). The systematic placement of Hoploscopini is still a matter of debate, with some authors grouping them together with Heliothelini in the Heliothelinae (Nuss 1998), while others consider the Heliothelinae an ingroup of Scopariinae (Leraut 1980, Minet 1982, Munroe and Solis 1998). The first molecular-based phylogeny including representatives of *Hoploscopa* suggest it to represent a separate lineage outside of Scopariinae + Crambinae (Léger et al. 2019).

This paper aims at establishing an iterative approach following Yeates et al. (2011) for species discovery and description in *Hoploscopa*, serving as a first step of a comprehensive revision of the genus. With an iterative approach using morphology and COI barcode, we provide redescriptions for fifteen of the 16 described species and describe 26 new species. Incongruence between morphology and molecular datasets, as well as factors that possibly influenced the evolution of the group are discussed.

## Materials and methods

### Material acquisition

Material collected on Borneo, Java, Fiji, the Malay Peninsula, North-Sulawesi, New Guinea, the Philippines, Samoa, Sumatra and Vanuatu was obtained from the Museum für Tierkunde Dresden (**MTD**), the British Natural History Museum, London (**NHMUK**), the United States National Museum, Washington (**USNM**), the Museum für Naturkunde Berlin (**MFNB**) and the Zoologische Staatsammlung München, Munich (**ZSM**). Examination of the type specimens of the previously described species, all deposited at the NHMUK, was done by TL during a visit at that institution. Specimens described from Sabah, Malaysia are stored at the Institute for Tropical Biology and Conservation, Borneensis, Kota Kinabalu, Malaysia (**BORN**).

### Iterative approach

In order to test species hypotheses with two independent sets of characters, we followed the best practice outlined by Schlick-Steiner et al. (2010), here referred to as “iterative taxonomy” (Yeates et al. 2011). This workflow started by sorting undescribed material to morphospecies based primarily on wing pattern, followed by examination of characters in male genitalia that show most variation. Subsequently, for each morphospecies, specimens with slightly differing wing pattern and/or of each collecting locality were selected for amplification of the mitochondrial COI barcode. Finally, morphospecies with at least one obtained COI barcode sequence were considered for species description (with the exception of *H. marijoweissae* sp. nov.). If strong divergence was observed in the COI barcode within a morphospecies, a second more careful examination of the specimens was conducted in the search of diagnostic characters, and COI barcode was sequenced for all specimens of the series. Specimens in poor conditions (i.e., wing pattern faded away, abdomen missing) or new species without male specimens available were not further considered for species description.

### Molecular work

One hundred and fifty-seven dried museum specimens were considered for DNA extraction. Assuming a higher degree of degradation due to the age of the dried specimens, DNA extraction and preparation of PCR samples for specimens collected before 1990 (referred to as “old sample”) were performed in the clean-room facility of the SGN-SNSD-Mol-lab at MTD. The abdomen (one or two legs if the abdomen could not be used) was carefully removed with sterilised pincers for DNA extraction, following a non-destructive method (Knölke et al. 2004) with the NucleoSpin Tissue kit (Macherey-Nagel, Düren, Germany) according to the manufacturer’s protocol. DNA concentration of old samples was measured with a NanoDropOne spectrophotometer. We followed a workflow previously used by other authors (Hebert et al. 2013, Mitchell 2015, Hundsdoerfer et al. 2017) by amplifying up to five short overlapping fragments covering the COI barcode part (see Suppl. material 1: Fig. S1). Primers, amplicon length and amplicon overlap are listed in Suppl. material 2: Table S1. In the first step (recently collected samples only), amplification of the whole COI barcode sequence (658 bp) was attempted with the primer pair HybLCO/HybNancy, each flanked with universal primer tails (“Hyb-”) facilitating sequencing (Wahlberg and Wheat 2008). If amplification was not successful, we proceeded in the second step with the amplification of fragments 1a (LepF1/K699) and 1b (f220/LepR1 or HybNancy). The latter reverse primer showed a slightly better performance with respect to the amplification of fragment 1b and was preferentially used. If amplification of fragments 1a or 1b in step two failed, we proceeded in step three with amplification of fragments A, B, C (covering fragment 1a) and C, D, E (covering fragment 1b). PCR-mix included 2 µl of BIORON complete buffer 10× incl. Mg^2+^ (25 mM), 2 µl of each primer at 10pmol/µl, 0.4 µl of dNTP (each 10 mM), 0.2 µl of Taq polymerase BIORON (Bioron DFS Taq, Ludwigshafen, Germany) ,1 µl (recent samples) or 1 to 8 µl (old samples) of DNA (depending on the quantity of DNA measured) and filled up with water to final volume of 20 µl. Alternatively, the AccuStart II GelTrack PCR SuperMix (2×) (Quanta BioSciences, Beverly, USA) ready-to-use mix was used in the following PCR-mix: 6.5 µl AccuStart ready-to-use-mix, 1 µl of each primer, 1–8 µl DNA and filled up with water to final volume of 25 µl. Initial denaturation at 95 °C during 5 min was followed by 42 cycles of 30 sec denaturation at 95 °C, 40 sec hybridisation at 49 °C, 50 sec elongation at 72 °C, with a final elongation at 72 °C for 10 min. Amplification success was checked by electrophoresis on 1 or 2% agarose gels, subsequently stained with GelRed and visualised under UV light. Sequencing was performed by Macrogen (Netherlands) using the original PCR primers or the T7 and T3 sequencing primers (Wahlberg and Wheat 2008). Full COI-barcoding PCR products were sequenced in the forward direction only, while the shorter PCR products were sequenced in both directions.

### Data analyses

Sequences were checked by eye and concatenated using PHYDE 0.9971 (Müller et al. 2005). In order to assess the amplification of the correct fragment, sequences were blasted with the blastn program against the nucleotide collection (nt), as provided by the National Center for Biotechnology Information (NCBI). In order to identify potential nuclear pseudogenes, sequences were searched for indels, internal stop codons and double peaks in electropherograms, as suggested in Song et al. (2008). Newly generated sequences (including ENA and BOLD accession numbers), as well as sequences retrieved from Barcode of Life Data System (BOLD, http://v4.boldsystems.org/) are reported in Suppl. material 3: Table S2. For the phylogenetic analyses, PartitionFinder2 (Lanfear et al. 2017) was used to determine the best partitioning scheme using the AICc model and the greedy search algorithm (Lanfear et al. 2012). Partition for each of the three codon positions was recovered as the best model and subsequently used for further analyses. Maximum Likelihood (ML) analysis was performed with RAxML (Stamatakis 2006) as implemented on the CIPRES portal (Miller et al. 2010), using the GTR+GAMMA substitution model and the best PartitionFinder scheme. Node support was estimated with 1000 thorough bootstrap replicates using the same algorithm.

### Species delimitation

Two different methods were used to investigate species delimitation in our molecular dataset: Automatic Barcode Gap Discovery (ABGD) (Puillandre et al. 2012) and General Mixed Yule-coalescent method (GMYC) (Pons et al. 2006, Fujisawa and Barraclough 2013). These popular methods have been repeatedly used in DNA barcoding studies on Lepidoptera (Kekkonen and Hebert 2014, Dincă et al. 2015, Dumas et al. 2015, Nakahara et al. 2019). We noticed a detrimental effect of non-compliant barcode sequences (< 487 bp) on the estimation of species in densely sampled clades (n > 2), where these sequences were flagged as new species. Consequently, these sequences were removed to generate a COI_487 bp dataset. Distance-based analysis ABGD was calculated using the K80 Kimura distance model and X fixed to 0.5 on the dedicated platform (https://bioinfo.mnhn.fr/abi/public/abgd/abgdweb.html). Genetic distances are referred to as K2P-dist throughout the text.

The ultrametric tree required for the GMYC analysis was generated using BEAST 1.10.4 (Suchard et al. 2018). The COI dataset was partitioned after codon position accordingly to the best PartitionFinder2 scheme. An uncorrelated relaxed clock with lognormal distribution was used and the Speciation: Yule Process model was set as tree prior model and other parameters were left unchanged. MCMC chain was set to 40 million generations, sampled every 1000^th^ generation. Convergence was checked on Tracer (Rambaut et al. 2018) and the first 4 million generations were discarded as burnin. Species delimitation analysis was performed using the GMYC method as implemented in the R-package SPLITS (Ezard et al. 2009, Fujisawa and Barraclough 2013). The single-threshold option was used as suggested in Fujisawa and Barraclough (2013).

### Systematic treatment

Genitalia were mounted following the method of Robinson (1976). Photographs of the habitus of imagines were taken with an Olympus E-M1 with the Olympus M. Zuiko Digital ED 60 mm f/2.8 Macro Lens. Photographs of genitalia and measurements were performed on a Nikon Eclipse 90i at the MTD. Images were subsequently enhanced on Adobe Photoshop CS6 and illustrations plates were created on Adobe Illustrator CS6. Collecting data of holotypes was copied exactly as found on the labels, with vertical bars to mark line breaks. Abbreviations or translations are given in square brackets where judged meaningful. Paratype data are reported by country in alphabetical order, with information reported without indication of line change. Collecting localities are reported as written on labels. Dates and collectors’ information were standardised and the latter placed in parentheses. The specimen depositories are reported with the use of the corresponding acronyms. GPS coordinates for localities were retrieved from the online platform geographic.org (https://geographic.org/geographic_names/) using the database of the National Geospatial-Intelligence Agency. Localities treated in this paper are reported in Table A1 in Appendix 1. Specimens whose conspecificity was uncertain were listed under “Other specimens examined”. Nomenclature follows that of Landry (1995), except for the use of the term phallus (Kristensen 2003). The colours “bronze”, “ochre”, and “tawny” refer to the Wikipedia list of colours by shade for brown (https://en.wikipedia.org/wiki/Category:Shades_of_brown). Character description was facilitated by the use of Mesquite (Maddison and Maddison 2017) where each character was given a state, thereby allowing a better visualisation of character state variation as well as warranting consistency among species descriptions.

## Results

### Molecular dataset

We obtained 101 COI barcodes ranging in length from 261 to 658 bp (mean length = 545 bp), among them 76 barcode “compliant sequences” (> 487 bp) according to Hebert et al. (2013). Ten non-compliant barcode sequences were kept in the final dataset for species with no or one barcode sequence. Fifteen further non-compliant barcode sequences were discarded from the final dataset (see Suppl. material 2: Tab. S1). We recovered the COI barcode for all but one (*H. marijoweissae* sp. nov.) species described in this paper and for seven of the sixteen previously described species. Twenty-five *Hoploscopa* sequences, as well as 13 sequences from the hypothesised sister-group *Perimeceta* were retrieved from BOLD and added to our dataset, totalling 126 COI barcode sequences including nine of the described species. Eleven species were represented by only one barcode sequence. Recovered fragments and total sequence length are summarised in Suppl. material 3: Table S2. The oldest sample from which we could successfully amplify COI sequence data were collected in 1955 (63 years old). The Maximum Likelihood analysis of the COI dataset is illustrated in Fig. 123, with bootstrap support (abbr. BS) over 50 displayed on nodes. Genetic distances (K2P-dist) between all COI barcodes are reported in Suppl. material 4: Table S3.

### Species delimitation

Analysis of the morphology allowed delineation of 39 species. Both ABGD and GMYC analyses highlighted 48 molecular operational taxonomic units (MOTUs) in *Hoploscopa* species, splitting seven of the morphospecies into two MOTUs, and one into three MOTUs (*H. danaoensis* sp. nov.). Six of these splits reflect allopatric divergence. In each of the two species *H. matheae* sp. nov. and *H. parvimacula* sp. nov., there is one female from the Malay Peninsula recovered as a separate MOTU from other specimens from Borneo. In *H. luteomacula*, the two specimens from Borneo are recovered as a distinct MOTU to that of Sumatra. In *H. albipuncta* sp. nov., a female from Luzon (Philippines) was recovered in a MOTU distinct from that including the specimens from Borneo. Genetic differentiation is also observed among Philippines islands: specimens of *H. danaoensis* sp. nov. from Danao, Negros, and Mindanao represent three different MOTUs, while specimens of *H. isarogensis* sp. nov. from Luzon and Leyte are also recovered into two distinct MOTUs.

Sympatric divergence is observed in *H. kinabaluensis* sp. nov. and *H. sumatrensis* sp. nov. *Hoploscopa kinabaluensis* sp. nov. is split into two MOTUs representing sister clades and diverging from each other by 1.7–2.2%. In *H. sumatrensis* sp. nov., two separate MOTUs are recovered by the ML analysis in a clade with DNA samples BC_MTD_LEP01421 (Hoploscopa sp. near sumatrensis) and BC_ZMBN_Lep00081 (larva of *Hoploscopa* sp.). The two *H. sumatrensis*MOTUs show a divergence of 4.1–6%. Second MOTU, represented by samples MTD8258 and BC_MTD_LEP01422, include only females and need examination of male specimens.

### Systematics

#### Hoploscopa

Taxon classificationAnimaliaLepidopteraCrambidae

Meyrick, 1886

32794D66-3A52-5E4F-937B-1F932F002558

Haploscopa Hampson, 1897: 223.
Syncrotaula Meyrick, 1933: 378. Type species Eudorina aurantiacalis Snellen, 1895, by subsequent designation (for Eudorina Snellen, 1895) by Joannis, 1930.

##### Type species.

*Hoploscopa astrapias* Meyrick, 1886, by monotypy.

##### Diagnosis.

*Hoploscopa* displays brown to dark brown forewings, often bearing pale yellow-, yellow-, or red-coloured median and postmedian diagonal stripes. In male genitalia, the uncus is well developed, the gnathos forms a ribbon-like structure, often with a posterior projection, and the vinculum bears laterally a pair of coremata. In female genitalia, the antrum is short, often sclerotised, and the corpus bursae bears a thorn, often with a basal sclerotisation. *Hoploscopa* is morphologically very similar to *Perimeceta*. The forewings of *Perimeceta* are slightly larger, display a yellow to brown ground colour, with one basal elliptic and one postmedian lunule-shaped white spot. In male genitalia, *Perimeceta* shows a spade-shaped uncus, the gnathos arms connect shortly after arising and are expanded posteriorly into an elongated tip, the valva shows a conspicuously rounded ventral margin, and is apically narrowed into a tip, while the ventral margin is more or less straight in *Hoploscopa*. *Perimeceta* exhibits female genitalia similar to those of *Hoploscopa* with a slender ductus bursae and a rounded corpus bursa bearing a thorn. However, the latter displays a membranous antrum in female genitalia, while it is sclerotised in most species of *Hoploscopa*.

##### Description.

***Head.*** Antennae dorsally with pale yellow to brown scales. Ocelli absent. Frons slightly produced, rounded. Proboscis basally white to brown scaled. Maxillary palpi brown to dark brown, first segment pale yellow, inner side brown to pale yellow. Labial palpi porrect, 2–2.9 × diameter of compound eye, brown, white to pale yellow at base, inner side brown to pale yellow.

***Thorax*** (Figs 1–45). Collar white to pale yellow. Frenulum simple in ♂, triple in ♀. Forewing length 7–13 mm, 2.4–2.9 × maximal width, females slightly larger than males. Wing venation (Fig. 2): R1 not connected to Sc; R2, R3 and R4 stalked together; R5 free, arising from upper corner of cell; M1, M2 not stalked; M3 arising from lower corner of cell; CuA1 arising below lower corner; CuA2 at distal 1/3 of cell; 1A+2A arising from cell base; Hindwing Sc+R1 connected to Rs at distal 1/3; M1 connected to Sc+R1 by short vein; cell closed; M2, M3 and CuA1 arising at lower angle of cell; CuA2 arising at middle of cell; 1A, 2A, 3A free. Forewing ground colour brown to dark brown, markings white, yellow or red colour. The pattern elements can be described as follows (Fig. 3):

**Figures 1–3.**
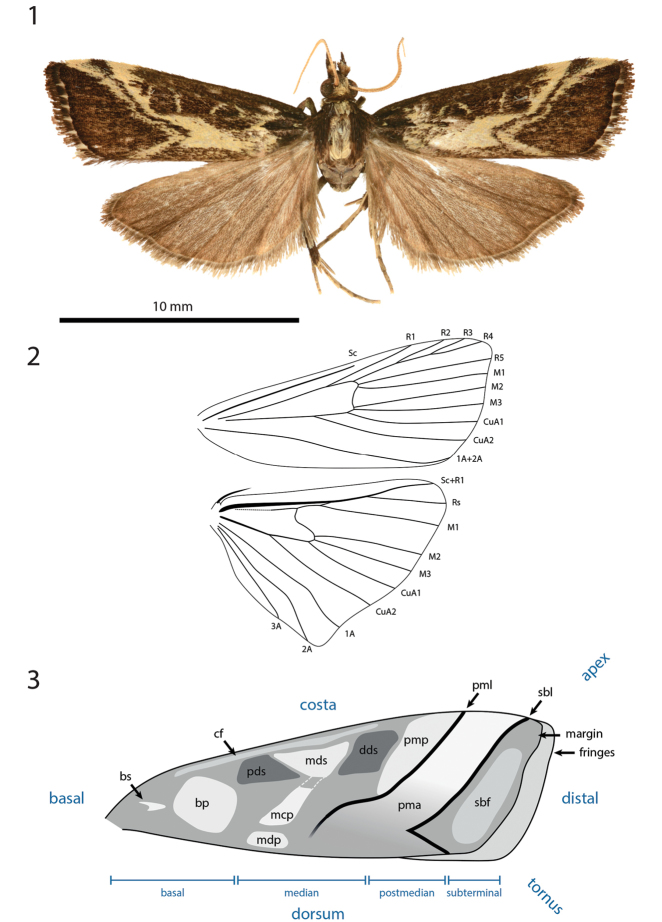
Habitus, venation and forewing pattern in *Hoploscopa*. **1** Habitus of *Hoploscopa matheae* sp. nov., holotype, ♀, Malaysia, Sabah, Kundasang, Kinabalu Mt. Lodge veranda, 6°0'42.15"N, 116°32'3.63"E, 1570 m (T. Léger & R. Mally) (genitalia on slide TL315 ♀) **2** wing venation of *Hoploscopa* species: *H. jubata* sp. nov., paratype, ♂, Papua New Guinea, Morobe Prov., nr Bulolo, Mt Susu Nat. Res., *Araucaria* For., 975 m, 27–28 Aug 1983 (S. Miller) (wing preparation TL706) **3** nomenclature used for description of forewing pattern of *Hoploscopa* spp. Abbreviations: bp (basal patch), bs (basal streak), cf (costal field), dds (distal discoidal stigma), mcp (median cubital patch), mds (median discoidal stigma), mdp (median dorsal patch), pds (proximal discoidal stigma), pma (postmedian area), pml (postmedian line), pmp (postmedian patch), sbf (subterminal field), sbl (subterminal line).

**cf** costal field stretched along costa up to postmedian area;

**bs** basal streak small, weakly or not marked;

**bp** basal patch often quadrangular, ill-defined;

**pds** and **dds** proximal and distal discoidal stigmata along costal field;

**mds** the median discoidal stigma trapezoid, with costal edge longer than dorsal one;

**mcp** and **mdp** median cubital patch and median dorsal patch in line with median discoidal stigma, often forming together a Y-shape pattern;

**pmp** postmedian patch roughly triangular, narrowing at costa;

**pml** postmedian line running from costa to middle of dorsum, often barely marked, mostly visible close to costa;

**pma** postmedian area often suffused to a various extend with white, yellow or red;

**sbl** subterminal line running from costa close to apex towards dorsum, often incurved inwards at CuA2;

**sbf** subterminal field more or less suffused with brown, red or yellow.

The margin is brown, in some species with spots, fringe unicoloured or chequered.

Hindwing upper side pale yellow to pale brown; underside pale yellow, with brown markings on costa and subterminal line toward costa; males of some species with androconial organ on the upper side at dorsum, consisting of upright scales along CuA2 and 1A veins and a protruded margin between CuA2 and 1A, bearing a patch of greyish scales. Forelegs brown to dark brown. Midlegs with femur brown, tibia and tarsi often pale yellow, speckled with brown. Hindlegs pale yellow on inner side, pale yellow to brown on outer side, tarsi bronze to pale brown.

***Tympanal organs*** (Figs 83–84).

**tb** Tympanic bridge deeply divided in the centre;

**td** Tympanic drum (Fig. 50) ovoid, antero-ventrally semi-closed, posteriorly not reaching transverse ridge;

**tdp** Tympanic depressions broad, opened ventrally;

**tm** Tympanum subtriangular;

**tr** Transverse ridge (“”) not sclerotised.

***Abdomen.*** Pale brown to brown. In males, sternum A8 more or less broadly indented, in some species with short, rounded lateral projections (Figs 85, 86).

***Male genitalia*** (Figs 46–82).

**c** cornutus on phallus elongated, flat, spatula-shaped, except otherwise mentioned;

**de** ductus ejaculatorius;

**g** Gnathos arms (Fig. 54) originating from the dorsal part of tegumen, forming ring, with or without dorso-median extension;

**jx** Juxta basally wide with anterior margin rounded to quadrangular, narrowing at basal 1/4;

**s** saccus triangular to quadrangular, pointing dorsally;

**ta** Tegumen arms anteriorly sclerotised, with edge marked, posteriorly membranous; dorsally fused into a bridge of various width, articulated or fused to uncus base;

**uc** Uncus well-developed, densely haired apically;

**v** Valva elongated, hairy, sclerotised, with strongly sclerotised dorsal edge;

**vc** Vinculum U-shaped in posterior view, dorso-laterally on each side with anterior projection bearing brush-shaped coremata.

***Female genitalia*** (Figs 87–122).

**aa** Anterior apophyses (Fig. 88) bluntly angled at posterior 1/3, bent ventrad;

**at** Antrum sclerotised or membranous;

**cb** Corpus bursae globular or ovoid, its membrane with a reticulate structure;

**cs** Broad sclerotisation of the corpus bursae;

**db** Ductus bursae membranous with longitudinal wrinkles, variable in length and shape;

**pa** Posterior apophyses slender, straight, directed posterad except otherwise mentioned;

**pp** Papillae anales thin in lateral view, dorsally and ventrally connected, setose;

**segm. VIII** Segment VIII faintly sclerotised, ventrally membranous; setae scattered across segment, more densely concentrated on posterior margin;

**t** thorn on corpus bursae large, sclerotised, inwardly projecting, its base slighly extending outwards into a small sclerotised protuberance.

##### Distribution.

*Hoploscopa* is found from the Malay Peninsula and Sumatra in the West to the Samoa Islands in the East, as well as from the North of Thailand to Vanuatu and Fiji in the South. It is absent from Queensland (Australia) and New Caledonia. It is predominantly found in tropical mountain forests, with only few species encountered in the lowlands.

##### Biology.

Host plant data is available for five *Hoploscopa* species, all feeding on ferns. The larvae of *H. gombongi* sp. nov., *H. obliqua* and *H. tonsepi* sp. nov. from Papua New Guinea are reported from *Diplazium esculentum* (Retzius in Retzius & König, 1791) Swartz, 1803 (Athyriaceae) (Miller et al. 2015, Mally et al. 2017). Another undescribed *Hoploscopa* species (sample USNM_ENT_00739239) from Papua New Guinea is reported from *Sphaerostephanos unitus* (Linnaeus, 1759) Holttum, 1794 (Thelypteridaceae) (ibid). Lastly, one undescribed *Hoploscopa* species from Borneo is reported from the fern *Dicranopteris linearis* (Burman, 1768) Underwood, 1907 (Gleicheniaceae) (Mally et al. 2017).

#### Hoploscopa albipuncta

Taxon classificationAnimaliaLepidopteraCrambidae

Léger & Nuss sp. nov.

6CD65D63-F8AC-5F2F-BBC0-A38A9DFD2A40

http://zoobank.org/26FE78DF-5D7E-4034-A730-FA5C7177466E

Figs 4 , 46 , 87

##### Material examined.

***Holotype***: ♂, with labels: “Malaysia: Sabah, Kinabalu Park H[ead]Q[uarter], | Timpohon Gate, 700m from Liwagu | Trail starting point, near Liwagu River, | 6°1'40"N, 116°32'59"E, 1760m, UV light, | 18.vi.2015, leg. T. Léger & R. Mally”; “TL335 | ♂”. Deposited in BORN.

***Paratypes***: 13 ♂, 1 ♀. Malaysia: 8 ♂ (1 with genitalia on slide TL336 ♂), same data as holotype; 3 ♂ (1 with genitalia on slide TL536 ♂), Sabah, Kinabalu National Park, Timpohon Gate, 300 m from Ligawu trail starting point, 6°1'41"N, 116°32'54"E, 1820 m, UV light, 18.vi.2015, leg. T. Léger & R. Mally (MTD); 1 ♂, Sabah, Mesilau, Kopogon, 24.ii.2006, light trap, leg. W. & M. Mey; 1 ♂ (genitalia on slide TL635 ♂), Sabah, Mesilau River, 24–25.ii.2006, light trap, leg. W. & M. Mey; 1 ♀ (DNA voucher MTD7434, genitalia on slide TL609 ♀), Sabah, Mesilau, 2000 m, 14–17.xi.2006, light trap, leg. W. Mey & K. Ebert (MFNB).

##### Other specimens examined.

1 ♂. Malaysia: 1 ♂ (DNA voucher ITBC09, genitalia on slide TL309 ♂), same data as holotype.

##### Diagnosis.

*Hoploscopa albipuncta* sp. nov. displays a basal white well-rounded spot on the forewing. In male genitalia, the dorsal margin of the valva is protruded, the juxta displays two conspicuous tips and the phallus bears an anvil-shaped cornutus. In female genitalia, the antrum is membranous and forms a rounded pouch.

##### Similar species.

No similar species known.

##### Description.

***Head.*** Antennae dorsally with brown scales. Proboscis pale yellow. Maxillary palpi brown, basally light brown. Labial palpi brown, ventrally pale yellow.

**Figures 4–18.**
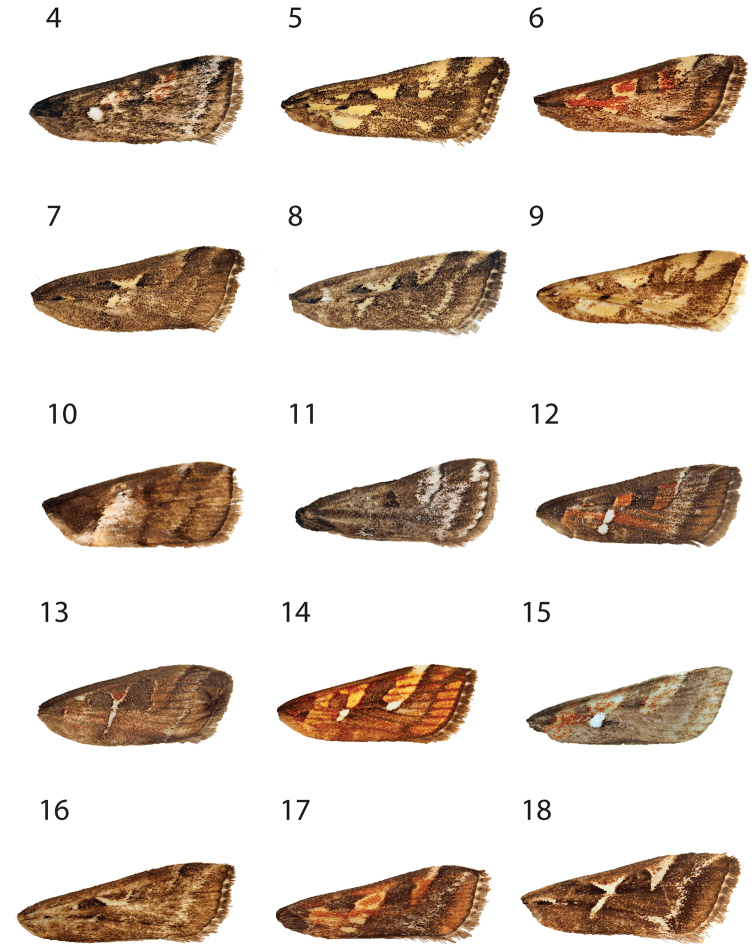
Forewing of *Hoploscopa* species. **4***Hoploscopa albipuncta* sp. nov., holotype, ♂, Malaysia, Sabah, Kinabalu Park HQ, Timpohon Gate, 700 m from Liwagu Trail starting point, near Liwagu River, 6°1'40"N, 116°32'59"E, 1760 m, 18.vi.2015 (T. Léger & R. Mally) (genitalia on slide TL335 ♂) **5***Hoploscopa sepanggi* sp. nov., holotype, ♂, Malaysia, Sabah, Mesilau Nature Resort, 6°2'43.71"N, 116°35'48.03'E, 1925 m, at lamps, 30.v.2015 (T. Léger & R. Mally) **6***Hoploscopa cynodonta* sp. nov., holotype, ♂, Malaysia, Sabah, Kinabalu Park HQ, junction Kiau View- and Pandanus Trail, 6°0'32.84"N, 116°32'14.94"E, 1690m, UV light, 07.vi.2015 (T. Léger & R. Mally) (genitalia on slide TL327 ♂) **7***Hoploscopa parvimacula* sp. nov., paratype, ♀, Sabah, Mesilau Nature Resort, 6°2'43.71"N, 116°35'48.03"E, 1925 m, at lamps, 30.v.2015 (T. Léger & R. Mally) (genitalia on slide TL312 ♀. **8***Hoploscopa kinabaluensis* sp. nov., paratype, ♀, Sabah, Kinabalu Park HQ, Timpohon Gate, 700 m from Liwagu trail starting point, near Liwagu River, 6°1'40"N, 116°32'59"E, 1760 m, UV light, 18.vi.2015 (T. Léger & R. Mally) (genitalia on slide TL326 ♀) **9***Hoploscopa luteomacula* Nuss, paratype, ♀, Indonesia, Sumatra, Barat, N-Padangpanjang, Mt Singgalang, 2100 m, 10–11.ii.1996 (A. Kallies) (genitalia on slide GU prep. Nuss 743) **10***Hoploscopa obliqua* Rotschild, abdomen missing, Papua New Guinea, Madang Province, Wanang village, 05°15'S, 145°17'E, reared from *Diplazium esculentum*, 12.vi.2007 **11***Hoploscopa niveofascia* sp. nov., holotype, ♂, Papua New Guinea, Morobe Prov., nr. Bulolo, Mt. Susu Nat. Res., 975 m, 27–28.viii. 1983, *Araucaria* For. (S. Miller) (genitalia on slide TL442 ♂) **12***Hoploscopa gombongi* sp. nov., paratype, ♂, Papua New Guinea, Yawan village, 06°10'S, 146°5'E, NG Binatang Res. Ctr. (B Gewa, J. Kua, S Sau, A. Kinibel) **13***Hoploscopa tonsepi* sp. nov., holotype, ♂, Papua New Guinea, Yawan village, 06°10'S, 146°5'E, NG Binatang Res. Ctr. (J. Valeba, J. Auga, M. Dilu, F. Philip, R. Lilip) (genitalia on slide TL655 ♂) **14***Hoploscopa marijoweissae* sp. nov., ♂, Papua New Guinea: Morobe Province, Mount Kaindi, 2350 m, 11.xii.1976 (G. F. Hevel & R. E. Dietz) (genitalia on slide TL437 ♂) **15***Hoploscopa titika* sp. nov., holotype, ♂, Sumatra-Holzweg, 25 km SSW-Pematangsiantar, Straße nach Prapat, 13.ii.1996 (A. Kallies) (genitalia on slide TL505 ♂) **16***Hoploscopa pangrangoensis* sp. nov., paratype, ♀, Indonesia, Java, Mt. Pangrange, SE Bogor, 6.30S 107.10E, 1625 m, primary forest, 16–20.ii.1996 (Siniaev & Afonin) (genitalia on slide TL627 ♀) **17***Hoploscopa isarogensis* sp. nov., paratype, ♀, Philippines, South Luzon, Mt Isarog, 13°40'N, 123°20'E, 530 m, submontane forest, at light, 22.iii.2000 (M. Nuss) (genitalia on slide TL523 ♀) **18***Hoploscopa ypsilon* sp. nov., paratype, ♀, Philippines, Luzon, Mountain Province, Chatol, 2100 m, 16–18.xi.1997 (Mey, Ebert & Nuss).

***Thorax*** (Fig. 4). Collar white. Forewing length: 9–10 mm (♂ & ♀); forewing ground colour brown; rounded snow white basal cubital spot; median discoidal stigma rhomboid to ellipsoid, dark red, edged with pale yellow; postmedian spot trapezoid, dark red, proximally pale yellow; subterminal line snow white, parallel to termen, slightly angled at M1, broader at costa; fringes brown, with white dots. Hindwing dirty pale yellow. Forelegs bronze. Midlegs brown, with tibia and tarsi segments distally white. Hindlegs brown, with tibia and tarsi segments distally white.

***Abdomen.*** Male sternum A8 posterior margin straight.

***Male genitalia*** (*N* = 5) (Fig. 46). Uncus long and slender, gently narrowing on basal half, apex tongue-shaped. Gnathos projection broad, triangular, with rounded apex. Valva ventral margin straight, dorsal margin strongly protruded dorsad, apex rounded. Juxta with base rounded, medially narrowed, apex split into two conspicuous tips. Saccus small, pointing dorsad. Phallus with sclerotised, anvil-shaped cornutus.

***Female genitalia*** (*N* = 1) (Fig. 87). Anterior apophyses with dorsal bump at posterior 1/3. Antrum forming a membranous rounded pouch. Ductus bursae of medium length, slender, straight. Corpus bursae small, globular, reticulate, with barely marked sclerotisation at thorn base. Thorn curved, with small dents pointing toward thorn apex on its inner side, glabrous on the outer side.

##### Distribution.

Known from the slopes of the Mount Kinabalu (4,095 m) on Borneo, at altitudes between 1,700 m and 2,000 m.

##### DNA barcoding.

Specimen MTD7430 from Luzon shows an K2P-distance of 4.5–4.9% with the two specimens from Borneo.

##### Etymology.

The species name *albipuncta* refers to the Latin *albus*, white, and *punctus*, forming a point.

##### Remarks.

Two female specimens from the Philippines with similar habitus and genitalia but a COI barcode divergence of 4.5–4.9% are recovered as different MOTU in the species delimitation analyses. Unfortunately, no male was available for this study, thus we refrained from describing a new species based on these two specimens.

#### Hoploscopa matheae

Taxon classificationAnimaliaLepidopteraCrambidae

Léger & Nuss sp. nov.

9377BB95-74F6-5576-B485-E607037A4B14

http://zoobank.org/D792135D-FFA9-49ED-8B76-C9802548E8E5

Figs 1 , 47 , 88

##### Material examined.

***Holotype***: ♀, with labels: “Malaysia: Sabah, Kundasang, | Kinabalu Mt. Lodge veranda, 6°0'42.15"N, 116°32'3.63"E, 1570 m, at day, 15.vi.2015, | leg. T. Léger & R. Mally”; “DNA barcoding | BC MTD Lep 3004”; “ITBC | 15”; “TL315 | ♀”. Deposited in BORN.

***Paratypes***: 1 ♂, 2 ♀. Brunei: 1 ♂ (NHMUK010923334, DNA voucher MTD8245, genitalia on slide TL730 ♂), 1 ♀ (NHMUK010923427), Bukit Retak, LP 238, GR 873804, 1365m, moss forest, 1–4.v.1989, leg. M. G. Allen & K. R. Tuck (NHMUK). Malaysia: 1 ♀ (DNA voucher MTD7426, genitalia on slide TL599 ♀), Sabah, Tawau Hills, Gelas River, 3.iii.2006, leg. W. + M. Mey (MFNB).

##### Other specimens examined.

2 ♀. Indonesia: 1 ♀ (NHMUK010923426), Java, Singolangoe, Tengger, 5000 feet, 05.1934 (F. P. A. Kalis) [abdomen missing] (NHMUK). Malaysia: 1 ♀ (NHMUK010923425, DNA voucher MTD8244 & genitalia on slide TL729 ♀), Cameron Highlands, Gunung Brinchang, 15–23.viii.1986 (G. S. Robinson) (NHMUK).

##### Diagnosis.

*Hoploscopa matheae* sp. nov. is unique in the genus by its broad pale yellow forewing fascia crossed by postmedian brown line as well as the basal and distal discoidal spots circled with pale yellow.

##### Similar species.

No similar species known.

##### Description.

***Head.*** Antennae dorsally with pale yellow scales. Proboscis dark brown. Maxillary palpi dark brown, basally and inwardly pale yellow. Labial palpi dark brown, ventro-basally pale yellow.

***Thorax*** (Fig. 1). Thorax dark brown, with two pale yellow stripes laterally and one dorsally. Collar pale yellow. Forewing length: 10 mm (♂ & ♀); forewing ground colour dark brown, with darker scales at its base; basally with a roughly defined Z-line pale yellow; basal and distal discoidal stigma of a darker brown, circled with pale yellow, median discoidal stigma therebetween dark red in some specimens; broad median triangular pale yellow patch near dorsum, connected distally by a line to postmedian triangular pale yellow patch; subterminal pale yellow line following median line shape, originating at distal 1/4 of dorsum, inwardly incurved between A1+2 and Cu2, costally diverging toward apex; subterminal field broadly marked with scales of a lighter brown; fringes dark brown, with pale yellow dots. Hindwing pale brown. Forelegs dark brown. Midlegs brown; femur and tibia distally pale yellow; tarsi pale yellow. Hindlegs dark brown, femur-tibia articulation pale yellow; tibia distally pale yellow; tarsi bronze.

***Abdomen*.** Male sternum A8 posterior margin straight.

***Male genitalia*.** (*N* = 1) (Fig. 47). Uncus broad, narrowing on distal half, with apex truncate. Gnathos without posterior projection. Valva ventral margin slightly concave, bent dorsad on apical 1/5; dorsal margin conspicuously convex; apex blunt. Juxta with base slightly quadrangular, medially narrow, apex broadly incurved. Saccus not pronounced.

***Female genitalia*** (*N* = 2) (Fig. 88). Anterior apophyses with dorsal tip pointed dorsad at posterior 1/3. Antrum sclerotisation twice as long as broad. Ductus bursae of medium length, roughly straight. Corpus bursae pear-shaped, reticulate on posterior half, sclerotised between thorn and corpus opening. Thorn straight, with small dents pointing toward thorn apex, basally with small outwardly projected extension.

##### Distribution.

Known from the Malay Peninsula, and Borneo (Brunei, Mount Kinabalu), at altitudes between 1,300 m and 1,600 m.

##### DNA barcoding.

Specimen MTD8244 from the Malay Peninsula shows an K2P-distance of 2.2% with the two specimens from Borneo. It is recovered as a distinct MOTU in the species delimitation analyses. Specimens from Kinabalu and Tawau Hills share identical COI barcodes. No COI barcode was obtained for the specimen from Brunei (MTD8245).

##### Etymology.

The species is named after Mathéa Léger, sister of the first author.

##### Remarks.

One specimen from the NHMUK collected on Mount Tengger (Indonesia, Java) in 1934 shares a similar wing pattern and thus potentially represents a conspecific specimen. Unfortunately, the abdomen of the specimen was missing. Specimen MTD8244 from the Malay Peninsula shows minor differences in wing pattern with those from Borneo: costal field, median discoidal stigma and subterminal field are dark red, and base of thorn on corpus bursae is thinner.

#### Hoploscopa sepanggi

Taxon classificationAnimaliaLepidopteraCrambidae

Léger & Nuss sp. nov.

34AD9E1D-4B17-5D41-819E-E9464386EA2B

http://zoobank.org/81756486-A524-4D2A-912E-F824C234F835

Figs 5 , 48 , 89

##### Material examined.

***Holotype***: ♂, with labels: “Malaysia, Sabah, Mesilau Nature Resort | 6°2'43.71"N, 116°35'48.03"E, 1925 m, at | lamps, 30.05.2015, leg. T. Léger & R. Mally”. Deposited in BORN.

***Paratypes***: 6 ♂, 3 ♀. Malaysia: 1 ♂ (genitalia on slide TL260 ♂) with same data as holotype except 02.vi.2015; 3 ♂ (genitalia on slide TL641 ♂ & TL338 ♂), 1 ♀ (genitalia on slide TL547 ♀), Sabah, Mesilau, logging site 400 m before entrance to Mesilau Nature Resort, 6°2'22"N, 116°35'54"E, 1930 m, UV light, 02.vi.2015, leg. T. Léger & R. Mally; 1 ♀ (genitalia TL337 on slide TL337 ♀), same data except 01.vi.2015; 1 ♂ (DNA voucher MTD7894, genitalia on slide TL671, wing preparation on slide TL237), Sabah, Kinabalu National Park, Timpohon Gate, 300 m from Ligawu trail starting point, 6°1'41"N, 116°32'54"E, 1820 m, UV light, 18.vi.2015, leg. T. Léger & R. Mally; 1 ♂ (DNA voucher MTD LEP123, genitalia on slide TL365 ♂), 1 ♀, Sabah, Mesilau, 2000 m, 14–17.xi.2006, light trap, leg. W. Mey & K. Ebert (MFNB).

##### Other specimens examined.

1 ♀. Malaysia: 1 ♀ (DNA voucher ITBC02, genitalia on slide TL302 ♀), same data as holotype.

##### Diagnosis.

*Hoploscopa sepanggi* sp. nov. bears sulfurous to pale yellow patches on the forewing forming a roughly chequered pattern. In male genitalia, the gnathos projection is slender, ca. half the length of uncus, and the juxta distal half is narrow, with a notched apex.

##### Similar species.

*Hoploscopa luteomacula*. The postmedian cubital and subterminal patch are missing or reduced to traces in *H. sepanggi* sp. nov., while they are well-marked, pale yellow in *H. luteomacula*. The fringes are brown, chequered with yellow in *H. sepanggi* sp. nov., while they are yellow in *H. luteomacula*.

##### Description.

***Head*.** Antennae dorsally striped with pale yellow and brown scales. Proboscis brown, speckled with pale yellow. Maxillary palpi brown, base and inner side pale yellow. Labial palpi brown, ventral base and inner side pale yellow.

***Thorax*** (Fig. 5). Thorax dorsally pale yellow, laterally brown. Collar pale yellow. Forewing length: 11–12.5 mm (♂), 11.5–13 mm (♀); forewing ground colour brown; basal patch and median discoidal stigma trapezoid, sulphur yellow with dark brown edges; basal and antemedian cubital patches abutting each other, quadrilateral, sulphur yellow with dark brown edges; costal field lightly marked with sulphur yellow; median dorsal patch oval, sulphur yellow; postmedian patch triangular, sulphur yellow, crossed on its middle with longitudinal brown streak; subterminal line reduced to small interspaced sulphur yellow dashes, with triangular sulphur costal spot; subterminal field broadly marked with pale yellow; fringe chequered pale yellow and brown. Hindwing pale brown. Forelegs dark brown. Midlegs brown, speckled with pale yellow. Hindlegs with femur brown; tibia pale yellow, speckled with brown, distally brown; tarsi bronze.

***Abdomen*.** Male sternum A8 posterior margin bilobed, with short, rounded lateral projections.

***Male genitalia*** (*N* = 5) (Fig. 48). Uncus long and slender, gently tapering toward tongue-shaped apex. Gnathos projection slender, ca. half the uncus length. Valva ventral margin gently bent dorsad on distal 1/3, dorsal margin conspicuously convex, apex slightly pointed. Juxta with base roughly quadrangular, medially narrowed, apex narrow, faintly notched. Saccus broad, triangular, pointing dorsad.

***Female genitalia*** (*N* = 3) (Fig. 89). Anterior apophyses with small dorsal bump at 1/3. Antrum sclerotisation twice as long as broad. Ductus bursae of medium length, slender, elbowed at 1/4 and 3/4. Corpus bursae ovoid, reticulated, diffuse sclerotisation between thorn and corpus opening, medially with faintly sclerotised band. Thorn straight, small, with tiny dents pointing toward thorn base, basally with small outwardly projected extension.

##### Distribution.

Known from the slopes of the Mount Kinabalu on Borneo, at altitudes between 1,800 m and 2,000 m.

##### Etymology.

This species is described in honour of the late Malaysian mountain guide Robbie Sepanggi who died on Mount Kinabalu during the 2015 Sabah earthquake while trying to save hikers.

#### Hoploscopa cynodonta

Taxon classificationAnimaliaLepidopteraCrambidae

Léger & Nuss sp. nov.

1B7D5887-760E-5362-9F89-DE550F77283F

http://zoobank.org/55B144AD-1AF7-4023-BC82-E5E2750AFE88

Figs 6 , 49 , 90

##### Material examined.

***Holotype***: ♂, with labels: “Malaysia, Sabah, Kinabalu Park H[ead]Q[quarters], | junction Kiau View- and Pandanus | Trail, 6°0'32.84"N, 116°32'14.94"E, | 1690 m, UV light, 07.vi.2015, | leg. T. Léger & R. Mally”; “DNA voucher | Lepidoptera | MTD 2015 | [vertically written:] no. 3066”; “TL327 | ♂”. Deposited in BORN.

***Paratypes***: 4 ♂, 2 ♀. Brunei: 2 ♂ (1 with NHMUK010923398, DNA voucher MTD8233 & genitalia on slide TL746; 1 with NHMUK010923401), 1 ♀ (NHMUK010923400), Ulu Temburong, LP 298, GR 838892, 300 m, 26–30-iv-1989, leg. M. Allen & K. Tuck (NHMUK). Malaysia: 1 ♂ (genitalia on slide TL531 ♂), Sabah, Kinabalu National Park, Timpohon Gate, 700 m from Liwagu Trail starting point, near Liwagu River, 6°1'40"N, 116°32'59"E, 1700 m, 18.vi.2015, leg. Léger & R. Mally (MTD); 1 ♀ (DNA voucher MTD7425 & genitalia on slide TL625 ♀), Sabah, Tawau Hills National Park, Headquarter, waterfall, 23–27.xi.2006, leg. W. Mey & K. Ebert (MFNB). 1 ♂ (NHMUK010923352), Sabah, Gunung Monkobo, 5.48N, 116.56E, dipterocarp forest, 945m, 14–23.viii.1987, leg. K. Tuck (NHMUK).

##### Diagnosis.

This relatively small-sized species (forewing length 8–10 mm) shows brown forewings with red markings edged with pale yellow. The median discoidal stigma is oval, red, edged with pale yellow, median cubital patch is reduced to a small streak and median dorsal patch is absent. The postmedian patch consists of a mix of red and brown scales, at costa forming triangular pale yellow patch. Male genitalia are unique in the conspicuously incurved ventral margin of the valva, medially extending into a tip pointing ventrad. In female genitalia, the corpus bursae is small, globular, and bears a crest of sclerotised acanthae between thorn and corpus opening.

##### Similar species.

*Hoploscopa isarogensis* sp. nov.; to a lesser extent *H. mallyi* sp. nov., *H. agtuuganonensis* sp. nov., *H. gracilis* sp. nov., *H. ignitamaculae* sp. nov. Median cubital and dorsal patches form with the median discoidal stigma a disrupted pale yellow band (median dorsal patch not marked in *H. mallyi* sp. nov.), and postmedian pale yellow marking at costa is reduced to a blotch in above listed species. *Hoploscopa gracilis* sp. nov. and *H. mallyi* sp. nov. are slightly larger (9–12 mm). Comparison of the male genitalia allows unequivocal separation from these species. In female genitalia, *H. isarogensis* sp. nov. displays a narrower and slightly longer ductus bursae and a larger corpus bursae with large curved thorn, while it is small and straight in *H. cynodonta* sp. nov. Female genitalia of *H. mallyi* sp. nov., *H. agtuuganonensis* sp. nov., and *H. gracilis* sp. nov. share the broad ductus bursae, the small globular corpus bursae with *H. cynodonta* sp. nov. but have a longer ductus bursae, and the sclerotisation of corpus bursae is large and diffuse.

##### Description.

***Head*.** Antennae dorsally with pale yellow scales. Proboscis brown to pale brown. Maxillary palpi brown, base and inner side pale yellow. Labial palpi brown, ventrally pale yellow.

***Thorax*** (Fig. 6). Collar white. Forewing length: 8–10 mm (♂), 8 mm (♀); forewing ground colour brown; basal longitudinal red streak basally surrounded with pale yellow U-shaped spot; costal field red; median discoidal stigma oval, red with pale yellow edges; median cubital patch forming longitudinal pale yellow rectangle; postmedian patch red, with basal edge pale yellow, distally abutted with pale yellow triangular costal patch; postmedian line costally marked; postmedian suffusion of pale yellow and pale brown scales, distally edged by pale yellow subterminal line; subterminal field marked with red; fringe chequered pale yellow and brown. Hindwing pale brown. Forelegs and midlegs brown. Hindlegs with femur brown; tibia pale yellow, distally brown; tarsi bronze speckled with pale yellow.

***Abdomen*.** Male sternum A8 posterior margin forming two conspicuous triangular tips, with short, rounded lateral projections.

***Male genitalia*** (*N* = 2) (Fig. 49). Uncus medially slightly widened, narrowing toward apex, apex duck beak-shaped, medio-ventrally with a bump and two ridges. Gnathos projection roughly triangular, ca. 1/4 of uncus length, with rounded apex. Valva ventral margin with basal half sclerotised, at 1/4 conspicuously incurved into a semi-circle, protruded medio-ventrally into a tooth-like tip pointing ventrad; valva dorsal margin convex, apex truncate. Juxta with base rounded, medially narrowed, apical half laterally protruded, apex broad, not sclerotised. Saccus quadrangular, conspicuously produced anterad.

***Female genitalia*** (*N* = 1) (Fig. 90). Posterior apophyses bent ventrad. Anterior apophyses with small dorsal bump at 1/3. Antrum sclerotisation twice as long as broad, barely sclerotised. Ductus bursae of medium length, broad, with a narrow loop before corpus bursae. Corpus bursae globular, reticulated, with crest of sclerotised acanthae between thorn and corpus opening. Thorn straight, slightly incurved at apex, with small dents pointing toward thorn base.

##### Distribution.

Known on Borneo from Brunei to the Mount Kinabalu, the Mount Monkobo (1,759m) and the Tawau Hills in Sabah (Malaysia), at altitudes between 300 and 1,700 m.

##### DNA barcoding.

Specimens MTD LEP3066 from Mount Kinabalu and MTD7425 from Tawau Hills differ by 0.4%. The nearest neighbour is *H. isarogensis* sp. nov. from the Philippines (K2P-dist = 2.3–3%).

##### Etymology.

Formed by apposition of the Greek words *cyno*-, of the dog, and *odous*, tooth, referring to the conspicuous tooth-like extension on the ventral margin of the valva in male genitalia.

#### Hoploscopa parvimacula

Taxon classificationAnimaliaLepidopteraCrambidae

Léger & Nuss sp. nov.

98A953F3-2D91-5814-9FC7-F5C328DD3BEC

http://zoobank.org/4E445DD3-8C71-40DE-9EDB-870935206EDE

Figs 7 , 50 , 91

##### Material examined.

***Holotype***: ♂, with labels: “Malaysia, Sabah, Mesilau, logging | site 400m before entrance to Mesilau | Nature Resort, 6°2'21.97"N, 116°35'54.12"E, 1930m, UV light, | 02.vi.2015, leg. T. Léger & R. Mally”; “DNA voucher | Lepidoptera | MTD2016 | [vertically written:] n°. 3196 ”; “TL510 | ♂”. Deposited in BORN.

***Paratypes***: 7 ♂, 2 ♀. Malaysia: 1 ♂ (DNA voucher MTD7888 & genitalia on slide TL552 ♂) with same data as holotype, 2 ♂ (DNA vouchers MTD7884 & MTD7885, genitalia on slides TL673 ♂ & TL261 ♂), 1 ♀ (DNA voucher MTD7883 & genitalia on slide TL551 ♀) with same data as holotype except 02.vi.2015; 1 ♂ (DNA voucher MTD7886, genitalia on slide TL537 ♂), Sabah, Mesilau Nature Resort, 6°2'44"N, 116°35'48"E, 1925 m, at lamps, 30.v.2015, leg. T. Léger & R. Mally; 1 ♀ (DNA voucher ITBC12, DNA barcoding voucher BC MTD LEP3003, genitalia on slide TL312 ♀), same data except 01.vi.2015; 2 ♂ (DNA vouchers MTD7895 & MTD7896, genitalia on slides TL550 ♂ & TL549 ♂), Sabah, Kinabalu National Park, Timpohon Gate, 300 m from Liwagu trail starting point, 6°1'41"N, 116°32'54"E, 1820 m, UV light, 18.vi.2015, leg. T. Léger & R. Mally; 1 ♂ (DNA voucher MTD7890, genitalia on slide TL672 ♂), Sabah, Kundasang, Kinabalu Mountain Lodge veranda, 6°0'42"N, 116°32'4"E, 1570 m, at light, 18.vi.2015, leg. T. Léger & R. Mally (MFNB).

##### Diagnosis.

*Hoploscopa parvimacula* sp. nov. displays brown forewings with reduced pale yellow markings. The median discoidal stigma is crescent-shaped, and the median cubital patch forms a streak not connected to the median discoidal stigma. The postmedian patch and postmedian area are faintly marked with pale yellow. In male genitalia, the gnathos projection is distally spatula-shaped, reaching ca. 2/3 of the uncus length, and the juxta displays blunt lateral projections and a truncate apex.

##### Similar species.

*Hoploscopa kinabaluensis* sp. nov., *H. pangrangoensis* sp. nov. The forewings of *H. kinabaluensis* sp. nov. display more contrasting postmedian patch and subterminal line, and a subterminal field suffused with pale yellow. In *H. pangrangoensis* sp. nov., the median cubital patch is almost absent and the subterminal line is more strongly pronounced. In male genitalia, base of gnathos projection is narrower and the cornutus displays a narrow bump at apex in *H. kinabaluensis* sp. nov. The finger-like gnathos projection of *H. pangrangoensis* sp. nov. is narrower than that of *H. parvimacula* sp. nov. and reaches only half the uncus length. In female genitalia, ductus bursae of *H. kinabaluensis* sp. nov. is twice as long as in other species, with a loop on its middle and a large bow before corpus bursae. In *H. pangrangoensis* sp. nov., colliculum is membranous with longitudinal sclerotised lines, corpus bursae is larger and thorn is smaller.

##### Description.

***Head*.** Antennae dorsally striped with brown and bronze scales. Proboscis pale yellow, speckled with pale brown. Maxillary palpi dark brown, base and inner side pale yellow. Labial palpi dark brown, ventro-basally pale yellow.

***Thorax*** (Fig. 7). Collar pale yellow. Forewing length: 9–10 mm (♂ & ♀); forewing ground colour brown; basal dark brown dash, distally pale yellow; basal and distal discoidal stigmata dark brown; median patches Y-shaped, pale yellow, disrupted with brown at vein, edged basally and distally with dark brown; postmedian patch of a lighter brown, with pale yellow triangular costal spot distally edged with dark brown; subterminal line pale yellow, distally dark brown; fringe brown, with pale yellow dots. Hindwing pale brown. Forelegs brown. Midlegs with femur brown; tibia pale yellow, speckled with brown; tarsi bronze. Hindlegs with femur brown; tibia pale yellow, speckled with brown; tarsi pale yellow to pale brown.

***Abdomen*.** Male sternum A8 posterior margin bilobed, with short, rounded lateral projections.

***Male genitalia*** (*N* = 8) (Fig. 50). Uncus medially broadened, narrowing on distal half, apex slightly obtuse. Gnathos projection ca. 2/3 of uncus length, spatula-shaped. Valva ventral margin straight, gently bent dorsad on distal 1/4, dorsal margin convex, apex rounded or slightly pointed. Juxta slender, base roughly quadrangular, apex truncate. Saccus small, pointing dorsad.

***Female genitalia*** (*N* = 2) (Fig. 91). Anterior apophyses with dorsal bump at posterior 1/3. Antrum sclerotisation twice as long as broad. Ductus bursae short and straight, slightly bent before corpus bursae. Corpus bursae pear-shaped, posterior half reticulated, anterior half membranous, with diffuse sclerotisation between thorn and corpus opening, medially with faintly marked sclerotised band. Thorn short and straight, with small dents pointing toward thorn apex.

##### Distribution.

Known from Mount Kinabalu on Borneo, at altitudes between 1,550 and 1,950 m.

##### DNA barcoding.

Sample MTD8229 of *H. parvimacula* sp. nov. shows a K2P-dist of 3.1–3.4% with samples from Borneo and is recovered as separate MOTU.

##### Etymology.

Refers to the Latin *parvus*, for small, and *macula*, for spot, referring to the Y-shaped median markings that are smaller than in other similar species.

##### Remarks.

The specimen with DNA voucher MTD8229 belongs to a series of females from the Malay Peninsula, which are morphologically similar to *H. parvimacula* sp. nov. and deposited at NHMUK. Since no males of the same series were available for investigations, we restrained from drawing further conclusions here.

#### Hoploscopa kinabaluensis

Taxon classificationAnimaliaLepidopteraCrambidae

Léger & Nuss sp. nov.

D1CC3B28-ACEA-5B25-BDC2-1B67FB8FA4DE

http://zoobank.org/F75F92C1-7C04-4BE9-A602-0FE822BF66D2

Figs 8 , 51 , 92

##### Type material.

***Holotype***: ♀, with labels: “Malaysia, Sabah, Kundasang | Kinabalu Mt. Lodge veranda, | 6°0'42.15"N, 116°32'3.63"E, | 1570m, at light, 16.vi.2015, | leg. T. Léger & R. Mally”; “DNA voucher | Lepidoptera | MTD 2015 | [vertically written:] no. 3064”; “TL325 ♀”. Deposited in BORN.

***Paratypes***: 3 ♂, 4 ♀. Malaysia: 1 ♂ (DNA voucher MTD7889, genitalia on slide TL667 ♂), same data as holotype; 1 ♂ (DNA voucher MTD7893 & genitalia on slide TL342 ♂), 1 ♀ (DNA voucher MTD LEP3065 & genitalia on slides TL326 ♀), Sabah, Kinabalu National Park, Timpohon Gate, 700 m from Liwagu trail starting point, near Liwagu River, 6°1'40"N, 116°32'59"E, 1760 m, UV light, 18.vi.2015, leg. T. Léger & R. Mally; 1 ♂ (DNA voucher MTD7897, genitalia on slide TL341 ♂), Sabah, Kinabalu National Park, Timpohon Gate, 300 m from Liwagu trail starting point, 6°1'41"N, 116°32'54"E, 1820 m, UV light, 18.vi.2015, leg. T. Léger & R. Mally; 1 ♀ (DNA voucher MTD7882, TL666 ♀), Sabah, Kinabalu National Park Headquarters, junction Kiau View- and Pandanus Trail, 6°0'32.84"N, 116°32'15"E, 1690 m, UV light, 07.vi.2015, leg. T. Léger & R. Mally; 1 ♀ (DNA voucher MTD7887, genitalia on slide TL662 ♀), Sabah, Kundasang, road 200 m before Kinabalu Mt. Lodge, 6°0'37"N, 116°32'4"E, 1535m, UV light, 03.vi.2015, leg. T. Léger & R. Mally (MTD).

##### Other specimens examined.

1 ♂, 2 ♀. Malaysia: 1 ♂ (DNA voucher MTD7892, genitalia on slide TL546 ♂), 1 ♀ (DNA voucher MTD7891 & genitalia on slide TL674 ♀), Sabah, Kinabalu National Park, Timpohon Gate, 700 m from Liwagu trail starting point, near Liwagu River, 6°1'40"N, 116°32'59"E, 1760 m, UV light, 18.vi.2015 (T. Léger & R. Mally); 1 ♀ (NHMUK 010923435, DNA voucher MTD8232 & genitalia on slide TL745 ♀), Sabah, Gunung Monkobo, dipterocarp forest, 975m, 5.48N, 116.56E (7–13.viii.1987) (NHMUK).

##### Diagnosis.

The forewing median markings form an interrupted pale yellow Y, and postmedian patch and subterminal line are pale yellow, well-marked. In male genitalia, the gnathos projection is ca. 2/3 of uncus length, spatula-shaped, broadening toward apex, and the cornutus displays a small but conspicuous bump. Female genitalia show a long ductus bursae broadly curved twice and a large pear-shaped corpus bursae.

##### Similar species.

*Hoploscopa parvimacula* sp. nov. (q.v.), *H. brunnealis*, *H. danaoensis* sp. nov., *H. metacrossa*. These species share similar forewing markings with *H. kinabaluensis* sp. nov. Male genitalia of these species show a gnathos projection larger at base, of constant width, reaching ca. half of uncus length, and a cornutus lacking the apical bump. In female genitalia, the shorter ductus bursae is nearly straight.

##### Description.

***Head*.** Antennae dorsally striped with pale yellow and bronze scales. Proboscis brown speckled with pale yellow. Maxillary palpi brown, base and inner side pale yellow. Labial palpi brown, ventral base and inner side pale yellow.

***Thorax*** (Fig. 8). Thorax dorsally pale yellow, laterally brown. Collar pale yellow. Forewing length: 9.0–10.5 mm (♂), 10–11 mm (♀); forewing ground colour brown; basal dark brown dash, distally pale yellow; median discoidal stigma and median cubital patch forming together a pale yellow-coloured Y, disrupted with brown at vein, edged basally and distally with dark brown; postmedian patch roughly triangular, pale yellow, invaded with brown, edged with dark brown; subterminal line thick, pale yellow; fringe chequered pale yellow and brown. Hindwing pale brown. Forelegs brown. Midlegs with femur brown; tibia brown, inwardly pale yellow; tarsi bronze. Hindlegs with femur brown; tibia pale yellow, speckled with brown; tarsi bronze, speckled with brown.

***Abdomen*.** Male sternum A8 posterior margin broadly indented, with short, rounded lateral projections.

***Male genitalia*** (*N* = 4) (Fig. 51). Uncus broad, gently narrowing on distal half, apex obtuse. Gnathos projection ca. 2/3 of uncus length, narrow at base, widening toward apex, apex spatula-shaped. Valva ventral margin straight, gently bent dorsad on distal 1/3, dorsal margin convex, apex rounded or slightly pointed. Juxta slender, base roughly quadrangular, apex slightly incurved. Saccus small, pointing dorsad. Phallus with elongated cornutus, at posterior end with narrow bump pointing dorsad.

***Female genitalia*** (*N* = 5) (Fig. 92). Anterior apophyses with tip or bump at posterior 1/3. Antrum sclerotisation ca. twice as long as broad. Ductus bursae long, slender, with one loop, curved before corpus bursae. Corpus bursae conspicuously pear-shaped, reticulated, with sclerotisation between thorn and corpus opening and faintly marked sclerotised band medially. Thorn long, slightly curved, with small dents pointing toward thorn apex.

##### Distribution.

Known from the slopes of Mount Kinabalu and Mount Monkobo on Borneo, from altitudes between 975 m and 1,850 m.

##### DNA barcoding.

Two MOTUs diverging by 1.7–2.2% are recovered in *H. kinabaluensis* sp. nov. These two lineages are found in sympatry at Mount Kinabalu.

##### Etymology.

The species name *kinabaluensis* refers to Mount Kinabalu on Borneo, where the species occurs.

##### Remarks.

The two barcode lineages show no differences in wing pattern and female genitalia. Only minor differences are found in male genitalia, with the apex of the gnathos projection slightly narrower and the basal margin of the juxta slightly incurved in the second lineage. Since second MOTU is only represented by three specimens including only one male, further investigations with a greater number of specimens is needed.

#### Hoploscopa luteomacula

Taxon classificationAnimaliaLepidopteraCrambidae

Nuss, 1998

2BA90486-E0D7-5339-AB37-858E3F3740C8

Figs 9 , 52 , 93

##### Material examined.

***Holotype***: ♂, with labels: “Holotypus”; “Sumatra, Barat | N-Padangpanjang | Mt. Singgalang 2100m | 10–11.ii.1996, L[icht]F[ang] [light trap] | leg. A. Kallies”; “HOLOTYPE | Hoploscopa luteomacula | det. Nuss, 1996”; “GU 744 | prep. Nuss 1996”; “Coll. M. Nuss | Geschenk 2000 | Museum für Tier- | kunde Dresden”. Deposited in MTD.

***Paratype***: 2 ♀. Indonesia: 1 ♀ (genitalia on slide GU 743, DNA barcode BC MTD 01419), same data as holotype; 1 ♀ (genitalia in capsule under specimen), Sumatra, Holzweg 2, 25km SSW-Pematangsiantar, Strasse nach Prapat [road to Prapat], 25.x.1989, leg. E. W. Diehl (MTD).

##### Other material examined.

1 ♂, 1 ♀. Brunei: ♀ (NHMUK010923403, DNA voucher MTD8234 & genitalia on slide TL747 ♀), Ulu Temburong, LP 298, GR 838892, 300m, 26–30.iv.1989 (M. G. Allen & K. R. Tuck). Malaysia: 1 ♂ (DNA voucher MTD LEP3195 & genitalia on slide TL525 ♂), Sabah, Kundasang, road 200m before Kinabalu Mt. Lodge, 6°0'37.38"N, 116°32'0.35"E”, 1535m, UV light, 03.vi.2015, leg. T. Léger & R. Mally (MFNB).

##### Diagnosis.

*Hoploscopa luteomacula* displays broad pale yellow patches in the forewing. In male genitalia, the uncus is rectangular with a truncate apex, the gnathos forms a tongue-shaped projection ca. 1/4 of the uncus length, the juxta is medially conspicuously narrowed and displays a duck-shaped apex.

##### Similar species.

*Hoploscopa sepanggi* (q.v.).

##### Description.

***Head*.** Antennae dorsally pale yellow. Proboscis pale yellow. Maxillary palpi brown, base and inner side pale yellow. Labial palpi brown, ventral base and inner side pale yellow.

***Thorax*** (Fig. 9). Thorax brown, dorsally pale yellow. Collar pale yellow. Forewing length: 9–11 mm (♂ & ♀); forewing ground colour brown, with markings pale yellow; basal patch large, rhomboid, slightly encroached with brown near dorsum; small basal discoidal spot; median discoidal stigma trapezoid; costal field pale yellow, speckled with brown; median cubital and dorsal patches rhomboid; postmedian patch quadrangular to elliptic, dorsally invaded with brown; postmedian cubital patch rhomboid; subterminal costal patch triangular; subterminal field more or less broadly marked with pale yellow; fringes pale yellow, sometimes with tiny brown dots, apex brown. Hindwing pale yellow to pale brown. Forelegs femur pale yellow; tibia brown, inner side pale yellow; tarsi pale yellow speckled with bronze. Midlegs with femur pale yellow; tibia and tarsi brown. Hindlegs pale yellow.

***Male genitalia*** (*N* = 1) (Fig. 52). Uncus large, rectangular, slightly narrowed at apical 1/4, apex truncate. Gnathos projection tongue-shaped, ca. 1/3 of uncus length. Valva ventral margin nearly straight, dorsal margin convex, apex blunt. Juxta with large rounded base, medially conspicuously narrowed, apex duck beak-shaped. Saccus small, pointing dorsad.

***Female genitalia*** (*N* = 1) (Fig. 93). Anterior apophyses with dorsal bump at posterior 1/3. Antrum sclerotisation reduced to sclerotised ring. Ductus bursae short, nearly straight. Corpus bursae large, pear-shaped, reticulated on posterior half, anterior half membranous, with faint sclerotisation between thorn and corpus opening. Thorn nearly straight, with dents pointing toward thorn apex, basally with small outwardly projected extension.

##### Distribution.

Known from Sumatra (Indonesia) at altitudes between 1,200 m and 2,100 m.

##### DNA barcoding.

Specimens MTD8234 and MTD LEP3195 from Borneo show an K2P-distance of 2.5–4.4% with the specimen from Sumatra and are recovered as a distinct MOTU.

##### Remarks.

Deep barcode divergence between morphologically similar specimens from Borneo and Sumatra might represent phylogeographic variability or distinct species. Additional material from both regions and a broader set of characters is needed to test these hypotheses.

#### Hoploscopa obliqua

Taxon classificationAnimaliaLepidopteraCrambidae

(Rothschild, 1915)

42BDB9BB-2EAB-56F5-AB88-95850222B8AC

Figs 10 , 53 , 94

##### Material examined.

***Holotype***: ♂, with labels: “Holo- | type” [round label, red ringed]; “Utakwa R[iver]., | Dutch N[ew]. Guin[ea]., | 3000 f[ee]t., Jan[uary]. 1913. | A.F.R. Wollaston.”; “437”; “Eudorina | obliqua | Type Rotsch[ild].” [handwritten]; “♂ | Pyralidae | Brit[ish].Mus[eum]. | Slide N°. | 20252”; "NHMUK 010923283” [barcode appended]. Deposited in NHMUK.

##### Other specimens examined.

Papua New Guinea: 2 specimens [abdomens lost] (1 with DNA voucher “USNM ENT 00665932”, 1 with DNA voucher “USNM ENT 00514731”), 1 ♀ (DNA voucher “USNM ENT 00514750”, genitalia on slide TL656 ♀), Madang Province, Wanang village, 05°15'S, 145°17'E, 1700 m, reared from *Diplazium esculentum*, 12.vi.2007, leg. Auga, Molem, Tamtiai, Lilip, Ibalim, Posman, Rimandai, Brus, Novotny, Hrcek (USNM).

##### Diagnosis.

The antennae striped with white and the antemedian oblique white streak extended distally into white suffusion in the forewing are unique to this species. In male genitalia, the gnathos projection forms a broad plate with a small apical tip. In female genitalia, the antrum is membranous, the ductus bursae is long and corpus bursae is densely covered with erect papillae on one half.

##### Similar species.

No similar species known.

##### Description.

***Head*.** Antennae dorsally striped with brown and white. Proboscis brown. Maxillary palpi brown, base pale brown, apex white. Labial palpi brown with white apex.

***Thorax*** (Fig. 10). Collar white. Forewing length: 8–9 mm (♂ & ♀); forewing ground colour brown; basal area of a darker brown; antemedian oblique thick white streak running from dorsum to upper margin of cell, distally extended into white suffusion; postmedian patch reduced to costal white blotch; postmedian line of a darker brown at costa; barely marked subterminal white line incurved inwards at CuA2, running up to M2; fringes pale brown. Hindwing pale brown. Fore- and midlegs brown, tibia and tarsi segments distally white. Hindlegs brown.

***Male genitalia*** (*N* = 1) (Fig. 53). Uncus medially conspicuously widened, narrowed at apical 1/4, apex spatulate. Gnathos projecting into a broad spatula-shaped plate with small apical tip on its middle. Valva ventral margin gently bent dorsad on distal 1/3, dorsal margin convex, apex pointed. Juxta slender, with base rounded, apex obtuse. Saccus small, pointing dorsad. Phallus with club-shaped cornutus.

***Female genitalia*** (*N* = 1) (Fig. 94). Anterior apophyses bent ventrad at 1/3. Antrum membranous. Ductus bursae long, straight. Corpus bursae globular, with one half reticulated, one half with erect papillae, with sclerotised bump on each side of thorn. Thorn straight, broad at base, tapering at mid-length, with small dents pointing toward thorn apex.

##### Distribution.

Known from the Papua (Indonesia) and Madang Provinces (Papua New Guinea) on New Guinea, at altitudes between 1,000 m and 1,700 m.

##### Phylogenetic relationships.

*Hoploscopa niveofascia* sp. nov. is recovered as sister group in the ML analysis of the COI barcode (BS = 95).

##### Remarks.

Nuss (1998) transferred this species from *Eudorina* to *Hoploscopa*.

#### Hoploscopa niveofascia

Taxon classificationAnimaliaLepidopteraCrambidae

Léger & Nuss sp. nov.

C17C0F5F-4055-574B-88F9-6843DAF341BB

http://zoobank.org/4112C427-AF2B-4CAE-BDB3-E94033B8E8C2

Figs 11 , 56

##### Material examined.

***Holotype***: ♂, with labels: “Papua New Guinea | Morobe Prov[ince]., n[ea]r. Bulolo | Mt. Susu Nat[ional]. Res[erve]., 975m | 27–28 Aug.1983, S. Miller | UV Lite, Araucaria For[est]”; “DNA voucher | Lepidoptera | MTD2016 | [vertically written:] no. 3162”; “TL442 | ♂”. Deposited in USNM.

##### Diagnosis.

*Hoploscopa niveofascia* sp. nov. displays snow white postmedian patch, subterminal line and well-marked marginal spots on the forewing. In male genitalia, the uncus is broad, with straight lateral margin and a blunt apex. Female genitalia not known.

##### Similar species.

*Hoploscopa diffusa*. *Hoploscopa niveofascia* sp. nov. shares with *H. diffusa* the white markings of the forewing. However, postmedian patch of *H. diffusa* is broadly marked with brown, marginal spots are reduced to small dots, and fringes exhibit white spots, while fringes are brown in *H. niveofascia* sp. nov.

##### Description.

***Head*.** Antennae dorsally dark brown. Proboscis white. Maxillary palpi dark brown, basally pale brown. Labial palpi dark brown, ventro-basally white.

***Thorax*** (Fig. 11). Thorax dark brown with mesodorsal transversal white line. Collar white. Forewing length: 9.5 mm; forewing ground colour dark brown; basal area without marked patches, white scales scattered near dorsum; median discoidal stigma darker coloured, rectangular, basally and distally thinly edged with white; postmedian patch diffuse, white; postmedian line marked on costal half; subterminal line thick, zigzagging, disrupted distally at M1; margin with large white spots; fringes bronze. Fore- and midlegs dark brown, with tibia and tarsi segments distally white. Hindlegs brown to bronze.

***Abdomen.*** Male sternum A8 posterior margin straight.

***Male genitalia*** (*N* = 1) (Fig. 56). Uncus broad with straight lateral margin, apex truncate, medially slightly incurved. Gnathos reduced to thin band without posterior projection. Valva ventral margin bent dorsad on distal half, dorsal margin slightly convex, apex pointed. Juxta elongated, with base rounded, medially narrowing, apex blunt. Saccus small, pointing dorsad.

***Female genitalia*** Not known.

##### Distribution.

Known from Mount Susu (975 m) in the Morobe Province (Papua New Guinea).

##### Phylogenetic relationships.

*Hoploscopa obliqua* is recovered as sister group in the ML analysis of the COI barcode (BS = 95).

##### Etymology.

From the Latin *niveus*, snowy, and *fascia*, band, referring to the snow-white markings on the forewing.

#### Hoploscopa gombongi

Taxon classificationAnimaliaLepidopteraCrambidae

Léger & Nuss sp. nov.

523587A7-4399-56D0-853A-2B9C26297F6F

http://zoobank.org/74AD0BB2-8718-42BA-88A3-0C6236302D18

Figs 12 , 54 , 96

##### Material examined.

***Holotype***: ♂, with labels: [exuvia pinned under the specimen] “Sp. PYRA-106 | YS.2H.3826 | CATY 001 L[ength] 9mm | YC 29975 Leaf M [circled] Y | Roll [circled] - Tie - Chew - Skel | 10-SEP-2012 [handwritten]”; “Papua New Guinea | Yawan village | 06°10'S, 146°5'E [1700m] | L[e]g[i]t B Gewa, J. Kua, | S Sau, A. Kinibel | NG Binatang Res[earch]. C[en]t[e]r.”; “USNM ENT: PNG | Madang Ecology Project | [barcode] | 00739216” [DNA voucher]; “DNA 2013”; “TL | 653 ♂”. Deposited in USNM.

***Paratypes***: 1 ♂, 2 ♀. Papua New Guinea: 1 ♂ (“YC29970”, DNA voucher “00739200”), 1 ♀ (“YC29974”, DNA voucher “00739238”, genitalia on slide TL654 ♀), same data as holotype except “L=19mm”, “Chew” (♂), “Leaf Y”, “L=14mm”, “Tie” (♀), leg. M. Rimandai, S. Sau, A. Kinibel, M. Jimbudo; 1 ♀ (“YC28238”, DNA voucher “00739199” & MTD7872, genitalia on slide TL657 ♀), same data as holotype except “Chew”, “L=15mm”, 11.ix.2012, leg. M. Rimandai, S. Sau, A. Kinibel, M. Jimbudo (USNM).

##### Diagnosis.

The well-marked median cubital and dorsal snow-white patches on the forewing segregate this species from its congeners. Median discoidal stigma is trapezoid, reddish brown, postmedian patch is reddish brown, distally edged by thin white streak. In male genitalia, the narrow uncus-tegumen connection and the broadly indented uncus apex is unique to this species. In female genitalia, papillae anales are thick, not connecting dorsally and ventrally, corpus bursae is small, globular, with a long straight thorn.

##### Similar species.

No similar species known.

##### Description.

***Head*.** Antennae dorsally brown. Proboscis pale brown. Maxillary palpi dark brown, base and inner side pale yellow to light brown. Labial palpi dark brown, ventro-basally pale yellow.

***Thorax*** (Fig. 12). Collar white. Forewing length 10–11 mm (♂), 9–10 mm (♀); forewing ground colour dark brown; cubital reddish brown fascia running from basal to postmedian area; basal and distal discoidal patches of a darker brown; rhombical reddish brown median discoidal stigma therebetween, basally and distally thinly edged with pale yellow; median cubital and dorsal patches white, elongated, slightly disrupted at 1A+2A; postmedian roughly quadrangular reddish brown patch, crossed with brown lines, with slender white streak abutting dorsally, running up to costa; postmedian line thin, marked on costal half; postmedian fascia white, speckled with brown; subterminal line thin, white, running more or less straight from dorsum distal 1/4 to apex; subterminal field faintly marked with reddish brown; fringes brown, with pale yellow dots. Hindwing pale yellow, slightly darker at apex. Legs brown, tibia distally pale yellow, tarsi brown to pale brown.

***Abdomen*.** Male sternum A8 posterior margin bilobed.

***Male genitalia*** (*N* = 1) (Fig. 54). Uncus slender, entirely sclerotised, forming narrow connection to tegumen, narrowed on its middle, apex large, broadly indented. Gnathos reduced to barely sclerotised band. Tegumen arms dorso-posteriorly not fused. Valva slender, ventral margin nearly straight, gently bent dorsad on distal 1/4, dorsal margin convex, valva apex pointed. Juxta with base rounded, medially slightly narrowed with weakly sclerotised edges, apex broadly rounded, flanked on each side with sclerotised bump covered with setae. Saccus not pronounced. Phallus apically with sclerotised spine.

***Female genitalia*** (*N* = 2) (Fig. 96). Papillae anales thick, dorsally and ventrally not connected. Posterior apophyses bent dorsad. Anterior apophyses widened at posterior 1/3, with tip pointed dorsad. Antrum sclerotisation as long as broad. Ductus bursae long, more or less straight. Corpus bursae small, globular, reticulated, with sclerotisation between thorn and corpus opening. Thorn straight, with small dents pointing toward thorn base, basally with small outwardly projected extension.

##### Distribution.

Known from Yawan village (1,700 m) in the Eastern Highlands Province (Papua New Guinea).

##### Biology.

The moths were reared from the fern *Diplazium esculentum* (Retzius in Retzius & König, 1791) Swartz, 1803 (Athyriaceae) (S. Miller, C. Redmond & T. Whitfield, pers. comm.).

##### Etymology.

The species epithet *gombongi* comes from “gombong”, the name for fern in the Yau language (https://www.ethnologue.com/language/yuw), referring to the larval host plant. This name was suggested by Vojtěch Novotný and Gibson Mayiah.

#### Hoploscopa tonsepi

Taxon classificationAnimaliaLepidopteraCrambidae

Léger & Nuss sp. nov.

EE5BA633-CC6B-5F92-AC6E-D41FC0891CC3

http://zoobank.org/B0664603-AEC3-4D7B-8A99-146A6359A88C

Figs 13 , 55 , 101

##### Material examined.

***Holotype***: ♂, with labels: [exuvia in a capsule pinned under the specimen] “Sp. PYRA-106 | YP.4B.4041 | CATY 002 L 11 mm | YC 30471 Leaf M [circled] Y | Roll - Tie - Chew [circled] - Skel | 13 OCT 2012 [handwritten]”; “Papua New Guinea | Yawan village | 06°10'S, 146°5'E [1700m] | L[e]g[i]t J. Valeba, J. Auga | M. Dilu, F. Philip, R. Lilip | NG Binatang Res[earch]. C[en]t[e]r.”; “USNM ENT: PNG | Madang Ecology Project | [barcode] | 00739227” [DNA voucher]; “DNA 2013”; “TL | 655 ♂”. Deposited in USNM.

***Paratypes***: 2 ♂, 1 ♀. Papua New Guinea: 1 ♂ (“YC30467”, DNA voucher MTD7874 & “00739207”, genitalia on slide TL661 ♂), same data as holotype except “CATY 001, L = 15 mm”; 1 ♂ (“YC30472”, DNA voucher “00739208”), same data as holotype except “CATY 001, L = 15 mm”, leg. B. Gewa, J. Kua, S. Sau, A. Kinibel; 1 ♀ (“YC30473”, DNA voucher “00739234” & MTD7873, genitalia on slide TL658 ♀), same data as holotype except “L = 10 mm” “Roll”, leg. B. Gewa, J. Kua, S. Sau, A. Kinibel (USNM).

##### Diagnosis.

*Hoploscopa tonsepi* sp. nov. displays a triangular reddish brown median discoidal stigma with white edges, extending toward dorsum into narrow white cubital streak. In male genitalia, the two-armed uncus with two small tips on its middle is unique to this species. In female genitalia, the absence of posterior apophyses, the short ductus bursae and the small corpus bursae without thorn are atypical for the genus and observed only in this species.

##### Similar species.

No similar species known.

##### Description.

***Head*.** Antennae dorsally striped with brown and bronze scales. Proboscis pale yellow, brown at base. Maxillary palpi dark brown, base and inner side pale yellow to light brown. Labial palpi dark brown, ventro-basally pale yellow.

***Thorax*** (Fig. 13). Collar pale yellow. Forewing length: 9–10 mm (♂ & ♀); forewing ground colour brown, broadly suffused with reddish brown; basal oblique thin white streak; cubital reddish brown fascia running from basal to postmedian area; basal and distal discoidal patches of a darker brown, basally and distally thinly edged with white; median discoidal stigma reddish brown, with V-shaped white edge, together with narrow white cubital streak forming a Y; postmedian patch roughly triangular, reddish brown, crossed with brown lines, abutted with pale yellow blotch at costa; postmedian line broad, marked on costal half; postmedian suffusion faintly marked, pale yellow; subterminal line pale yellow, diffuse, running more or less straight from dorsum distal 1/4 to apex; subterminal field marked with reddish brown; fringes brown, with pale yellow dots. Hindwing pale brown, darker at apex. Forelegs dark brown; tibia dark brown, distally pale yellow; tarsi pale yellow. Midlegs with femur dark brown; tibia pale yellow, speckled with brown, distally brown; tarsi pale yellow. Hindlegs brown; tibia and tarsi segments distally pale yellow.

***Abdomen*.** Male sternum A8 posterior margin bilobed.

***Male genitalia*** (*N* = 2) (Fig. 55). Uncus broad, entirely sclerotised, on distal half extending into two arms with rounded tip, with small dent on inner side, margin between arms with two small tips on its middle. Gnathos projection plump, ca. 2/3 of uncus length. Valva slender, ventral margin nearly straight, dorsal margin slightly convex, apex pointed. Juxta with base roughly rounded, medially narrowed, apex blunt, slightly incurved. Saccus slightly quadrangular. Coecum penis reduced to a short protrusion.

***Female genitalia*** (*N* = 1) (Fig. 101). Papillae anales thick, dorsally and ventrally not connected. Posterior apophyses absent. Anterior apophyses without bump at posterior 1/3. Antrum sclerotisation short. Ductus bursae short, slender, straight. Corpus bursae very small, ovoid, covered with tiny papillae. Corpus sclerotisation and thorn absent.

##### Distribution.

Known from the Yawan village (1,700 m) in the Eastern Highlands Province (Papua New Guinea).

##### Biology.

The moths were reared from *Diplazium esculentum* (Retzius in Retzius & König, 1791) Swartz, 1803 (Athyriaceae) (S. Miller, C. Redmond, & T. Whitfield, pers. comm.).

##### Etymology.

Named after Tonsep, the village leader of Yawan in Papua New Guinea, who locally conducted the project on which the larvae were collected and reared to adults.

#### Hoploscopa marijoweissae

Taxon classificationAnimaliaLepidopteraCrambidae

Léger & Nuss sp. nov.

2AA3B197-7CA0-5FB9-9BC2-E552096FC1F5

http://zoobank.org/D8FBB3AC-2B4B-436E-B939-70FB9B5E9336

Figs 14 , 57 , 95

##### Material examined.

***Holotype***: ♂, with labels: “M[oun]t. Goliath [Mount Yamin], | 5000 f[ee]t., Centr[al]. Dutch N[ew]. Guinea, | ca. 139° long, | February 1911, | (A. S. Meek).”; "NHMUK 010923397” [barcode appended]; “TL | 710 ♂”. Deposited in NHMUK.

***Paratypes***: 1 ♂, 1 ♀. Indonesia: 1 ♂ (NHMUK010923399), 1 ♀ (NHMUK010923351, genitalia on slide TL 709 ♀), same data as holotype (NHMUK).

##### Other specimens examined.

1 ♂. Papua New Guinea: 1 ♂, (DNA voucher MTD LEP3157 & genitalia on slide TL437 ♂) Morobe Province, Mount Kaindi, 2350 m, 11.xii.1976, leg. G. F. Hevel & R. E. Dietz (USNM).

##### Diagnosis.

This species is recognisable by the brown forewings broadly suffused with copper-coloured scales giving them a shiny appearance. Median cubital patch is white, discoidal stigma and postmedian patch are yellow, the latter marked with white toward dorsum and at costa. In male genitalia, uncus is elongated, with a truncate apex, and gnathos projection is ca. half the length of uncus, with truncate apex. In female genitalia, antrum is barely sclerotised, ductus bursae is long, slender and straight and corpus bursae is large, globular, with a small slightly curved thorn.

##### Similar species.

No similar species known.

##### Description.

***Head*.** Antennae dorsally bronze. Proboscis pale yellow. Maxillary palpi brown, inner side pale brown. Labial palpi brown, ventrally pale yellow, inner side pale brown.

***Thorax*** (Fig. 14). Collar pale yellow. Forewing length 11 mm (♂), 12 mm (♀); forewing ground colour brown; broad basal yellow band stretching from subdorsum to costa, with crossing veins copper-coloured, edged with copper; costal field copper; median discoidal stigma yellow with copper edges, costally filled with copper, forming together with cubital trapezoid white patch a canine tooth shape; postmedian patch broad, yellow, basally encroached with copper, crossing veins copper-coloured, with white blotch at costa; postmedian line broad, marked on costal half; broad copper postmedian fascia with crossing veins brown; subterminal field marked with a mix of yellow and copper scales; fringe brown, with white dots. Hindwing white to pale brown. Forelegs brown. Midlegs with femur brown; tibia pale yellow, apically brown; tarsi pale yellow to pale brown. Hindlegs with femur brown; tibia pale yellow, speckled with brown; tarsi pale brown.

***Abdomen*.** Male sternum A8 posterior margin bilobed.

***Male genitalia*** (*N* = 1) (Fig. 57). Uncus long and slender, with straight lateral margin, apex blunt. Gnathos projection ca. half the length of uncus, with blunt apex. Valva ventral margin nearly straight, in basal half slightly concave, dorsal margin conspicuously rounded, apex pointed. Juxta with base rounded, notched on its middle, medially narrowed, apex rounded, faintly notched on its middle. Saccus triangular. Phallus with truncate cornutus.

***Female genitalia*** (*N* = 1) (Fig. 95). Anterior apophyses conspicuously widened at posterior 1/3, with tip pointing dorsad. Antrum sclerotisation weak, ca. as long as broad. Ductus bursae of medium length, straight, slender. Corpus bursae large, globular, posterior half reticulated, anterior half membranous, with weak sclerotisation between corpus opening and thorn. Thorn long, slightly curved, with small dents pointing toward thorn base, basally with small outwardly projected extension.

##### Distribution.

Known from Mount Yamin (Indonesia: Papua), and Mount Kaindi (Papua New Guinea: Morobe Province), at altitudes between 1,700 m and 2,350 m.

##### Etymology.

Dedicated to the late Marijo Weiss, a close friend of Théo Léger’s family.

##### Remarks.

The specimen from Mount Kaindi is very similar to those from Mount Yamin but its postmedian fascia of the forewing is yellow and copper instead of dark copper, and uncus and gnathos projection are slightly longer in male genitalia.

#### Hoploscopa titika

Taxon classificationAnimaliaLepidopteraCrambidae

Léger & Nuss sp. nov.

12CE3C67-3870-5363-A455-9EA11AF59BF6

http://zoobank.org/8B31F2F7-6FD3-4C8D-85E2-43D87F3EEF3E

Figs 15 , 58

##### Material examined.

***Holotype***: ♂, with labels: “Sumatra-Holzweg | 25 km SSW-Pematangsiantar | straße nach Prapat [road to Prapat], L[icht]F[ang] [light trap] | 13.ii.1996, leg. A. Kallies”; “coll[ection]. M. Nuss | Geschenk 2000 | Museum für Tier- | kunde Dresden”; “DNA voucher | Lepidoptera | MTD 2016 | [vertically written:] no. 3206”; “TL505 | ♂”. Deposited in MTD.

##### Diagnosis.

*Hoploscopa titika* sp. nov. is recognisable by its well-marked white quadrangular median cubital spot on the forewing. In male genitalia, the gnathos projection is reduced to a small ridge, and the dorsal margin of the valva is rounded, broadly sclerotised. Female genitalia not known.

##### Similar species.

No similar species known.

##### Description.

***Head*.** Antennae dorsally with pale yellow to bronze scales. Proboscis white. Maxillary palpi brown, inner side white to pale brown. Labial palpi brown, ventro-basally white.

***Thorax*** (Fig. 15). Collar white. Forewing length: 9 mm; forewing ground colour brown; basal patch extending from dorsum to subcosta, red, with small basal pale yellow spot; median discoidal stigma triangular, pale yellow, filled with red scales, with white trapezoid cubital patch abutting dorsally; postmedian patch roughly quadrangular, pale yellow, filled with red scales; subterminal line diffuse, white; subterminal field marked with red and pale yellow; fringe missing on specimen. Forelegs lost. Mid- and hindlegs white to pale yellow.

***Abdomen*.** Male sternum A8 posterior margin bilobed, with short, rounded lateral projections.

***Male genitalia*** (*N* = 1) (Fig. 58). Uncus long and slender, gently tapering toward apex, apex tongue-shaped. Gnathos projection limited to small ridge. Valva broad, ventral margin bent dorsad on distal 1/3, dorsal margin conspicuously convex, apex pointed. Juxta with base rounded, medially straight, apex blunt. Saccus small, pointing dorsad. Phallus apically with sclerotised spine.

***Female genitalia*.** not known.

##### Distribution.

Known from North Sumatra (Indonesia).

##### Etymology.

The species name *titika* comes from the Indonesian word “titik” meaning spot or dot, referring to the white median cubital spot of the forewing.

#### Hoploscopa pangrangoensis

Taxon classificationAnimaliaLepidopteraCrambidae

Léger & Nuss sp. nov.

8B7EF575-7EA1-57B3-833E-74BCCE540B31

http://zoobank.org/B24AA14E-0E31-4CF0-BCDC-85DB29385473

Figs 16 , 59 , 97

##### Material examined.

***Holotype***: ♀, with labels: “Indonesia, Java, M[oun]t. Pangrange | 30 km S[outh]E[ast] Bogor, 1625 m | primary forest 16–20.ii.1996 | 6.30S 107.10E leg. Siniaev & Afonin”; “Lepidoptera | date: i.2018 | MTD 7433 | [vertically written:] DNA-voucher”; “TL | 636 ♀”. Deposited in MFNB.

***Paratypes***: 4 ♂, 1 ♀. Indonesia: 2 ♂ (1 with genitalia on slide TL659 ♂), 1 ♀ (DNA voucher MTD7431, genitalia on slide TL627 ♀), same data as holotype; 2 ♂ (1 with genitalia on slide TL660 ♂), same data as holotype except 21–26.ii.1996 (MFNB).

##### Diagnosis.

The forewing markings of *H. pangrangoensis* sp. nov. are reduced to a small crescent-shaped median discoidal stigma; median cubital and dorsal patches are not marked, and postmedian patch is reduced to a blotch at costa. In male genitalia, the uncus is medially widened and the gnathos shows a finger-like projection ca. half the length of uncus. In female genitalia, the thin longitudinal sclerotised lines of the antrum are unique to this species.

##### Similar species.

*Hoploscopa parvimacula* sp. nov. (q.v.), *H. sumatrensis* sp. nov. *Hoploscopa sumatrensis* sp. nov. shares with *H. pangrangoensis* sp. nov. the crescent-shaped pale yellow median discoidal stigma on the forewing and the small median cubital patch. However, median discoidal stigma and postmedian patch are filled with reddish brown in *H. sumatrensis* sp. nov. In male genitalia, the gnathos projection reaches 4/5 of the uncus length in *H. sumatrensis* sp. nov. In female genitalia, antrum of *H. sumatrensis* sp. nov. is completely sclerotised.

##### Description.

***Head*.** Antennae dorsally striped with brown and bronze scales. Proboscis pale brown. Maxillary palpi brown, ventro-basally pale yellow. Labial palpi brown, base and inner side pale yellow.

***Thorax*** (Fig. 16). Collar pale yellow. Forewing length: 10–11 mm (♂ & ♀); forewing ground colour brown; basal dark brown dash distally pale yellow; small pale yellow spot at base of cell; basal and distal discoidal patches dark brown; median discoidal stigma crescent-shaped, white to pale yellow; median cubital pale yellow blotch in some specimens; postmedian pale yellow streak at costa, distally with dark brown streak; postmedian area speckled with pale yellow; subterminal line pale yellow, diffuse; fringes brown, with pale yellow dots. Hindwing pale brown. Forelegs brown, tarsi pale brown. Midlegs with femur brown; tibia brown speckled with pale yellow; tarsi pale yellow speckled with bronze. Hindlegs with femur brown; tibia pale yellow, speckled with pale brown; tarsi pale yellow.

***Abdomen*.** Male sternum A8 posterior margin notched, with short, rounded lateral projections.

***Male genitalia*** (*N* = 2) (Fig. 59). Uncus medially widened, narrowed on apical 1/4, apex blunt. Gnathos projection finger-shaped, ca. half the length of uncus. Valva ventral margin straight, gently bent dorsad on distal 1/3, dorsal margin conspicuously convex; apex slightly pointed. Juxta with base quadrangular, medially narrowing, apex roughly rounded, slightly notched. Saccus triangular, conspicuously pointing dorsad.

***Female genitalia*** (*N* = 2) (Fig. 97). Anterior apophyses with dorsal bump at posterior 1/3. Antrum elongated, membranous, with thin longitudinal sclerotised lines, anteriorly with minute sclerotised markings. Ductus bursae short, bent before corpus opening. Corpus bursae pear-shaped, reticulated, with sclerotisation between thorn and corpus opening and faintly marked sclerotised band medially. Thorn small, straight, with small dents pointing toward thorn apex.

##### Distribution.

Known from the slopes of Mount Pangrango (3019 m) on Java (Indonesia), at an altitude of 1,625 m.

##### Etymology.

Named after Mount Pangrango, a dormant stratovolcano on Java, where the specimens were collected.

#### Hoploscopa isarogensis

Taxon classificationAnimaliaLepidopteraCrambidae

Léger & Nuss sp. nov.

20D3495C-86E8-50EF-9D40-9A67971AF2CD

http://zoobank.org/C77DFE2E-3B02-4738-8F3E-E8139D571FEB

Figs 17 , 60 , 100

##### Material examined.

***Holotype***: ♀, with labels: “Philippines, South Luzon | M[oun]t Isarog | 13°40'N, 123°20'E, 530 m | submontane forest, at light | 22.iii.2000, leg. M. Nuss”; “DNA Barcode | BC MTD 01431”; “DNA voucher | Lepidoptera | MTD 2016 | [vertically written:] no. 3198”; “TL514 | ♀”. Deposited in MTD.

***Paratypes***: 2 ♂, 7 ♀. Philippines: 1 ♂ (DNA vouchers MTD LEP14 & MTD LEP3209, genitalia on slide TL507 ♂), 5 ♀ (1 with DNA voucher MTD LEP43 & MTD LEP3208, genitalia on slide TL528 ♀; 1 with DNA barcode BC MTD 01430; 1 with DNA voucher MTD LEP3199 & genitalia on slide TL520 ♀, 1 with DNA voucher MTD LEP3200 & genitalia on slide TL523 ♀), same data as holotype; 1 ♂, 1 ♀ (♂ with genitalia on slide TL762 ♂), same date and locality as holotype, leg. Mey & Ebert; 1 ♀ (DNA voucher MTD7422 & genitalia on slide TL621 ♀), Laguna, Pangil, 50 m, 11.iv.1997, leg. Mey & Speidel (MTD).

##### Other specimens examined.

2 ♂, 7 ♀. Philippines: 1 ♂ (DNA voucher MTD7420 & genitalia on slide TL626 ♂), 6 ♀ (4 with DNA voucher MTD7418, MTD8143, MTD8144, MTD8146, MTD8148 and genitalia on slide TL630♀, TL704 ♀, TL705 ♀, TL720 ♀, TL719 ♀ respectively, 2 with DNA voucher MTD8145 & MTD8147 and abdomens in microvials), Leyte, Lake Danao, 650 m, 14.-17.4.1997, leg. W. Mey & W. Speidel (MFNB); 1 ♂ (DNA voucher MTD7423 & genitalia on slide TL631 ♂), Mindoro, Mt Baco Pass, 1150 m, 14.i.1998 (Mey & Samarita); 1 ♀ (DNA voucher MTD7424 & genitalia voucher on slide TL619 ♀), Mindoro, Mt Halcon, 1300 m, 15–17.i.1998 (Mey & Samarita) (MFNB);

##### Diagnosis.

*Hoploscopa isarogensis* sp. nov. is a relatively small brown-winged species (forewing length = 7–9.5 mm) with red markings edged yellow. Median discoidal stigma, cubital and dorsal patches form together a disrupted band, and postmedian patch is red with a pale yellow blotch at costa. In male genitalia, the apex of the uncus is duck beak-shaped, with a small marked bump on its ventral side, and gnathos projection is ca. 1/3 of uncus length, with an indented apex. In female genitalia, the corpus bursae displays a clearly delimited sclerotisation between the thorn and the corpus opening, and bears a long, thick, curved thorn.

##### Similar species.

*Hoploscopa cynodonta* sp. nov. (q.v.), *H. ignitamaculae* sp. nov. (q.v.), *H. agtuuganonensis* sp. nov. The forewings of *H. agtuuganonensis* sp. nov. display a trapezoid median discoidal stigma, and median markings are only slightly disrupted, while they are well separated in *H. isarogensis* sp. nov. In male genitalia, gnathos projection reaches 2/3 of uncus length and has a truncate apex. In female genitalia, ductus bursae is long, broad, with one loop, while it is shorter, slender and straight in *H. isarogensis* sp. nov., and the small corpus bursae displays one plump thorn with a small sclerotisation at its base.

##### Description.

***Head*.** Antennae dorsally brown. Proboscis pale yellow. Maxillary palpi brown, base and inner side pale brown. Labial palpi brown, ventro-basally pale yellow.

***Thorax*** (Fig. 17). Collar white. Forewing length: 7–8 mm (♂), 8–9.5 mm (♀); forewing ground colour brown; broad basal patch pale yellow to yellow, crossed by longitudinal reddish brown fascia running up to postmedian area; costal field reddish brown; median discoidal stigma rhomboid to elliptic, pale yellow, filled with reddish brown; cubital and dorsal pale yellow patches forming with median discoidal stigma an oblique band disrupted at veins; postmedian patch roughly triangular, reddish brown, more or less speckled with pale yellow, with pale yellow blotch at costa; subterminal line pale yellow, conspicuously incurved inwards at CuA2; subterminal field marked with reddish brown; fringe brown, with pale yellow dots. Hindwing pale brown. Forelegs brown. Midlegs brown, inner side pale yellow. Hindlegs brown, tibia marked with pale yellow.

***Abdomen*.** Male sternum A8 posterior margin broadly indented, with short, rounded lateral projections.

***Male genitalia*** (*N* = 2) (Fig. 60). Uncus long, medially broadened, narrowed at apical 1/4, apex duck beak-shaped, medio-ventrally with a small bump projecting ventrad. Gnathos projection ca. 1/3 of the uncus length, with apex notched. Valva ventral margin nearly straight, gently bent dorsad on distal 1/4, dorsal margin slightly convex, apex truncated. Juxta broad, with base rounded, medially slightly narrowed, apex slightly incurved. Saccus quadrangular.

***Female genitalia*** (*N* = 6) (Fig. 100). Anterior apophyses with small dorsal bump at posterior 1/3. Antrum sclerotisation short, ca. as wide as long. Ductus bursae relatively short, nearly straight. Corpus bursae globular and reticulated but in some specimens with a rounded pouch-like extension, with well-marked sclerotisation between thorn and ductus bursae opening, medially with faintly sclerotised band. Thorn sabre-like, curved, with small dents pointing toward thorn base.

##### Distribution.

Known from the Luzon and Mindoro islands (Philippines), between sea level and 1,150 m.

##### DNA-barcoding.

Specimens from Luzon and Leyte show an K2P-distance of 3.4–3.9% and are recovered in two MOTUs.

##### Etymology.

Named after Mount Isarog (1966 m), a volcano on Luzon Island (Philippines) where the species is found.

##### Remarks.

Minor differences were observed in male and female genitalia of the specimens from Leyte. However, with only one male from Leyte on hand, we refrained from drawing further conclusions on its species status here.

#### Hoploscopa ypsilon

Taxon classificationAnimaliaLepidopteraCrambidae

Léger & Nuss sp. nov.

1D4EE61F-3145-5404-911A-D41C5BD384C6

http://zoobank.org/06694A15-8524-4AB3-AAC0-2874511AC4F3

Figs 18 , 61 , 98

##### Material examined.

***Holotype***: ♂, with labels: “Philippinen, Luzon | M[oun]t[ai]n. Prov[ince]., Barlig | 1650 m, 14–15.XI.97 | leg. Mey, Ebert, Nuß”; “DNA voucher | Lepidoptera | date: ix.2018 | MTD8138 | [vertically written:] DNA-voucher”; “TL699 | ♂”. Deposited in MFNB.

***Paratypes***: 9 ♂, 15 ♀. Philippines: 4 ♂, 10 ♀ (1 with DNA voucher MTD7429 & genitalia on slide TL623 ♀), same data as holotype; 3 ♂ (1 with genitalia on slide TL677 ♂), 5 ♀ (1 with DNA voucher MTD7427 & genitalia on slide TL620 ♀, 1 with DNA voucher MTD7428 & genitalia on slide TL637 ♀), Luzon, Mountain Province, Chatol, 2100 m, 16–18.xi.1997, leg. Mey, Ebert & Nuss; 1 ♂ (DNA voucher 7435 & genitalia on slide TL624 ♂), Luzon, Ifugao, Mount Polis, 2000 m, 13.xi.1997, leg. Mey, Ebert & Nuss; 1 ♂ (DNA voucher MTD8137), Luzon, Santa Fe, Bald Mountain, 1150 m, 11–13.xi.1997, leg. Mey, Ebert & Nuss (MFNB).

##### Diagnosis.

*Hoploscopa ypsilon* sp. nov. displays well-marked white coloured markings on the forewing. Median discoidal stigma and cubital patch are Y-shaped, postmedian patch is triangular and subterminal line is conspicuously incurved inwards at CuA2. In male genitalia, the gnathos forms a short, broadly rounded projection.

##### Similar species.

*Hoploscopa danaoensis* sp. nov. This species displays pale yellow markings on the forewing, the postmedian area is more or less broadly suffused with pale yellow, with subterminal line only slightly incurved inwards at CuA2. In male genitalia, *H. danaoensis* sp. nov. has a tongue-shaped projection ca. half the uncus length. Female genitalia are very similar to those of *H. danaoensis* sp. nov. and cannot be confidently separated.

##### Description.

***Head.*** Antennae dorsally with brown scales. Proboscis brown, speckled with pale yellow. Maxillary palpi brown, inner side and base pale yellow. Labial palpi brown, ventral base and tip pale yellow.

***Thorax*** (Fig. 18). Collar pale yellow. Forewing length: 9–10 mm (♂), 9.5–11 mm (♀); forewing ground colour brown; basal dark brown blotch with oblique white streak abutting at costa; median discoidal stigma V-shaped, white, edged with darker brown, together with white cubital streak forming a Y; postmedian patch triangular, white, invaded with light brown toward costa, edged with darker brown; subterminal line white, conspicuously incurved inwards at CuA2; fringes brown, with pale yellow dots. Hindwing pale brown, darker at apex. Forelegs brown; tarsi brown to pale brown. Midlegs with femur brown; tibia brown, speckled with pale yellow; tarsi pale yellow. Hindlegs with femur brown; tibia brown speckled with pale yellow, distally pale yellow; tarsi pale yellow.

***Abdomen*.** Male sternum A8 posterior margin bilobed.

***Male genitalia*** (*N* = 3) (Fig. 61). Uncus long, slightly narrowed on apical 1/4, apex duck beak-shaped. Gnathos projection short, with broadly rounded apex. Valva ventral margin gently bent dorsad on distal 1/3, dorsal margin conspicuously convex, apex slightly pointed. Juxta with base rounded, medially slightly narrowed, apex incurved. Saccus small, quadrangular.

***Female genitalia*** (*N* = 3) (Fig. 98). Anterior apophyses with dorsal bump at 1/3. Antrum sclerotisation short, ca. as wide as long. Ductus bursae short, bent before corpus opening. Corpus bursae pear-shaped, reticulated, with sclerotisation between thorn and corpus opening and faintly marked sclerotised band medially. Thorn small, straight, with small dents pointing toward thorn apex.

##### Distribution.

Known from the Ifugao, the Mountain and the Nueva Vizcaya Provinces on Luzon (Philippines), at altitudes between 1,150–2,100 m.

##### DNA barcoding.

The highest intraspecific divergence is 0.7%. The nearest neighbor is *H. danaoensis* sp. nov. (2.9–3.1%).

##### Etymology.

The species name *ypsilon*, for the Greek letter Y, refers to the shape of the median markings on the forewing.

#### Hoploscopa danaoensis

Taxon classificationAnimaliaLepidopteraCrambidae

Léger & Nuss sp. nov.

4AB0F571-3797-5765-8370-6D02616D7F47

http://zoobank.org/CE945458-F671-4EE7-8E5B-FA18C6CC58AC

Figs 19 , 62 , 99

##### Material examined.

***Holotype***: ♂, with labels: “Philipinen [sic], Leyte | Lake Danao, 650 m | 14.-17.4.1997 | leg. Mey & Speidel”; “DNA voucher | Lepidoptera | date: i.2018 | MTD7419 | [vertically written:] DNA-voucher”; “TL | 632 ♂”. Deposited in MFNB.

***Paratypes***: 6 ♂, 11 ♀. Philippines: 6 ♂ (2 with genitalia on slide TL721 ♂ & TL722 ♂), 11 ♀ (1 with DNA voucher MTD7421 & genitalia on slide TL618 ♀, 1 with DNA voucher MTD8142 & genitalia on slide TL703, 1 with DNA voucher MTD8141 & genitalia on slide TL702 ♀), same data as holotype (MFNB).

##### Other specimens examined.

2 ♂, 9 ♀. Philippines: 1 ♂ (genitalia on slide TL681 ♂), 1 ♀ (DNA voucher MTD7417 & genitalia on slide TL615 ♀), Mindanao, 1050 m, Mt Agtuuganon, 28.v.-7.vi.1996, leg. W. Mey; 1 ♂ (DNA voucher MTD8140 & genitalia on slide TL701 ♂), 8 ♀ (DNA voucher MTD8139 & genitalia on slide TL700 ♀), Negros, Patag, Lake Danao, 1400m, 21.5.96, leg. W. Mey (MFNB).

##### Diagnosis.

The forewings of *H. danaoensis* sp. nov. displays pale yellow median markings forming a Y. In male genitalia, the uncus displays a straight lateral margin and an obtuse apex, and the tongue-shaped gnathos projection reaches ca. half of the uncus length. The juxta is progressively narrowing toward the notched apex. Female genitalia are very similar to several other *Hoploscopa* species, e.g., *H. ypsilon* sp. nov.

##### Similar species.

*Hoploscopa brunnealis* (q.v.), *H. ypsilon* sp. nov. (q.v.), *H. metacrossa* (q.v.).

##### Description.

***Head*.** Antennae dorsally with brown scales. Proboscis pale brown, of a darker brown at base. Maxillary palpi brown, basally pale yellow, inner side pale brown. Labial palpi brown, ventro-basally pale yellow, inner side speckled with pale yellow.

**Figures 19–33.**
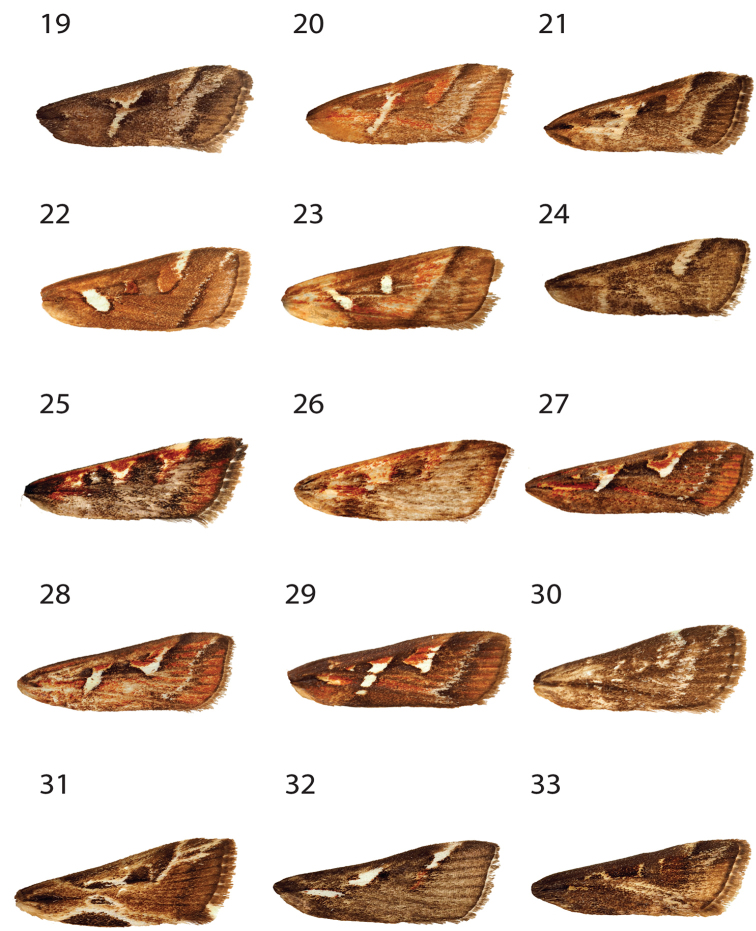
Forewing of *Hoploscopa* species **19***Hoploscopa danaoensis* sp. nov., paratype, ♂, Philippines, Leyte, Lake Danao, 650 m, 14–17.iv.1997 (Mey & Speidel) **20***Hoploscopa aurantiacalis* Snellen lectotype, ♀, NHMUK010923286, Indonesia, Java occ., Pengaleng, 4000 ft, 1893 (genitalia on slide Pyralidae Brit. Mus. Slide N° 20246) **21***Hoploscopa brunnealis* Snellen, lectotype, ♀, NHMUK010923292, Indonesia, Java occ., Pengaleng, 4000 ft, 1893 (genitalia on slide Pyralidae Brit. Mus. Slide N° 20247) **22***Hoploscopa ocellata* Hampson, holotype, ♀, NHMUK010923358, Indonesia, Moluccas, Batchian, iii.1892 (W. Doherty) (genitalia on slide Pyralidae Brit. Mus. Slide N° 20258) **23***Hoploscopa quadripuncta* Rotschild, holotype, ♂, NHMUK010923357, Indonesia, Papua, Utakwa River, 3000 ft, i.1913 (A.F.R. Wollaston) (genitalia on slide Pyralidae Brit. Mus. Slide N° 20257) **24***Hoploscopa semifascia* Hampson, ♀, NHMUK010923321, Papua New Guinea, Southern Highlands, Bosavi, 570 m, 6.i.1986 (D. Agassiz) (genitalia on slide TL740 ♀) **25***Hoploscopa subvariegata* Rothschild, ♂, Morobe Province, near Wau, Mt. Kaindi, 2360 m, 27–28.vii.1983 (S. E. & P. M. Miller) (genitalia on slide TL642 ♂) **26***Hoploscopa persimilis* Rothschild, lectotype, ♂, NHMUK010923328, Indonesia, Papua, Utawka River, 3000 ft., i.1913 (A.F.R. Wollaston) (genitalia on slide Pyralidae Brit. Mus. Slide N° 20255) **27***Hoploscopa astrapias* Meyrick, lectotype, ♀, NHMUK010923383, Fiji, Vunîdawa, 2.i.1932 (genitalia on slide Pyralidae Brit. Mus. Slide N° 20241) **28***Hoploscopa anamesa* Tams, ♀, NHMUK010923466, Vanuatu, Aneityum Island, Red Crest, 3 m NE of Anelgauhat, 1200 ft, vi.1955 (E. Cheesman) (genitalia on slide TL718 ♀) **29***Hoploscopa nauticorum* Tams, allotype, ♀, Samoan, Upolu Island, Malololelei, 2000 ft, 21.ii.1925 (P. A. Buxton & G. H. Hopkins) **30***Hoploscopa diffusa* Hampson, lectotype, ♂, NHMUK010923338, Papua New Guinea, Fergusson Island, x-xi.1894 (A. S. Meek) (genitalia on slide Pyralidae Brit. Mus. Slide N° 1014 ♂) **31***Hoploscopa triangulifera* Hampson, ♀, NHMUK010923453, Biagi, Mambare R., 1600 m, iii.1906 (A. S. Meek) (genitalia on slide TL712 ♀) **32***Hoploscopa anacantha* sp. nov., holotype, ♂, NHMUK010923444, Indonesia, North Sumatra, Dumoga-Bone N. P., Gunung Mogogonipa, summit, 1008 m, 18–20.x.1985 (genitalia on slide TL759 ♂) **33***Hoploscopa kelama* sp. nov., paratype, ♂, NHMUK010923342 , Indonesia, North Sulawesi, Dumoga-Bone National Park, Clarke Camp, lower montane forest, 1140 m, x.1985 (Royal Entomological Society of London, Project Wallace) (genitalia on slide TL759 ♂).

***Thorax*** (Fig. 19). Collar pale yellow. Forewing length: 8 mm (♂), 8–9 mm (♀); forewing ground colour brown; basal dark brown blotch distally pale yellow; median discoidal stigma V-shaped, white to pale yellow, with dark brown basal and distal edges; cubital patch elongated, white to pale yellow, together with median discoidal stigma forming a Y; postmedian patch roughly triangular, white to pale yellow, broadly invaded with brown, edged with darker brown; postmedian line marked on costal half; subterminal line thick, white to pale yellow, incurved inwards at CuA2, angled at M1; fringes brown, with diffuse pale yellow dots. Hindwing pale brown. Forelegs brown, tarsi pale brown. Midlegs with femur brown; tibia brown, speckled with pale yellow, distally pale yellow; tarsi pale yellow. Hindlegs with femur brown; tibia pale yellow, speckled with pale brown; tarsi pale brown.

***Abdomen*.** Male sternum A8 posterior margin straight.

***Male genitalia*** (*N* = 1) (Fig. 62). Uncus long with straight lateral margin, apex obtuse. Gnathos projection tongue-shaped, ca. half the uncus length. Valva ventral margin gently bent dorsad on distal 1/3, dorsal margin conspicuously convex, apex slightly pointed. Juxta with base quadrangular, tapering toward apex, apex notched. Saccus small, triangular, pointing dorsad.

***Female genitalia*** (*N* = 1) (Fig. 99). Anterior apophyses with dorsal bump at posterior 1/3. Antrum short, as long as broad. Ductus bursae short, straight, bent before corpus bursae. Corpus bursae broad, pear-shaped, reticulated, with elongated sclerotisation between thorn and corpus opening, medially with sclerotised band. Thorn straight, with small dents pointing toward thorn apex.

##### Distribution.

Known from Lake Danao (650 m) on Leyte island (Philippines).

##### DNA barcoding.

Morphologically similar specimens from Mindanao and Negros are recovered in two separate MOTUs and show a divergence of 3.9–4.2% with *H. danaoensis* sp. nov.

##### Etymology.

Named after Lake Danao on Leyte where the species is found.

##### Remarks.

Minor differences were observed in male genitalia of the specimens from Mindanao and Negros. However, we only had one male on hand for each series. In absence of further evidence, we refrained from describing further species here.

#### Hoploscopa aurantiacalis

Taxon classificationAnimaliaLepidopteraCrambidae

(Snellen, 1895)

89506280-27CF-5390-BC9C-0E5CD112DAA6

Figs 20 , 102

##### Material examined.

***Lectotype***: ♀, with labels: “Lecto- | type” [purple ringed]; “SYN- | TYPE” [blue ringed]; “F1893 | Java occ. | Pengaleng | 4000’ F[ee]t” [handwritten, label squared with silver]; “Syncrotaula | aurantiacalis | Snellen | det. M. Shaffer, 1967” [handwritten]; “Lectotype | Eudorina | aurantiacalis | Snellen | det. M. Nuß” [handwritten]; “99.113.”; “♀ | Pyralidae | Brit[ish].Mus[eum]. | Slide N°. | 20246”; “GU Nr 659 | prep. M. Nuß”; "NHMUK 010923286” [barcode appended]. Deposited in NHMUK.

##### Diagnosis.

The median discoidal stigma, cubital and dorsal patches of the forewings form an oblique thin white streak. In female genitalia, the membranous pouch anterad from the antrum and the leaf-shaped sclerotisation of the corpus bursae allows to recognise this species. Male genitalia are not known.

##### Similar species.

No similar species known.

##### Description.

***Head*.** Antennae dorsally brown. Proboscis pale yellow. Maxillary palpi brown, base pale yellow, inner side pale brown. Labial palpi brown, ventral base and inner side pale yellow.

***Thorax*** (Fig. 20). Collar white. Forewing length: 10 mm; forewing ground colour brown; basal longitudinal red fascia dorsally edged with pale yellow, running up to postmedian area; costal field reddish brown; median discoidal stigma v-shaped, white, forming oblique streak extending down to dorsal area; postmedian patch reddish brown speckled with white, with costal white blotch; postmedian line marked on costal half; postmedian suffusion white; subterminal line white; subterminal field broadly marked with reddish brown; margin brown, chequered with pale yellow. Hindwing pale brown. Fore- and midlegs brown to bronze. Hindlegs with femur bronze, tibia pale yellow speckled with bronze, tarsi bronze.

***Male genitalia*.** Not known.

***Female genitalia*** (*N* = 1) (Fig. 102). Anterior apophyses bent ventrad at 1/3, without bump. Antrum sclerotisation short. Ductus bursae of medium length, with a rounded pouch at base, one loop on its middle. Corpus bursae rounded, extending lateral into a broad pouch, posterior half reticulated, anterior half with erect papillae, with leaf-shaped sclerotisation at corpus opening. Thorn long, flattened, straight, thinly indented.

##### Distribution.

Recorded from West Java (Indonesia), at an altitude of 1,300 m.

##### Remarks.

Nuss (1998) transferred this species from *Eudorina* to *Hoploscopa* and designated the lectotype.

#### Hoploscopa brunnealis

Taxon classificationAnimaliaLepidopteraCrambidae

(Snellen, 1895)

D7BF27AB-FCA8-5B35-AACE-94F39DC814BF

Figs 21 , 103

Argyria xiphotoma Meyrick, 1938

##### Material examined.

***Lectotype***: ♀, with labels: “Lecto- | type” [round label, purple ringed]; “SYN- | TYPE” [round label, ringed blue]; “F1893 | Java occ[idental]. | Pengaleng | 4000’ F[ee]t” [handwritten, squared with silver]; “99.113.” [handwritten] | “Syncrotaula | brunnealis | Snellen [handwritten] | det. M. Shaffer, 1967”; “Lectotype | Eudorina | brunnealis | Snellen | det. M. Nuß” [handwritten]; “GU Nr. 660 | prep. M. Nuß”; “♀ | Pyralidae | Brit[ish].Mus[eum]. | Slide N°. | 20247”; "NHMUK 010923292” [barcode appended]. Deposited in NHMUK.

##### Diagnosis.

*Hoploscopa brunnealis* displays a forewing pattern very similar to that of *H. danaoensis* sp. nov., with a median pale yellow Y, a postmedian pale yellow patch invaded with brown or tawny and a pale yellow subterminal line. In female genitalia, the antrum sclerotisation is short, the ductus bursae is short, straight, and the corpus bursae is pear-shaped, with a small straight thorn. Male genitalia are not known.

##### Similar species.

*Hoploscopa danaoensis* sp. nov., *H. metacrossa* (q.v.), *H. parvimacula* sp. nov. (q.v.). Female genitalia of these species display a larger antrum sclerotisation.

##### Description.

***Head*.** Antennae dorsally with bronze scales. Proboscis pale brown to pale yellow. Maxillary palpi brown, base and inner side pale yellow. Labial palpi brown, ventral base and inner side pale yellow.

***Thorax*** (Fig. 21). Forewing length: 10 mm; ground colour brown; basal dash dark brown distally pale yellow; costal field tawny; median discoidal stigma and median cubital patch forming together a pale yellow-coloured Y, with basal and distal edges dark brown; postmedian patch tawny speckled with pale yellow, distally pale yellow, with costal pale yellow blotch; postmedian area suffused with pale yellow; subterminal line pale yellow; fringes brown, with pale yellow dots.

***Male genitalia*.** Not known.

***Female genitalia*** (*N* = 1) (Fig. 103). Anterior apophyses without dorsal bump at posterior 1/3, Antrum sclerotisation short. Ductus bursae of medium length, slender, more or less straight. Corpus bursae pear-shaped, with posterior half reticulated, anterior half membranous, with faintly marked sclerotisation between thorn and corpus opening, medially with faintly marked sclerotised band. Thorn small, straight, with small dents pointing toward thorn base.

##### Distribution.

Recorded from West Java (Indonesia) at an altitude of 1,300 m.

##### Remarks.

Nuss (1998) transferred this species from *Eudorina* to *Hoploscopa* and designated the lectotype. *Argyria xiphotoma* Meyrick, 1938 is synonymised with *Hoploscopa brunnealis* in the same paper.

#### Hoploscopa ocellata

Taxon classificationAnimaliaLepidopteraCrambidae

(Hampson, 1919)

C4EB7041-3726-532E-82C0-FAA2939DFBAB

Figs 22 , 104

##### Material examined.

***Holotype***: ♀, with labels: “Holo- | type” [round label, red ringed]; “Batchian [Bacan islands] | Mar[ch]. 1892 | W. Doherty”; “Pyrocrambia | ocellata | type ♀. H[a]mps[o]n.” [handwritten]; “♀ | Pyralidae | Brit[ish].Mus[eum]. | Slide N°. | 20258”; "NHMUK 010923358” [barcode appended]. Deposited in NHMUK.

##### Diagnosis.

The large oblique elliptic white patch at base of the forewing, the median trapezoid and postmedian semi-elliptic tawny patches are unique to *H. ocellata*. In female genitalia, the long, wrinkled and multicoiled ductus bursae, the ovoid corpus bursae bearing long acanthae, and the long glabrous thorn unequivocally segregates *H. ocellata* from other *Hoploscopa* species. Male unknown.

##### Similar species.

No similar species known.

##### Description.

***Head*.** Antennae dorsally brown. Proboscis pale yellow. Maxillary palpi brown, basally pale yellow. Labial palpi brown, ventro-basally pale yellow.

***Thorax*** (Fig. 22). Collar white. Forewing length: 10 mm; forewing ground colour brown; large elliptic white basal patch with dark brown edges; basal and distal discoidal patches of a darker brown; median discoidal stigma there between trapezoid- to square-shaped, tawny, basally thinly edged with white; postmedian patch semi-elliptic, tawny, distally with white costal streak; subterminal line more or less straight, disrupted at M1, white, distally dark brown on dorsal 2/3; subterminal field suffused with white; fringes brown with lighter spots. Hindwing pale brown. Fore- and hindlegs brown, with tibia and tarsi segments distally white. Midlegs lost.

***Male genitalia*.** Not known.

***Female genitalia*** (*N* = 1) (Fig. 104). Papillae anales thin, dorsally and ventrally not connected. Anterior apophyses bent ventrad at 1/3, without bump. Antrum membranous. Ductus bursae long, narrow, wrinkled, multi-coiled. Corpus bursae ovoid, with one half reticulate, the other half densely covered with long acanthae, medially with sclerotised band. Thorn long, slender, straight, without dents.

##### Distribution.

Recorded from Bacan island in the Moluccas (Indonesia).

##### Remarks.

Nuss (1998) transferred this species from *Eudorina* to *Hoploscopa*.

#### Hoploscopa quadripuncta

Taxon classificationAnimaliaLepidopteraCrambidae

(Rotschild, 1915)

2F4A5F48-28EE-56E9-9BCC-87B0C323428E

Figs 23 , 63 , 105

##### Material examined.

***Holotype***: ♂, with labels: “Holo- | type” [round label, red ringed]; “Utakwa [sic, Oetakwa] R[iver]., | Dutch N[ew]. Guin[ea]., | 3000 f[ee]t., Jan. 1913. | A.F.R. Wollaston.”; “Eudorina | quadripuncta | Type Rotsch[ild].”; “436”; “♂ | Pyralidae | Brit[ish].Mus[eum]. | Slide N°. | 20257”; "NHMUK 010923357” [barcode appended]. Deposited in NHMUK.

##### Other specimens examined.

3 ♂, 1 ♀. Indonesia: 1 ♂ (NHMUK010923460, DNA voucher MTD8249 & genitalia on slide TL734 ♂), Moluccas, Seram, Gunung Kobipoto, north slopes, 570 m (NHMUK010923460), lowland forest, viii-ix.1987, leg. J. D. Holloway, D. T. Jones et al.; 1 ♂ (NHMUK010923459, DNA voucher MTD8248 & genitalia on slide TL733 ♂), same data except 900 m. Papua New Guinea: 1 ♂, 1 ♀, Hydrographers Mountains, 830 m, i.1918, Rothschild bequest (NHMUK).

##### Diagnosis.

The white-coloured basal patch and median discoidal stigma as well as the oblique postmedian line allows separation of *H. quadripuncta* from its congeneric species. In male genitalia, uncus apex is cuneate and gnathos projection is ogive-shaped. In female genitalia, the thorn of the corpus bursae is long, slender, curved, and shows a well-marked rounded sclerotisation at its base.

##### Similar species.

No similar species known.

##### Description.

***Head*.** Antennae dorsally bronze. Proboscis white. Maxillary palpi brown, base and inner side pale brown. Labial palpi brown, ventral base and inner side white.

***Thorax*** (Fig. 23). Collar pale yellow. Forewing length: 8.5–10 mm (♂), 10 mm (♀); forewing ground colour brown; basal quadrangular oblique white patch, basally and distally edged with dark brown, with tawny to reddish brown fascia along CuA2 spreading distally into postmedian area up to costa; median white rounded patch edged with dark brown; postmedian line oblique, straight, thick; fringes brown. Hindwing pale brown. Forelegs brown. Midlegs with femur brown, tibia and tarsi pale yellow. Hindlegs pale brown.

***Abdomen*.** Male sternum A8 posterior margin notched.

***Male genitalia*** (*N* = 3) (Fig. 63). Uncus long and slender, narrowed at apical 1/4, apex cuneate. Gnathos projection ogival, ca. 1/3 of uncus length. Valva slender, ventral margin straight on basal 2/3, bent dorsad on apical 1/3, dorsal margin convex, apex pointed. Juxta with base rounded, medially narrowed, apex broad, weakly sclerotised. Saccus broad, triangular, pointing dorsad. Phallus with flat spatula-shaped cornutus, apically with narrow bump.

***Female genitalia*** (Fig. 105). Anterior apophyses with tip pointing upward at posterior 1/3. Antrum with a narrow weakly sclerotised ring. Ductus bursae of medium length, straight. Corpus bursae ovoid, posterior half reticulated, anterior half membranous, sclerotised between thorn and corpus opening, medially with faintly sclerotised band. Thorn long, curved, with small dents pointing toward thorn base on posterior edge, basally with conspicuous outwardly directed plump extension.

##### Distribution.

Recorded from the Oetakwa River (Papua: Indonesia) in New Guinea, also known from the Oro Province (Papua New Guinea) and the Moluccas (Indonesia), at altitudes between 500 m and 1,000 m.

##### Remarks.

Nuss (1998) transferred this species from *Eudorina* to *Hoploscopa*. Other specimens examined from the Moluccas and Papua New Guinea display the same pattern as the holotype from Western New Guinea, however a small difference in the shape of the gnathos projection is observed: it is pointed in the holotype, while it is rounded in the other specimens. Unfortunately, no molecular investigations were done on the holotype.

#### Hoploscopa semifascia

Taxon classificationAnimaliaLepidopteraCrambidae

(Hampson, 1919)

AC17B452-1D0C-51C3-9F30-599CE0ED23A4

Figs 24 , 106

##### Material examined.

***Holotype***: ♀, with labels: “Holo- | type” [round label, red ringed]; “Fak-Fak | Dutch NewGuinea | Dec’[19]07 | 1700 f[ee]t | (Pratt)”; “1913-216.”; “Eudorina | semifascia | type ♀ H[a]mps[o]n” [handwritten]; “♀ | Pyralidae | Brit[ish].Mus[eum]. | Slide N°. | 20253”; "NHMUK 010923320” [barcode appended]. Deposited in NHMUK.

##### Other specimens examined.

2♀; Papua New Guinea: 1♀ (NHMUK010923321, DNA voucher MTD8242, genitalia on slide TL740 ♀), Southern Highlands, Bosavi, 570 m, 6.i.1986, leg. D. Agassiz (NHMUK); 1 ♀ (DNA voucher MTD LEP3158, genitalia on slide TL438 ♀), Morobe Province, Wau, Wau Ecology Institute, 24–26.viii.1983, 1360 m, leg. S. E. & P. M. Miller (USNM).

##### Diagnosis.

The forewings of *H. semifascia* display an antemedian dark brown fascia edged with pale yellow and a marked postmedian pale yellow patch. Median markings are completely lacking. In female genitalia, the thorn is long, thin, curved in distal half, with a small, well-marked sclerotisation at its base. Male unknown.

##### Similar species.

No similar species known.

##### Description.

***Head*.** Antennae dorsally striped with bronze and brown scales. Proboscis pale yellow. Maxillary palpi brown, base and inner side pale yellow. Labial palpi brown, base pale yellow, inner side pale yellow to pale brown.

***Thorax*** (Fig. 24). Collar white. Forewing length: 7 mm; forewing ground colour brown; antemedian broad transversal dark brown fascia, basal edge pale yellow, incurved inwardly on costal half, distal edge oblique, pale yellow; postmedian elongated pale yellow patch with dark brown edges; fringes brown, with pale yellow spots. Hindwing pale brown. Forelegs brown. Midlegs brown; tarsi pale brown. Hindlegs with femur brown; tibia pale yellow, speckled with brown; tarsi pale brown.

***Male genitalia*.** Not known.

***Female genitalia*** (*N* = 3) (Fig. 106). Anterior apophyses widened at posterior 1/3, with dorsal tip. Antrum sclerotisation short, as long as broad. Ductus bursae of medium length, slightly curved twice. Corpus bursae reticulated, with small rounded sclerotisation at thorn base. Thorn long and slender, curved, glabrous, basally with small outwardly projected extension.

##### Distribution.

Recorded from Fak-Fak (Papua, Indonesia), also known from the Southern Highlands and the Morobe Province (Papua New Guinea) at altitudes between 550 m and 1,400 m.

##### DNA barcoding.

The K2P-distance between specimens MTD LEP3158 from the Morobe Province and MTD8242 from the Southern Highlands (Papua New Guinea) is 0.5%.

##### Remarks.

Nuss (1998) transferred this species from *Eudorina* to *Hoploscopa*. Other specimens examined from Papua New Guinea display the same pattern and identical genitalia as the holotype from Western New Guinea.

#### Hoploscopa subvariegata

Taxon classificationAnimaliaLepidopteraCrambidae

(Rotschild, 1915)

FD0C89BF-34F7-5C52-B479-777CB7617839

Figs 25 , 64 , 109

##### Material examined.

***Holotype***: ♀, with labels: “Holo- | type” [round label, red ringed]; “Angabunga R[iver]., | affl[uent]. of St. Joseph | R., Brit[ish].N[ew].Guinea, | 6000 f[ee]t, upwards. | Nov.[19]04.-Febr.[19]05. | (A. S. Meek).”; “Eudorina | subvariegata | Type Rotsch[ild].” [handwritten]; “♀ | Pyralidae | Brit[ish].Mus[eum]. | Slide N°. | 20254”; "NHMUK 010923326” [barcode appended]. Deposited in NHMUK.

##### Other specimens examined.

14 ♂, 4 ♀. Papua New Guinea: 10 ♂ (2 with genitalia on slide TL478 ♂ & TL642 ♂), 1 ♀ (genitalia on slide TL521 ♀), Morobe Province, near Wau, Mt. Kaindi, 2360 meters, 27–28 July 1983, leg. S. E. & P. M. Miller; 1 ♂ (genitalia on slide TL534 ♂), same data except 29–30.viii.1983; 1 ♂, 1♀, same locality, 11.xii.1976, leg. G. F. Hevel & R. E. Dietz; 2 ♂ (1 with DNA voucher 3156, genitalia on slide TL436 ♂), 1 ♀, same locality, 3.x.1992, leg. V. O. Becker); 1 ♂, 1 ♀ (genitalia on slide TL471 ♀), Morobe Province, Biaro Road, 2000 m, 25.ix.1992, leg. V. O. Becker (USNM).

##### Diagnosis.

*Hoploscopa subvariegata* displays four roughly triangular yellow spots filled with reddish brown on forewing costa. In male genitalia, uncus is long and slender with truncate apex, gnathos projection is tongue-shaped, reaching 1/3 of uncus length, and dorsal margin of the valva is conspicuously protruded dorsad. In female genitalia, ductus bursae is long and straight, corpus bursae is large, globular and bears a small, slightly curved thorn with leaf-shaped sclerotisation at its base.

##### Similar species.

*Hoploscopa persimilis*. The latter species lacks the subterminal triangular yellow blotch at forewing costa. In the male genitalia of *H. persimilis*, uncus apex is duck-shaped, gnathos projection forms a small triangular tip, and valva dorsal margin is not protruded. Female of *H. persimilis* is not known.

##### Description.

***Head*.** Antennae dorsally with bronze scales. Proboscis pale yellow, basally brown. Maxillary palpi brown. Labial palpi brown, ventro-basally pale yellow.

***Thorax*** (Fig. 25). Collar white. Forewing length: 10 mm (♂), 9.5–12 mm (♀); forewing ground colour brown to dark brown; basal area reddish brown, basally with small yellow dash, at costa with V-shaped yellow patch filled with reddish brown; antemedian cubital snow-white dot; costal field reddish brown; median discoidal stigma V-shaped, white, yellow toward costa, filled with reddish brown; postmedian patch reddish brown, with white to yellow basal edge and pale yellow to yellow costal blotch; subterminal costal spot pale yellow to yellow, encroached with reddish brown; subterminal field reddish brown; margin brown, with white dots. Hindwing dirty white, bronze toward margin. Forelegs femur and tibia brown; tarsi pale yellow. Midlegs with femur brown; tibia pale yellow, dorso-distally brown; tarsi pale yellow. Hindleg femur brown; tibia pale yellow, dorso-distally brown; tarsi pale yellow to pale brown.

***Abdomen*.** Male sternum A8 posterior margin bilobed.

***Male genitalia*** (*N* = 4) (Fig. 64). Uncus long and slender, gently tapering toward apex, apex roughly truncate. Gnathos projection tongue-shaped, ca. 1/3 of uncus length. Valva ventral margin nearly straight, dorsal margin strongly protruded dorsad, apex truncate. Juxta with broad rounded base, medially wide, apex broadly incurved, weakly sclerotised. Saccus broad, triangular, pointing dorsad. Phallus with elongated, flat, spatula-shaped cornutus apically with narrow bump.

***Female genitalia*** (*N* = 2) (Fig. 109). Anterior apophyses with small dorsal tip at posterior 1/3. Antrum sclerotisation short, as long as broad. Ductus bursae of medium length, slender, straight. Corpus bursae large, globular, posterior half reticulated, anterior half membranous, with sclerotisation between thorn and corpus opening, medially with faintly marked sclerotised band. Thorn slightly curved, with small dents pointing toward thorn base.

##### Distribution.

Recorded from the Angabunga River in the Central Province (Papua New Guinea), also known from Mount Kaindi, Morobe Province, at altitudes between 2,000 m and 2,360 m.

##### Remarks.

Nuss (1998) transferred this species from *Eudorina* to *Hoploscopa*. Other specimens examined from Papua New Guinea display the same pattern and identical genitalia as the holotype from Western New Guinea.

#### Hoploscopa persimilis

Taxon classificationAnimaliaLepidopteraCrambidae

(Rotschild, 1915)

EA594C73-2BA9-5EC5-A605-0147257A1B99

Figs 26 , 65

##### Material examined.

***Lectotype***: ♂, with labels: “Lecto- | type” [round label, purple ringed]; “Utakwa [sic, Oetakwa] R[iver]., | Dutch N[ew]. Guin[ea]., | 3000f[ee]t., Jan[uary]. 1913. | A.F.R. Wollaston.”; “439”; “Eudorina | persimilis | Type Rotsch[ild].”; “Lectotype | Eudorina | persimilis | Rothschild | det. Nuß” [handwritten]; “♂ | Pyralidae | Brit[ish].Mus[eum]. | Slide N°. | 20255”; "NHMUK 010923328” [barcode appended].

##### Diagnosis

. *Hoploscopa persimilis* displays three nearly triangular yellow spots filled with reddish brown on the costa of the forewing. In male genitalia, uncus is long, slender, with duck-shaped apex, gnathos is projected into a small, triangular pointed tip, and valva dorsal margin is conspicuously convex. Female genitalia not known.

##### Similar species.

*Hoploscopa subvariegata* (q.v.).

##### Description.

***Head*.** Not examined.

***Thorax*** (Fig. 26). Collar pale yellow. Forewing length: 9 mm; forewing ground colour brown; basal patch elongated, reddish brown, basally crossed by transverse pale yellow streak; costal field reddish brown; median discoidal stigma trapezoid, pale yellow, with basal and distal edges reddish brown; postmedian patch triangular, pale yellow and reddish brown, distally with costal pale yellow blotch; subterminal field tawny; fringes brown with pale yellow spots. Hindwing pale yellow. Legs missing or badly preserved.

***Male genitalia*** (*N* = 1) (Fig. 65). Uncus long, slender, slightly narrowed on apical 1/4, apex duck beak-shaped. Gnathos projection triangular. Valva ventral margin nearly straight, dorsal margin conspicuously convex, apex roughly rounded. Juxta not clearly visible on the slide. Saccus broad, triangular, pointing dorsad. Phallus apically with sclerotised spine.

***Female genitalia*.** Not known.

##### Distribution.

Described from the Oetakwa River on New Guinea (Indonesia: Papua), at an altitude of 1,000 m.

##### Remarks.

Nuss (1998) transferred this species from *Eudorina* to *Hoploscopa* and designated the lectotype. The worn-out specimens hampered accurate description.

#### Hoploscopa diffusa

Taxon classificationAnimaliaLepidopteraCrambidae

(Hampson, 1919)

6A4F57AA-3531-5A39-8B15-31D1D94F03A7

Figs 30 , 66

##### Material examined.

***Lectotype***: ♂, with labels: “Lecto- | type” [round label, purple ringed]; “Syn- | type” [round label, blue ringed]; “Fergusson I., | X, XI. [18]94 | (A. S. Meek).”; “97–80”; “Pyrocrambia | diffusa. | type ♂. H[a]mpson.” [handwritten]; “Pyralidae | Brit[ish].Mus[eum]. | Slide N°. | 1014 ♂”; “Lectotype | Eudorina | diffusa | Hampson | det. Nuß” [handwritten]; "NHMUK 010923338 [barcode appended]”. Deposited in NHMUK.

##### Other specimens examined.

2 ♂. Papua New Guinea: 2 ♂ (1 with NHMUK010923439 & genitalia on slide TL715 ♂, 1 with NHMUK010923440 & genitalia on slide TL716 ♂), same data as holotype (NHMUK).

##### Diagnosis.

*Hoploscopa diffusa* displays a brown forewing with postmedian patch, part of postmedian area and subterminal line white coloured, and fringes marked with white dots. In male genitalia, gnathos projection is triangular with rounded tip, valva dorsal margin is conspicuously protruded and juxta is tongue-shaped. Female unknown.

##### Similar species.

*Hoploscopa niveofascia* sp. nov. (q.v.).

##### Description.

***Head*.** Antennae dorsally with brown scales. Proboscis white. Maxillary palpi brown, pale brown at base. Labial palpi brown, ventro-basally white.

***Thorax*** (Fig. 30). Collar white. Forewing length: 9 mm; forewing ground colour dark brown; small white dots scattered in basal and median area; postmedian patch white, filled with brown, with white costal blotch; postmedian line marked on costal half; postmedian area medially suffused with white scales; subterminal line white, shifted distally at M1; margin with white and black spots; fringe brown, with white dots; hindwing pale brown. Fore- and midlegs brown; tibia and tarsi segments distally white. Hindlegs similar, of a lighter brown.

***Abdomen*.** Male sternum A8 posterior margin notched, with short, rounded lateral projections.

***Male genitalia*** (*N* = 3) (Fig. 66). Uncus long, narrowed on apical 1/4, apex duck beak-shaped. Gnathos projection triangular, with rounded apex. Valva broad, ventral margin conspicuously bent dorsad on apical 1/4, dorsal margin strongly protruding dorsad, apex pointed. Juxta with base rounded, of constant width, apex rounded. Saccus small, pointing dorsad. No cornutus visible on the slide.

***Female genitalia*.** Not known.

##### Distribution.

Recorded from Fergusson Island (Papua New Guinea).

##### Remarks.

Nuss (1998) transferred this species from *Eudorina* to *Hoploscopa* and designated the lectotype.

#### Hoploscopa triangulifera

Taxon classificationAnimaliaLepidopteraCrambidae

(Hampson, 1919)

AAA9CA38-77DC-5A21-A1A3-6C62296853E4

Figs 31 , 67 , 110

##### Material examined.

***Holotype***: ♂, with labels: “Holo- | type” [round label, red ringed]; “Fergusson I[sland]. | [word crossed] xii.[18]95, | (A, S, Meek).”; “.97.204.”; “Eudorina | triangulifera | type ♂. H[a]mps[o]n”[handwritten]; “Body re-affixed | 6.xii.[19]40 R.S.C.” [handwritten]; “♀ | Pyralidae | Brit[ish].Mus[eum]. | Slide N°. | 20256”; "NHMUK010923353”[barcode appended]. Deposited in NHMUK.

##### Other specimens examined.

2♂, 2♀. Papua New Guinea: 1 ♂ (NHMUK010923452, genitalia on slide TL750 ♂), same locality as holotype, xi-xii.1894; 1 ♂ (genitalia on slide TL711 ♂, NHMUK010923454), 1 ♀ (genitalia on slide TL712 ♀, NHMUK010923453), Biagi, Mambare R., 1600 m, iii.1906, leg. A. S. Meek; 1 ♀ (NHMUK010923455), Dampier Island [Karkar], ii-iii.1914 (NHMUK).

##### Diagnosis.

The large median dorsal patch with costal margin rounded on the forewing is unique to *H. triangulifera*. In male genitalia, the gnathos shows no distal projection, and the saccus is broad, quadrangular. In female genitalia, ductus bursae is short and slender, corpus bursae is large, globular, and bears a long curved thorn.

##### Similar species.

No similar species known.

##### Description.

***Head*.** Antennae dorsally pale yellow. Proboscis brown. Maxillary palpi brown, inner side pale brown. Labial palpi brown, ventral base pale yellow, inner side pale brown.

***Thorax*** (Fig. 31). Thorax brown, dorsally pale yellow. Collar pale yellow. Forewing length: 7–8 mm (♂), 8–9 mm (♀); forewing ground colour brown; basal and distal discoidal stigmata dark brown; median discoidal stigma forming roughly defined white X; median cubital patch elliptic, dark brown with white edges; large median dark brown patch on dorsum with rounded pale yellow costal margin; postmedian patch triangular, dark brown; postmedian line pale yellow, marked on costal half; pale yellow and brown postmedian fascia on costal half in some specimens, edged distally by white subterminal line; fringes brown with white spots. Hindwing pale brown. Forelegs bronze, tarsi speckled with pale yellow. Midlegs with femur bronze; tibia brown, distally pale yellow; tarsi bronze. Hindlegs with femur bronze; tibia brown, basally and distally pale yellow; tarsi bronze.

***Male genitalia*** (*N* = 2) (Fig. 67). Uncus broad, narrowing on distal half, apex truncate. Gnathos projection limited to small ridge. Valva ventral margin nearly straight, dorsal margin conspicuously convex, apex pointed. Juxta with broad rounded base, medially wide, apex broadly indented, weakly sclerotised. Saccus broad, quadrangular.

***Female genitalia*** (*N* = 2) (Fig. 110). Anterior apophyses without dorsal bump at posterior 1/3. Antrum sclerotisation ca. twice as long as broad. Ductus bursae short, straight, slender. Corpus bursae globular, reticulated, with sclerotisation between thorn and corpus opening, and faintly marked sclerotised band medially. Thorn long, incurved, with small dents pointing toward thorn base on inner side, glabrous on outer side.

##### Distribution.

Known from the Madang, the Northern and the Milne Bay Provinces (Papua New Guinea) at altitudes of ca. 1,600 m.

##### Remarks.

Nuss (1998) transferred this species from *Eudorina* to *Hoploscopa*. The handwritten label from Hampson stipulates that the type is a male, while the abdomen dissected shows it is a female. The abdomen was re-affixed to the specimen, suggesting that either the sex wasn’t identified properly by Hampson or that the wrong abdomen was reaffixed to the specimen.

#### Hoploscopa astrapias

Taxon classificationAnimaliaLepidopteraCrambidae

Meyrick, 1886

C661CE39-025B-5998-A3CD-CEE68E6F6C72

Figs 27 , 68 , 107

##### Material examined.

***Lectotype***: ♀, with labels: “Lecto- | type” [round label, purple ringed]; “Vunîdawa | Fiji | HP. 2. 1. [19]32” [handwritten]; “HOPLOSCOPA Meyr.” [handwritten]; “astrapias Meyr.” [handwritten]; “Hoploscopa | astrapias | 1/1 Meyrick [handwritten] | E. Meyrick det. | in Meyrick Coll.”; “Lectotype | Hoploscopa | astrapias | Meyrick | det. M. Nuß” [handwritten]; “Meyrick Coll. | B. M. 1938-290.”; “♀ | Pyralidae | Brit[ish].Mus[eum]. | Slide N°. | 20241”; “GU 654 | Hoploscopa | astrapias | Matthias Nuß”; "NHMUK 010923383” [barcode appended]. Deposited in NHMUK.

##### Other specimens examined.

1 ♂, 1 ♀. Fiji: 1 ♂ (DNA voucher MTD8251, genitalia on slide TL724 ♂), 1 ♀ (DNA voucher MTD8250, genitalia on slide TL723 ♀), Viti Levu, Nandarivatu, 820 m, 14.9.1955, leg. H. W. Simmonds (NHMUK).

##### Diagnosis.

The forewings of *H. astrapias* display a median cubital triangular white patch and a bean-shaped postmedian patch red with yellow edges, distally with thick white streak. In male genitalia, the uncus is slender, elongated, the gnathos is reduced to a ring without posterior projection and the valva is slender, with an evenly rounded apex. In female genitalia, the corpus bursae is large, spherical, and bears a large, straight, glabrous thorn.

##### Similar species.

*Hoploscopa anamesa*, *H. nauticorum* but the latter can be separated from *H. astrapias* and *H. anamesa* based on the forewing: median markings form an elongated white streak running down to dorsal area, disrupted at veins (forming roughly triangular white patch not extending beyond CuA2 in the two other species), and the postmedian patch is quadrangular. In male genitalia, the bristles at uncus apex observed in *H. nauticorum* are absent or reduced in *H. astrapias* and *H. anamesa*, and the valva dorsal margin is strongly produced dorsad on basal half, with a more pointed apex in *H. nauticorum* (dorsal margin slightly convex in two other species, apex evenly rounded). Forewing and male genitalia of *H. astrapias* and *H. anamesa* do not provide unambiguous diagnostic characters to separate them. Median cubital patch of *H. anamesa* is slightly thicker than that of *H. astrapias* in specimens examined. Female genitalia allow clear segregation of these two species: antrum is membranous in *H. anamesa*, while it is lightly sclerotised, twice as long as broad in *H. astrapias*, and ductus bursae is long, gently coiled twice in *H. anamesa*, while it is short and broadly curved in *H. astrapias*.

##### Description.

***Head*.** Antennae dorsally with brown scales. Proboscis white to pale yellow. Maxillary palpi brown, base and inner side pale brown. Labial palpi brown, ventro-basally pale yellow to white.

***Thorax*** (Fig. 27). Collar white. Forewing length: 10–11 mm (♂ & ♀); forewing ground colour brown; basal yellow streak along 1A+2A, abutted with cubital reddish brown fascia running up to median area, disrupted by median cubital patch; costal field reddish brown; median discoidal stigma trapezoid, reddish brown, edged basally and distally with yellow, median cubital patch triangular, snow white, together with median discoidal stigma forming a canine tooth shape; post-median patch bean-shaped, reddish brown with yellow edges, with thick snow white streak abutting dorsally; postmedian area suffused with reddish brown; subterminal line white, not reaching dorsum; subterminal field broadly marked with reddish brown; fringes brown. Hindwing pale yellow, bronze toward distal margin. Forelegs brown. Midlegs brown to bronze; tibia medially white. Hindlegs brown to bronze, tibia base dorsally pale yellow.

***Male genitalia*** (*N* = 1) (Fig. 68). Uncus long, slender, with straight lateral margin, apex narrow, tongue-shaped, dorsally with sclerotised bristles. Gnathos projection limited to small ridge. Valva slender, ventral margin nearly straight, dorsal margin slightly convex, apex rounded. Juxta broad, with base rounded, apex weakly sclerotised, slightly incurved. Saccus small, pointing dorsad. Phallus with large flat spatula-shaped cornutus.

***Female genitalia*** (*N* = 1) (Fig. 107). Anterior apophyses with dorsal bump at posterior 1/3. Antrum lightly sclerotised, twice as long as broad. Ductus bursae of medium length, broadly curved. Corpus bursae globular, with posterior half reticulated, anterior half membranous, with weak sclerotisation between thorn and corpus opening. Thorn long and slender, straight, glabrous.

##### Distribution.

Known from the island of Viti Levu (Fiji), at altitudes between 0 and 800 m.

##### DNA barcoding.

*Hoploscopa astrapias* shows a divergence of 3.9% with *H. anamesa* and 3.3–4.7% with *H. nauticorum*.

##### Phylogenetic relationships.

*Hoploscopa astrapias*, *H. anamesa*, and *H. nauticorum* are recovered together in the ML analysis of the COI barcode (BS = 61). This topology is congruent with the morphology of these three species: the forewings display a snow white transversal median line and postmedian streak; in male genitalia, the uncus is elongated, slender, the gnathos is reduced to a sclerotised band without posterior projection, the phallus bears a large, flattened, spatula-shaped cornutus; in female genitalia, the corpus bursae is globular and bears a long and slender straight thorn. Within this clade, a close relationship between *H. astrapias* and *H. anamesa* is supported by both morphology and molecular data (BS = 78).

#### Hoploscopa anamesa

Taxon classificationAnimaliaLepidopteraCrambidae

Tams, 1935

24008D40-CA8A-5BD1-A327-D5B698A5FB20

Figs 28 , 69 , 108

##### Material examined.

***Lectotype***: ♀, with labels: “LECTO- | TYPE” [round label, purple ringed]; “SYN- | TYPE” [round label, blue ringed]; "New Hebrides: | Tanna. | ix.1930. | L.E.Cheesman. | B.M.1931-30.”; “Lectotype | Hoploscopa | astrapias | anamesa | Tams | det. Nuß” [handwritten]; "NHMUK 010923391” [barcode appended]. Deposited in NHMUK.

##### Other specimens examined.

2 ♂, 2 ♀. Vanuatu: 1 ♂ (NHMUK010923465), 1 ♀ (NHMUK010923466, genitalia on slide TL718 ♀), Aneityum Island, Red Crest, 3 [k]m NE of Anelgauhat, 1200ft, vi.1955, leg. E. Cheesman; 1 ♂ (NHMUK010923471, DNA voucher MTD8254, genitalia on slide TL727 ♂), 1 ♀ (NHMUK010923470, DNA voucher MTD8255, genitalia on slide TL728 ♀), Aneityum Island, Agathis Camp, 1150 ft, 21.vii.1971, leg. G. S. Robinson (NHMUK).

##### Diagnosis.

*Hoploscopa anamesa* (Fig. 28) display forewing and male genitalia similar to those of *H. astrapias*. Female genitalia differ from those of *H. astrapias* in following characters: the antrum is not sclerotised, and the ductus bursae is long and coiled. Corpus bursae and thorn similar to *H. astrapias*.

##### Similar species.

*Hoploscopa astrapias* and *H. nauticorum* (q.v.).

##### Description.

***Head*.** Antennae dorsally with brown scales. Proboscis white to pale yellow. Maxillary palpi brown, base and inner side pale brown. Labial palpi brown, ventro-basally pale yellow to white.

***Thorax*** (Fig. 28). Collar white. Forewing length: 10–11 mm (♂ & ♀); forewing ground colour brown; basal yellow streak along 1A+2A, abutted by cubital red fascia running up to median area, disrupted by median cubital patch; costal field reddish brown; median discoidal stigma trapezoid, red, edged basally and distally with yellow; median cubital patch thick, streak-like, snow white; post-median patch bean-shaped, red with yellow edges, dorsally abutted by thick snow white streak; postmedian area suffused with red; subterminal line white, not reaching dorsum; subterminal field broadly marked with red; fringes brown. Hindwing pale yellow, bronze toward distal margin. Forelegs brown. Midlegs brown to bronze; tibia medially white. Hindlegs brown to bronze, tibia base dorsally pale yellow.

***Male genitalia*** (*N* = 1) (Fig. 69). Uncus long, slender, with straight lateral margin, apex narrow, tongue-shaped, dorsally with sclerotised bristles. Gnathos projection limited to small ridge. Valva slender, ventral margin nearly straight, dorsal margin convex, apex rounded. Juxta broad, with base rounded, apex weakly sclerotised, blunt. Saccus small, pointing dorsad. Phallus with broad flat spatula-shaped cornutus.

***Female genitalia*** (*N* = 1) (Fig. 108). Anterior apophyses with dorsal protuberance at posterior 1/3. Antrum membranous. Ductus bursae long, coiled. Corpus bursae globular, with posterior half reticulated, anterior half membranous, with weak sclerotisation between thorn and corpus opening. Thorn long and slender, straight, glabrous.

##### Distribution.

Known from the Fiji (see Remarks), the Aneityum and Tanna islands on Vanuatu, at an altitude of ca. 350 m.

##### Phylogenetic relationships.

*Hoploscopa astrapias* is recovered as sister species (BS = 78). See *H. astrapias* for further remarks.

##### Remarks.

Nuss (1998) raised *H. anamesa* from subspecies of *H. astrapias* to species. He stated that this species occurs in sympatry with *H. astrapias* on Fiji.

#### Hoploscopa nauticorum

Taxon classificationAnimaliaLepidopteraCrambidae

Tams, 1935

E47A3279-5400-5EC1-8D68-722FFDC392B6

Figs 29 , 70

##### Material examined.

***Holotype***: ♂, with labels: “Holo- | type [round label, red ringed]”; “Samoan Is[Land]. [typographed]| Malololelei | Upolu | 24.ii.24. [handwritten]| P.A.Buxton”; “Samoa: | Brit.-Mus. | 1935-315.”; “Hoploscopa | astrapias | nauticorum Tams [typographed] | Holotype ♂ ”; “♂ | Pyralidae | Brit[ish].Mus[eum]. | Slide N°. | 20242”; “GU Nr. 655 | prep. M. Nuß”; "NHMUK 010923388 [barcode appended]”. Deposited in NHMUK.

***Allotype***: ♀, with labels: “Allo- | type [round label, red ringed]”; “Samoan Is. [typographed] | Upolu Is[land] | Malololelei | 21.ii.1925 | 2,000 f[ee]t | P.A. Buxton | and G. H. Hopkins [handwritten]”; “Samoa: | Brit. Mus. | 1935-315.”; “Hoploscopa | astrapias | nauticorum Tams [typographed] | Paratype. | Allotype ♀ [handwritten]”; "NHMUK 010923389 [barcode appended]”. Deposited in NHMUK.

##### Other specimens examined.

2 ♂. Samoa: 2 ♂ (DNA vouchers MTD8252 & MTD8253, genitalia on slide TL725 ♂ & TL726 ♂), West Samoa, Upolu, Tiavi, 600 m, 24.viii.1974, leg. G. S. Robinson (NHMUK).

##### Diagnosis.

The forewings of *H. nauticorum* display snow white median, cubital and dorsal markings together forming a streak disrupted with brown at veins, and a postmedian triangular red patch, distally with snow white streak. In male genitalia, the small sclerotised bristles on dorsal side of the uncus apex are unique to this species. Female genitalia were not investigated.

##### Similar species.

*Hoploscopa astrapias* (q.v.), *H. anamesa* (see *H. astrapias*).

##### Description.

***Head*.** Antennae dorsally bronze. Proboscis brown, speckled with pale yellow. Maxillary palpi dark brown, basally pale yellow, inner side brown. Labial palpi dark brown, ventral base pale yellow.

***Thorax*** (Fig. 29). Collar pale brown. Forewing length: 10–11 mm; forewing ground colour dark brown; basal yellow streak along 1A + 2A, abutted to cubital red fascia running up to postmedian area, disrupted by median cubital patch; small reddish blotch at base of cell, with minute snow white dot; median discoidal stigma trapezoid, red, basally and distally edged with yellow, with snow white streak abutting dorsally, running down to dorsal area, disrupted at veins; postmedian patch quadrangular, red, dorso-distally with thick snow white streak; subterminal line white, indented inwardly at CuA2; subterminal field red; fringe brown. Hindwing pale yellow, bronze toward distal margin. Forelegs brown, tarsi speckled with pale yellow. Midlegs brown; tibia base speckled with pale yellow. Hindlegs brown.

***Abdomen*.** Male sternum A8 posterior margin broadly indented, with short, rounded lateral projections.

***Male genitalia*** (*N* = 3) (Fig. 70). Uncus long and slender, gently tapering toward apex, apex narrow, tongue-shaped, dorsally with few small sclerotised bristles. Gnathos without posterior projection. Valva ventral margin nearly straight, dorsal margin strongly protruding dorsad on basal half, apex rounded. Saccus broad, medially slightly incurved. Phallus with large, flat, spatula-shaped cornutus.

***Female genitalia*.** Not investigated.

##### Distribution.

Known from the island of Upolu on Samoa, at an altitude of ca. 600 m.

##### Phylogenetic relationships.

*Hoploscopa nauticorum* is recovered as sister to *H. astrapias* and *H. anamesa* in the ML analysis of the COI barcode (BS = 61). See *H. astrapias* for further remarks.

##### Remarks.

Nuss (1998) raised *H. nauticorum* from subspecies of *H. astrapias* to species.

#### Hoploscopa anacantha

Taxon classificationAnimaliaLepidopteraCrambidae

Léger & Nuss sp. nov.

6E602DE1-9894-5341-99AA-1DA380B1D952

http://zoobank.org/58D67EDA-0687-479C-87A3-854F58BC369C

Figs 32 , 71 , 111

##### Material examined.

***Holotype***: ♂, with labels: “G[unung]. Mogogonipa | summit, 1008m. | 18–20.x.1985”; “Indonesia : | SULAWESI UTARA, | Dumoga-Bone N[ational]. P[ark]. [Bogani Nani Wartabone National Park] | October 1985.”; “R[oyal]. Ent[omological]. Soc[iety]. Lond[on]. | PROJECT WALLACE | B.M. 1985-10”; "NHMUK010923444 [barcode appended]”; “TL 759 | ♂”. Deposited in NHMUK.

***Paratypes***: 3♂, 3♀ Indonesia: 2♂ (NHMUK010923449 & NHMUK010923343), Sulawesi Utara, Danau Mooat, site 22, PHPA chalet, open habitat, 1080 m, 31.viii.1985, leg. J. D. Holloway; ♂ (NHMUK010923446 & genitalia on slide TL760 ♂), 1 ♀ (NHMUK010923445), Sulawesi Utara, Dumoga-Bone National Park [Bogani Nani Wartabone National Park], “Clarke” Camp, lower montane forest, 1140 m, leg. Royal Entomological Society of London; 2 ♀ (1 with NHMUK010923447, DNA voucher MTD8247 & genitalia on slide TL732 ♀, 1 with NHMUK010923448), Sulawesi Utara, Danau Mooat, near Kotamobagu, 1200 m, 27–28.ix.1985, leg. Royal Entomological Society of London (NHMUK).

##### Diagnosis.

*Hoploscopa anacantha* sp. nov. displays dark brown forewings with basal, median and postmedian white streak-like markings. In male genitalia, the gnathos projection forms a conspicuous triangle reaching half the length of the uncus. In female genitalia, the elongated bulging sclerotisation without thorn on corpus bursae is unique to this species.

##### Similar species.

No similar species known.

##### Description.

***Head*.** Antennae dorsally dark brown. Proboscis white. Maxillary palpi dark brown, basally white. Labial palpi dark brown, ventro-basally white.

***Thorax*** (Fig. 32). Collar white. Forewing length: 9 mm (♂), 9–10 mm (♀); forewing ground colour dark brown; basal cubital streak and median triangular to streak-like patch white, edged with darker brown and few reddish brown scales; postmedian streak white, reddish brown toward middle of wing; fringes dark brown, with white dots. Hindwing pale brown. Legs brown.

***Abdomen*.** Male sternum A8 posterior margin notched, laterally with short, rounded lateral projections.

***Male genitalia*** (*N* = 2) (Fig. 71). Uncus long, slender, with straight lateral margin, apex blunt. Gnathos projection broad, triangular, ca. half the uncus length, with small rounded apex. Valva ventral margin nearly straight, dorsal margin convex, apex pointed. Juxta with base quadrangular, medially narrowed, apex wide, truncate. Saccus small, quadrangular.

***Female genitalia*** (*N* = 1) (Fig. 111). Anterior apophyses without bump at posterior 1/3. Antrum sclerotisation short, as long as broad. Ductus bursae long, broadly curved before corpus opening. Corpus bursae globular, covered with erect papillae, reticulated at corpus opening, with well-marked elongated bulging sclerotisation running from corpus opening to middle of corpus bursae. Thorn absent.

##### Distribution.

Known from North Sulawesi (Indonesia) at altitudes between 1,000 m and 1,200 m.

##### Phylogenetic relationships.

This species is recovered sister to another similar but yet undescribed *Hoploscopa* species (DNA voucher MTD8238) from North Sulawesi (BS=67).

##### Etymology.

From the Greek *a*-, *an*-, without, and *acantha*, spine, referring to the absence of thorn on the corpus bursae of female genitalia. This name was suggested by Francesca Vegliante.

#### Hoploscopa kelama

Taxon classificationAnimaliaLepidopteraCrambidae

Léger & Nuss sp. nov.

5C83BE22-CCCC-5FC5-8390-072DE4DBA89C

http://zoobank.org/69D2A893-2F5A-4E90-9E82-8C6FD059A364

Figs 33 , 72 , 112

##### Material examined.

***Holotype***: ♀, with labels: “[Indonesia] North-Sulawesi, Danau Mooat | east of Kotamobagu, 1150m | 30–31.iii.2000, at light | leg. A. Kallies & S. Naumann”; “DNA voucher | Lepidoptera | MTD 2016 | no. 3214 [vertically written]”; “TL | 532 ♀”. Deposited in MTD.

***Paratypes***: 6 ♂, 2 ♀. 1 ♂ (DNA voucher MTD LEP3215, genitalia on slide TL511 ♂), same data as holotype; 2 ♀ (1 with DNA voucher MTD7878 & genitalia on slide TL665 ♀, 1 with DNA voucher 7879 & genitalia on slide TL474 ♀), same collecting locality as holotype, 25–26.iii.2000, leg. A. Kallies & C. Zorn (MTD); 2 ♂ (NHMUK010923443, NHMUK010923341), North Sulawesi, Danau Mooat, near Kotamobagu, 1200 m, 27–28.ix.1985, leg. Royal Entomological Society of London, Project Wallace; 3 ♂ (NHMUK010923342, NHMUK010923442, NHMUK010923441), North Sulawesi, Dumoga-Bone National Park [Bogani Nani Wartabone National Park], Clarke Camp, lower montane forest, 1140 m, x.1985, leg. Royal Entomological Society of London, Project Wallace (NHMUK).

##### Diagnosis.

*Hoploscopa kelama* sp. nov. displays basal and distal discoidal dark brown spots edged with pale yellow, as well as a pale yellow cubital double line on the forewing. In female genitalia, the very small thorn located at the corpus bursae opening is unique to this species.

##### Similar species.

*Hoploscopa boleta* sp. nov., *H. pseudometacrossa* sp. nov. Both species display light brown median discoidal stigma and postmedian patch and lack the double pale yellow cubital line observed in *H. kelama* sp. nov. Examination of genitalia allows unambiguous separation of these species from *H. kelama* sp. nov.: male genitalia of both species show a slender uncus bearing thick setae, ventrally marked with three small ridges, and female genitalia show a larger thorn located on posterior half of corpus bursae.

##### Description.

***Head*.** Antennae dorsally striped with brown and ochreous scales. Proboscis brown. Maxillary palpi brown, basally pale yellow, inner side pale brown. Labial palpi brown, ventro-basally pale yellow.

***Thorax*** (Fig. 33). Collar white. Forewing length: 8–9.5 mm (♂ & ♀); forewing ground colour brown to dark brown; basal dash black at base, distally pale yellow; basal discoidal patch crescent-shaped, pale yellow, filled with dark brown; distal discoidal patch dark brown, quadrangular, with basal and distal edges pale yellow; double cubital line running from median area distally to tornus, pale yellow; postmedian costal blotch small, pale yellow; subterminal line pale yellow, barely or not marked on its middle fringes brown, with pale yellow dots. Hindwing pale brown. Forelegs brown. Mid- and hindlegs with femur brown; tibia dark brown, distally pale yellow; tarsi brown.

***Abdomen*.** Male sternum A8 posterior margin notched, with short, rounded lateral projections.

***Male genitalia*** (*N* = 1) (Fig. 72). Uncus long and slender, medially slightly widened, conspicuously narrowed at apical 1/4, apex duck beak-shaped, with small median bump. Gnathos projection limited to a small ridge. Valva ventral margin nearly straight, dorsal margin slightly convex, apex pointed. Juxta slender, with base slightly quadrangular, medially narrowed, apex broadly incurved, weakly sclerotised. Saccus quadrangular.

***Female genitalia*** (*N* = 3) (Fig. 112). Anterior apophyses with small dorsal bump at posterior 1/3. Antrum sclerotisation broad, ca. twice as long as broad. Ductus bursae long, broad, looped once, bent at corpus opening. Corpus bursae with posterior half reticulated, anterior half membranous. Thorn located on ductus bursae shortly ahead of corpus opening, very small, with dents pointing toward thorn apex.

##### Distribution.

Known from North Sulawesi (Indonesia) at altitudes between 1,000 m and 1,200 m.

##### Phylogenetic relationships.

The ML analysis of the COI barcode recovered *H. kelama* sp. nov. in a clade with *H. albomaculata* sp. nov. and *H. ignitamaculae* sp. nov., but without significant support (BS = 30). These three species vary greatly in their wing pattern but show similarities in the morphology of the genitalia: the uncus apex is duck-shaped, with a small tip on its middle in male genitalia; in female genitalia, the ductus bursae is long and broad, the corpus bursae is globular, with an elongated, well-marked sclerotisation. *Hoploscopa kelama* sp. nov. and *H. ignitamaculae* sp. nov. share a small-sized thorn on corpus bursae. Hoploscopa*anacantha* sp. nov. shares a wing pattern similar to that of *H. albomaculata* sp. nov., and displays an elongated, marked sclerotisation like in the other three species above, although without thorn.

##### Etymology.

The species epithet *kelama* comes from the Indonesian “kelam” meaning dark, referring to the dark patterns of the forewing.

#### Hoploscopa ignitamaculae

Taxon classificationAnimaliaLepidopteraCrambidae

Léger & Nuss sp. nov.

55C1A884-BA0B-588C-AF5C-71B71C81C396

http://zoobank.org/DB39215F-2066-454D-BEAE-BE7D0E523CF4

Figs 34 , 73 , 113

##### Material examined.

***Holotype***: ♀, with labels: “[Indonesia] North-Sulawesi, Tangkoko- | Batuangus-Dua-Saudara Reserve | near Batuputih, primary forest | 600m, 21.iii.2000, at light | leg. A. Kallies & C. Zorn”; “DNA barcode | BC MTD 01427”. Deposited in MTD.

***Paratypes***: 6 ♂, 13 ♀. Indonesia: 4 ♂ (3 with genitalia on slide TL475 ♂, TL480 ♂, TL545 ♂), 7 ♀ (1 with DNA voucher MTD LEP82, genitalia on slide TL364 ♀; 1 with DNA barcode BC MTD 01425), same data as holotype; 3 ♀ (1 with DNA voucher MTD LEP3216, DNA barcoding BC MTD 01426; 1 with DNA voucher MTD LEP3218, genitalia on slide TL516 ♀), North Sulawesi, Danau Mooat, east of Kotamobagu, 1000 m, 25–26.iii.2000, at light, leg. A. Kallies & C. Zorn; 1 ♂ (DNA voucher MTD LEP3217 & genitalia on slide TL509 ♂) with same collecting locality, 30–31.iii.2000, leg. A. Kallies & S. Naumann (MTD); 1 ♂ (NHMUK010923412), North Sulawesi, Dumoga-Bone National Park [Bogani Nani Wartabone National Park], Clarke Camp, lower montane forest, 1140 m, ix.1985, leg. Royal Entomological Society of London, Project Wallace; 1 ♀ (NHMUK010923411), same data except Plot B, lowland forest, 300 m; 2 ♀ (NHMUK010923410, NHMUK010923413), same data except Gunung Mogogonipa, summit, 1008m, 22–23.ix.1985 (NHMUK).

##### Diagnosis.

The forewings of *H. ignitamaculae* sp. nov. display marked basal, median and postmedian reddish orange markings edged with yellow; postmedian patch with undulated basal margin. In male genitalia, the uncus is medially widened, its apex is duck-shaped, ventrally with small bump, and the gnathos is projected into a short, pointed tip. In female genitalia, the well-defined elongated sclerotisation of the corpus bursae with the small slightly curved thorn on its middle is unique to this species.

##### Similar species.

*Hoploscopa isarogensis* sp. nov. Forewing markings of *H. isarogensis* sp. nov. tend more to red. Postmedian patch is triangular with a straight basal margin, and subterminal line is often markedly pale yellow. Examination of genitalia allow unambiguous separation of these species.

##### Description.

***Head*.** Antennae dorsally with bronze scales. Proboscis white to pale yellow. Maxillary palpi brown, basally pale yellow. Labial palpi brown, ventral base and inner side pale yellow.

**Figures 34–45.**
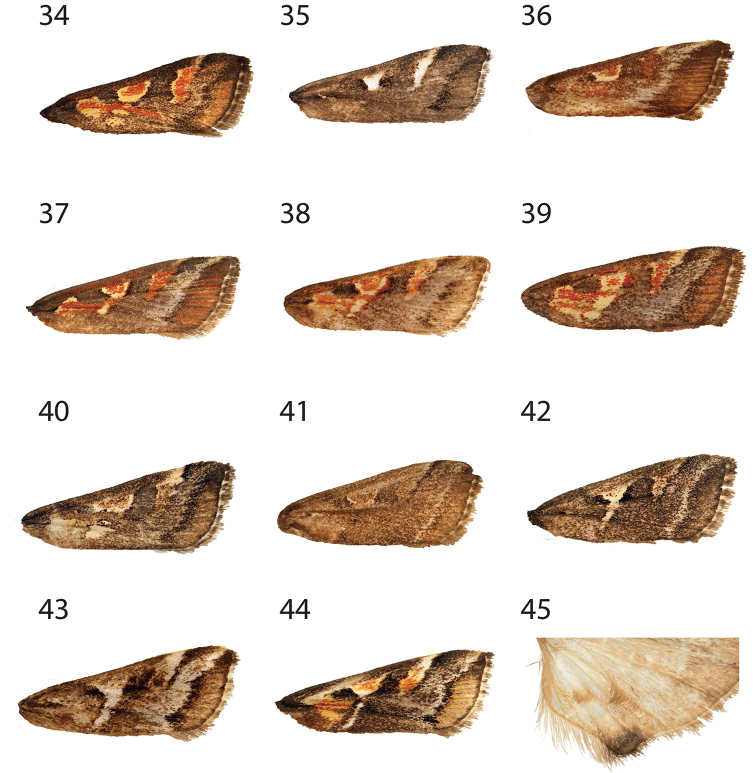
Forewing of *Hoploscopa* species. **34***Hoploscopa ignitamaculae* sp. nov., paratype, ♂, North Sulawesi, Danau Mooat, east of Kotamobagu, 1000 m, 30–31.iii.2000 (A. Kallies & C. Zorn) (genitalia on slide TL509 ♂) **35***Hoploscopa albomaculata* sp. nov., paratype, ♀, Indonesia, North Sulawesi, Danau Mooat, east of Kotamobagu, 1000 m, 25–26.iii. 2000, (A. Kallies & C. Zorn) (genitalia on slide TL540 ♀) **36***Hoploscopa sumatrensis* sp. nov., holotype, ♂, Indonesia, Sumatra-Holzweg, 25 km SSW-Pematangsiantar, road to Prapat, 6–26.i.1996 (A. Kallies) (genitalia on slide TL755 ♂) **37***Hoploscopa mallyi* sp. nov., paratype, ♂, Malaysia, Sabah, Mt Kinabalu, Mesilau, 2000 m, 14–17.xi.2006 (W. Mey & K. Ebert) **38***Hoploscopa gracilis* sp. nov., holotype, ♂, Indonesia, Sumatra-Holzweg, 25 km SSW-Pematangsiantar, road to Prapat, 13.ii.1996 (A. Kallies) (genitalia on slide TL754 ♂) **39***Hoploscopa agtuuganonensis* sp. nov., holotype, ♀, Philippines, Mindanao, Mt Agtuuganon, 1050 m, 28.v–7.vi.1996 (W. Mey) (genitalia on slide TL616 ♀) **40***Hoploscopa boleta* sp. nov., paratype, ♀, Papua New Guinea, Morobe, Mt Kaindi, 2360 m, 3.x.1992 (V. O. Becker) (genitalia on slide TL448 ♀) **41***Hoploscopa pseudometacrossa* sp. nov., holotype, ♀, Papua New Guinea, W. Hhl, Prv, near Mt. Hagen, Kuk Ag, Res. Sta., 1600 m, 19–20.viii.1983 (Scott E. & Pamela Miller) (genitalia on slide TL689 ♀) **42***Hoploscopa metacrossa* Hampson, ♀, Papua New Guinea, Morobe Province, near Bulolo, Mt Susu National Reserve, *Araucaria* forest, 975 m, 27–28.viii.1983 (S. Miller). **43***Hoploscopa jubata* sp. nov., paratype, ♂, Papua New Guinea, Morobe Province, near Bulolo, Mt Susu National Reserve, *Araucaria* forest, 975 m, 27–28.viii.1983 (S. Miller) **44***Hoploscopa jubata* sp. nov. , paratype, ♀, Morobe Province, near Bulolo, Mt Susu National Reserve, *Araucaria* forest, 975 m, 27–28.viii.1983 (S. Miller) **45** Hindwing scent organ in *Hoploscopa metacrossa*. Paratype, ♂, Papua New Guinea, Morobe Province, Wau, 1200 m, 8–14.xii.1976 (G. F. Hevel & R. E. Dietz) (genitalia on slide TL443 ♂).

***Thorax*** (Fig. 34). Collar white. Forewing length: 8.0–9.5 mm (♂ & ♀); forewing ground colour brown; basal patch yellow, with cubital reddish orange line starting from its middle, running distally up to postmedian area; costal field reddish orange; median discoidal stigma trapezoid, reddish orange, edged with yellow; median cubital and dorsal patches yellow, not connected; postmedian patch reddish orange, basal margin undulated, yellow, costal blotch yellow; subterminal field brown to tawny; fringes brown with pale yellow spots. Hindwing pale brown. Forelegs brown. Midlegs brown; tibia distally pale yellow. Hindlegs with femur brown; tibia pale yellow speckled with brown; tarsi brown.

***Abdomen*.** Male sternum A8 posterior margin broadly incurved, with short, rounded lateral projections.

***Male genitalia*** (*N* = 4) (Fig. 73). Uncus medially widened, apex duck beak-shaped, ventrally with a small bump. Gnathos projection triangular, slender, ca. 1/4 of uncus length, with rounded apex. Valva ventral margin nearly straight, dorsal margin convex, apex pointed. Juxta with rounded base, narrowing on basal half, apex blunt. Saccus slightly quadrangular.

***Female genitalia*** (*N* = 2) (Fig. 113). Anterior apophyses with dorsal bump at posterior 1/3. Antrum sclerotisation short, as long as wide. Ductus bursae of medium length, broad, bent at anterior and posterior 1/4. Corpus bursae globular, posterior half reticulated, anterior half with erected papillae, with elongated sclerotised area running from corpus opening to its middle. Thorn on middle of sclerotisation, very small, slightly curved, with dents pointing toward thorn base.

##### Distribution.

Known from North Sulawesi (Indonesia) at altitudes between 300 m and 1,150 m.

##### Phylogenetic relationships.

See *H. kelama* sp. nov.

##### Etymology.

From the Latin *ignitus*, set on fire, and *macula*, spot or blot, in reference to the red and yellow spots of the forewing.

##### Remarks.

Specimen MTD8238 shows a forewing pattern resembling that of *H. ignitamaculae* sp. nov., but with markings reduced. However, the thorn is absent in female genitalia.

#### Hoploscopa albomaculata

Taxon classificationAnimaliaLepidopteraCrambidae

Léger & Nuss sp. nov.

1AFD6A3A-490A-5DE3-B75A-03256651A683

http://zoobank.org/E458DE68-F0CC-48B1-91D2-8F94B21D51DB

Figs 35 , 74 , 114

##### Material examined.

***Holotype***: ♀, with labels: “[Indonesia] North Sulawesi, Danau Mooat | east of Kotamobagu, 1000m | 25.–26. iii. 2000, at light | leg. A. Kallies & C. Zorn”; “DNA Barcode | BC MTD 01429”; “TL544 | ♀”. Deposited in MTD.

***Paratypes***: 9 ♂, 5 ♀. Indonesia: 5 ♂ (4 with genitalia on slides TL473 ♂, TL479 ♂, TL543 ♂, TL646 ♂), 3 ♀ (1 with DNA voucher MTD LEP57 & MTD LEP3213, genitalia on slide TL529 ♀), same data as holotype; 2 ♂ (1 with DNA voucher MTD LEP81, genitalia on slide TL363 ♂), 1 ♀ (DNA barcoding BC MTD 01428, genitalia on slide TL540 ♀), same locality as holotype, 30–31.iii.2000, leg. A. Kallies & S. Naumann (MTD); 1 ♂ (NHMUK010923450), 1 ♀ (NHMUK010923355), North Sulawesi, Danau Mooat, 1200 m, near Kotamobagu, 27–28.ix.1985 (♂), 9.xi.1985 (♀), leg. Royal Entomological Society of London, Project Wallace; 1 ♂ (NHMUK010923451), same data, Site 22, 1080 m, PHPA chalet, open habitat, 31.viii.1985, leg. J. D. Holloway (NHMUK).

##### Diagnosis.

*Hoploscopa albomaculata* sp. nov. is unique by virtue of its median trapezoid and postmedian streak-like white patches on the forewing. In male genitalia, the gnathos projection is reduced to a small triangular tip, and the juxta is elongated, slender, with a deeply indented apex. In female genitalia, ductus bursae is long, broad and curvy, and corpus bursae displays well-delimited sclerotised band and a straight thorn.

##### Similar species.

No similar species known.

##### Description.

***Head*.** Antennae dorsally with brown scales. Proboscis pale yellow to pale brown. Maxillary palpi brown, base and inner side pale yellow. Labial palpi brown, ventral base and inner side pale yellow.

***Thorax*** (Fig. 35). Collar pale yellow. Forewing length: 9 mm (♂), 9–10 mm (♀); forewing ground colour brown; small basal white dash edged with dark brown; median discoidal stigma trapezoid, white, with basal and distal edges dark brown; postmedian patch streak-like, white, pale yellow at costa, edges dark brown; subterminal line dark brown, basally faintly marked with pale yellow; fringes basally pale yellow, distally brown. Hindwing pale brown. Forelegs bronze. Mid- and hindlegs with femur brown; tibia pale yellow speckled with brown; tarsi bronze.

***Abdomen*.** Male sternum A8 posterior margin bilobed, laterally with short, rounded lateral projections.

***Male genitalia*** (*N* = 5) (Fig. 74). Uncus medially slightly widened, narrowed at apical 1/4, apex roughly truncate, ventrally with a triangular tip pointing posterad. Gnathos projection short, wide at base, triangular. Valva ventral margin nearly straight, dorsal margin conspicuously convex, apex pointed. Juxta slender, with base rounded, narrowing toward apex, apex deeply indented. Saccus small, pointing dorsad.

***Female genitalia*** (*N* = 3) (Fig. 114). Anterior apophyses without dorsal bump at posterior 1/3. Ductus bursae long, slender, gently curved twice. Antrum sclerotisation short, as long as wide. Corpus bursae small, globular, reticulated, with marked elongated sclerotisation from corpus opening to thorn. Thorn straight, with small dents pointing toward thorn apex.

##### Distribution.

Known from North Sulawesi (Indonesia) at altitudes between 1,000 m and 1,200 m.

##### Phylogenetic relationships.

See *H. kelama* sp. nov.

##### Etymology.

From the Latin *albus*, white, and *maculatus*, covered with spots.

**Figures 46–51.**
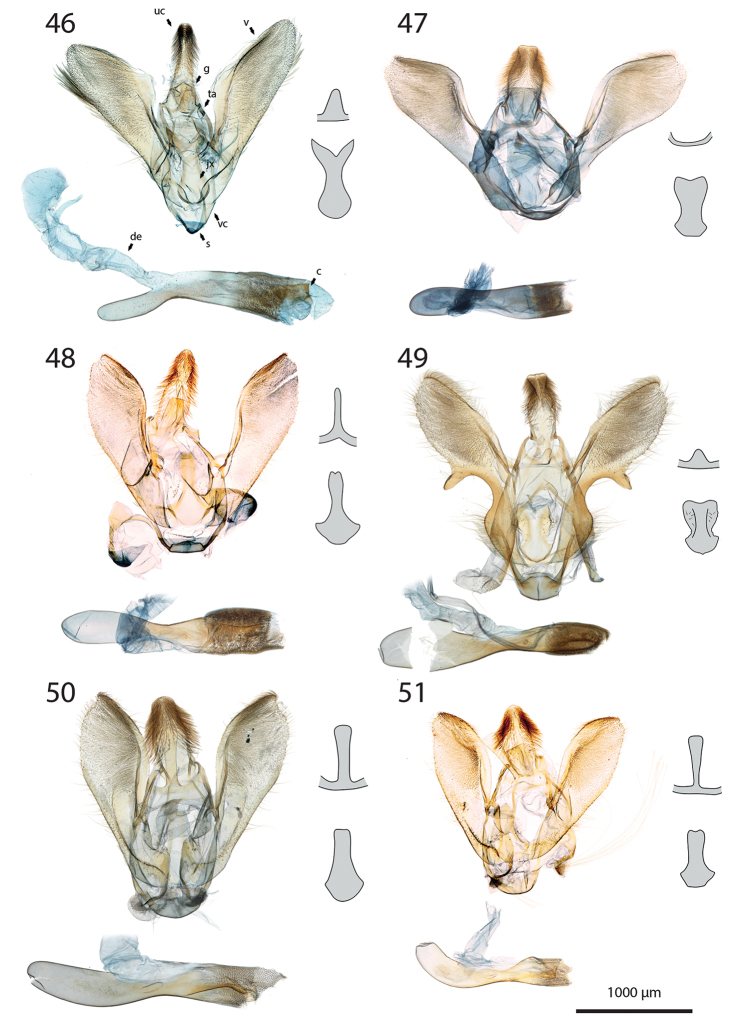
Male genitalia features of *Hoploscopa*. For each figure left above: genitalia without phallus; left below: phallus; right above: gnathos drawing; right below: juxta drawing. **46***Hoploscopa albipuncta* sp. nov., paratype, TL336 ♂ (genitalia without phallus), TL635 ♂ (phallus) **47***Hoploscopa matheae* sp. nov., paratype, TL730 ♂ **48***Hoploscopa sepanggi* sp. nov., paratype, TL641 ♂ **49***Hoploscopa cynodonta* sp. nov., paratype, TL531 ♂ **50***Hoploscopa parvimacula* sp. nov., holotype, TL510 ♂ **51***Hoploscopa kinabaluensis* sp. nov., paratype, TL342 ♂. Abbreviations: c (cornutus), de (ductus ejaculatorius), g (gnathos), jx (juxta), s (saccus), ta (tegumen arm), v (valva), vc (vinculum), uc (uncus).

#### Hoploscopa sumatrensis

Taxon classificationAnimaliaLepidopteraCrambidae

Léger & Nuss sp. nov.

59060E37-9381-50B0-A555-47C6757449BA

http://zoobank.org/22F33777-64CB-4E37-9AB2-41A52020ABEB

Figs 36 , 75 , 83 , 115

##### Material examined.

***Holotype***: ♂, with labels: “Sumatra-Holzweg | 25km SSW-Pematangsiantar | Straße nach Prapat [road to Prapat] | 6.-26.i.1995, leg. A. Kallies”; “coll[ection]. M. Nuss | Geschenk 2000 | Museum für Tier- | kunde Dresden”; “DNA voucher | Lepidoptera | date: xi.2018 | MTD8260 | [vertically written:] DNA | -voucher”; “TL755 | ♂”. Deposited in MTD.

***Paratypes***: 3 ♂, 4 ♀. Indonesia. 3 ♂ (DNA vouchers MTD9119, MTD9129 & MTD9121 and genitalia on slides TL761 ♂, TL763 ♂, TL538 ♂), 3 ♀ (DNA vouchers MTD LEP3205, MTD8257, MTD8262 & genitalia on slides TL524 ♀, TL752 ♀, TL757 ♀), same data as holotype; 1 ♀ (DNA voucher MTD7880 & genitalia on slide TL670 ♀), Sumatra, Asahan, Huta Padang, 1990, leg. E. W. Diehl (MTD).

##### Other specimens examined.

4 ♀, 2 ♀ (DNA vouchers MTD LEP3202, MTD 8258 & genitalia on slides TL530 ♀, TL753 ♀), same data as holotype; 1 ♀ (DNA voucher MTD LEP3210 & genitalia on slide TL522 ♀), same data as holotype except 23.viii.1989, leg. E. W. Diehl; 1 ♀ (DNA voucher MTD8261 & genitalia on slide TL756 ♀), same data as holotype except 13.ii.1996 (MTD).

##### Diagnosis.

The forewings of *H. sumatrensis* sp. nov. display a pale yellow crescent-shaped median discoidal stigma filled with reddish brown, as well as a postmedian area broadly suffused with pale yellow. In male genitalia, the gnathos projection is slender, ca. 4/5 of uncus length, with a tongue-shaped apex, and the juxta has a rounded base and a notched apex. In female genitalia, the short and slender ductus bursae bent before corpus and the pear-shaped corpus bursae with small straight thorn resemble those of other *Hoploscopa* species, e.g., *H. danaoensis* sp. nov. and *H. parvimacula* sp. nov.

##### Similar species.

*Hoploscopa pangrangoensis* sp. nov. (q.v.).

##### Description.

***Head*.** Antennae dorsally with brown scales. Proboscis pale yellow to brown. Maxillary palpi brown, base and inner side pale yellow. Labial palpi brown, ventral base and inner side pale yellow.

***Thorax*** (Fig. 36). Collar pale yellow. Forewing length: 9–10 mm (♂ & ♀); forewing ground colour brown; small basal dark brown dash distally pale yellow; base of dorsum with patch of pale yellow scales; cubital reddish brown fascia running up to median area; median discoidal stigma reddish brown, edged with crescent-shaped pale yellow patch; postmedian patch reddish brown, with pale yellow blotch at costa; subterminal line broad, pale yellow; subterminal field tawny to brown; fringes brown, with pale yellow dots. Hindwing pale yellow. Forelegs bronze. Midlegs with femur brown; tibia pale yellow to brown; tarsi bronze. Hindlegs brown; tibia pale yellow speckled with brown; tarsi bronze.

***Abdomen*** (Fig. 83). Male sternum A8 posterior margin bilobed.

***Male genitalia*** (*N* = 4) (Fig. 75). Uncus medially broad, narrowed at apical 1/4, apex tongue-shaped. Gnathos projection slender, ca. 4/5 of uncus length, apex tongue-shaped. Valva ventral margin straight, bent dorsad on distal 1/4, dorsal margin convex, apex pointed. Juxta base rounded, distal half narrowed, apex notched. Saccus triangular, pointed.

***Female genitalia*** (*N* = 4) (Fig. 115). Anterior apophyses with dorsal tip at posterior 1/3. Antrum sclerotisation short. Ductus bursae short, bent before corpus opening. Corpus bursae large, pear-shaped, with sclerotisation between thorn and corpus opening and a median sclerotised band. Thorn small, straight, with small dents.

##### Distribution.

Known from North Sumatra (Indonesia).

##### DNA barcoding.

Two MOTUs are found in morphologically identical specimens from the same locality. The K2P-distance between the two MOTUs is 4.1–6%. The first MOTU, which is the one of the type material, shows an intraspecific variation of 0.7%. The second MOTU is found in three females (samples BC MTD LEP01422, MTD8258 and MTD8261) and shows an intraspecific divergence of 0.6%.

##### Etymology.

Named after the island of Sumatra where the species is encountered.

##### Remarks.

Future examination of male specimens from the second lineage will help to determine whether or not it represents a separate species.

**Figures 52–57.**
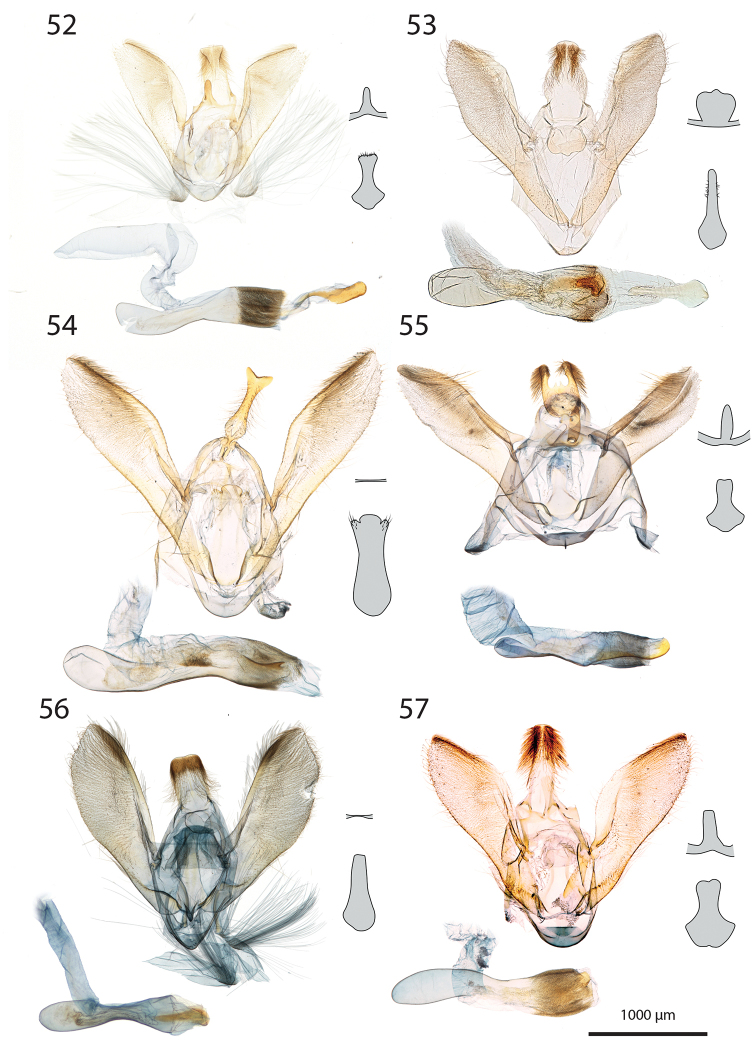
Male genitalia features of *Hoploscopa*. **52***Hoploscopa luteomacula* Nuss, holotype, GU prep. Nuss 744 ♂ **53***Hoploscopa obliqua*, holotype, Pyralidae Brit. Mus. Slide N° BMNH20252 ♂ (phallus with juxta attached) **54***Hoploscopa gombongi* sp. nov., holotype, TL653 ♂ **55***Hoploscopa tonsepi* sp. nov., paratype, TL661 ♂ **56***Hoploscopa niveofascia* sp. nov., holotype, TL442 ♂ **57***Hoploscopa marijoweissae* sp. nov., holotype, TL710 ♂.

#### Hoploscopa mallyi

Taxon classificationAnimaliaLepidopteraCrambidae

Léger & Nuss sp. nov.

1D67A747-BF03-5E80-82D2-8B0E052D6243

http://zoobank.org/13552C8E-ED5E-447B-859A-8402DD48BA72

Figs 37 , 76 , 116

##### Material examined.

***Holotype***: ♀. “Borneo, Mt Kinabalu | Headquarter, 1600m | 10–13.XI.2006, L[icht]F[ang] | leg. W. Mey & K. Ebert”; “Liwagu River | 1500m, Turm”; “DNA voucher | Lepidoptera | M. Nuss 2007 | [vertically written:] no. 124”; “TL366 | ♀”. Deposited in BORN.

***Paratypes***: 4 ♂, 2 ♀. Malaysia: 1 ♂, 1 ♀ (genitalia on slides TL533 ♂ and TL340 ♀ respectively), Sabah, Kinabalu National Park Headquarters, 250 m from Pandanus Trail starting point, 6°0'34"N, 116°32'20"E 1640 m, UV light, 07.vi.2015, leg. T. Léger & R. Mally; 2 ♂ (1 with genitalia on slide TL339 ♂), Sabah, Mesilau logging site, 400 m before entrance to Mesilau Nature Reserve, 6°2'22"N, 116°35'54"E, 1930 m, UV light, 02.vi.2015, T. Léger & R. Mally; 1 ♀ (DNA voucher 3197, genitalia on slide TL513 ♀), Sabah, Kinabalu Park Headquarters, ca. 300 m from starting point of Kiau View Trail, 6°0'25"N, 116°32'21"E, 1660 m, UV light, 06.vi.2015, T. Léger & R. Mally (MTD); 1 ♂, Borneo, Mt Kinabalu, Mesilau, 2000 m, 14–17.xi.2006, W. Mey & K. Ebert (MFNB).

##### Diagnosis.

The forewings of *H. mallyi* sp. nov. display pale yellow crescent-shaped median discoidal stigma filled with red, together with median cubital patch forming a Y. In male genitalia, the gnathos projection is long, slender, reaching circa 2/3 of the uncus length, and the juxta is ogive-shaped, with a slightly indented apex. In female genitalia, the ductus bursae is long, broad, curved twice, and the corpus bursae is small, globular, with one short curved thorn.

##### Similar species.

*Hoploscopa agtuuganonensis* sp. nov., *H. gracilis* sp. nov. In *H. agtuuganonensis* sp. nov., median discoidal stigma together with cubital and dorsal patches forms a band progressively narrowing toward dorsum (median cubital patch reduced to small streak, median dorsal patch absent in *H. mallyi* sp. nov.). *Hoploscopa gracilis* sp. nov. displays wing pattern similar to *H. mallyi* sp. nov. and can be best separated by examination of genitalia: the uncus and valva are slenderer, the valva apex is pointed (rounded in *H. mallyi* sp. nov.) and the juxta displays a broad duck beak-shaped apex. In female genitalia of *H. gracilis* sp. nov., the ductus bursae shows a narrow loop at posterior 1/3, and the thorn is less curved.

##### Description.

***Head*.** Antennae dorsally with brown scales. Proboscis brown to pale brown scaled. Maxillary palpi brown, base and inner side pale yellow. Labial palpi brown, ventral base and inner side pale yellow.

***Thorax*** (Fig. 37). Collar pale yellow. Forewing length: 10–12 mm (♂), 11–12 mm (♀); forewing ground colour brown; basal longitudinal red streak basally edged with pale yellow; costal field red; median discoidal stigma crescent-shaped, pale yellow, filled with red; median cubital patch streak-like, pale yellow, together with discoidal stigma forming a Y; postmedian patch triangular, red, basal edge partially pale yellow, with pale yellow blotch at costa; postmedian area suffused with pale yellow and iridescent scales; subterminal field marked with red; fringes brown with pale yellow dots. Hindwing pale brown. Forelegs brown. Midlegs brown; femur and tibia distally pale yellow. Hindlegs with femur brown; tibia pale yellow speckled with brown; tarsi pale yellow to pale brown.

***Male genitalia*** (*N* = 2) (Fig. 76). Uncus slender, medially narrowed, apex spatulate. Gnathos projection slender, ca. 2/3 of uncus length, apex blunt. Valva ventral margin gently bent dorsad on distal 1/3, dorsal margin convex, apex rounded. Juxta ogival, with base quadrangular, gently narrowing toward apex, apex narrow, notched. Saccus triangular, pointing dorsad.

***Female genitalia*** (*N* = 3) (Fig. 116). Anterior apophyses with dorsal bump at posterior 1/3. Antrum sclerotisation forming a short ring. Ductus bursae long, broad, forming two large curves. Corpus bursae small, globular, reticulate, sclerotised between thorn and corpus opening, medially with broad faintly sclerotised band. Thorn sabre-like, curved, with small dents pointing toward thorn base, basally with conspicuous outwardly projected extension.

##### Distribution.

Known from the slopes of Mount Kinabalu on Borneo, at altitudes between 1,600 m and 2,000 m.

##### Phylogenetic relationships.

This species is recovered in a clade with *H. agtuuganonensis* sp. nov. and *H. gracilis* sp. nov. in the ML analysis of the COI barcode (BS = 92). The morphology of these species is in agreement with the molecular findings: in male genitalia, gnathos projection is long and slender; in female genitalia, the antrum sclerotisation is short, the ductus bursae is relatively broad, looped or with conspicuous curves, and the corpus bursae is small, globular.

##### Etymology.

This species is dedicated to our colleague and friend Richard Mally, an eminent lepidopterologist.

**Figures 58–63.**
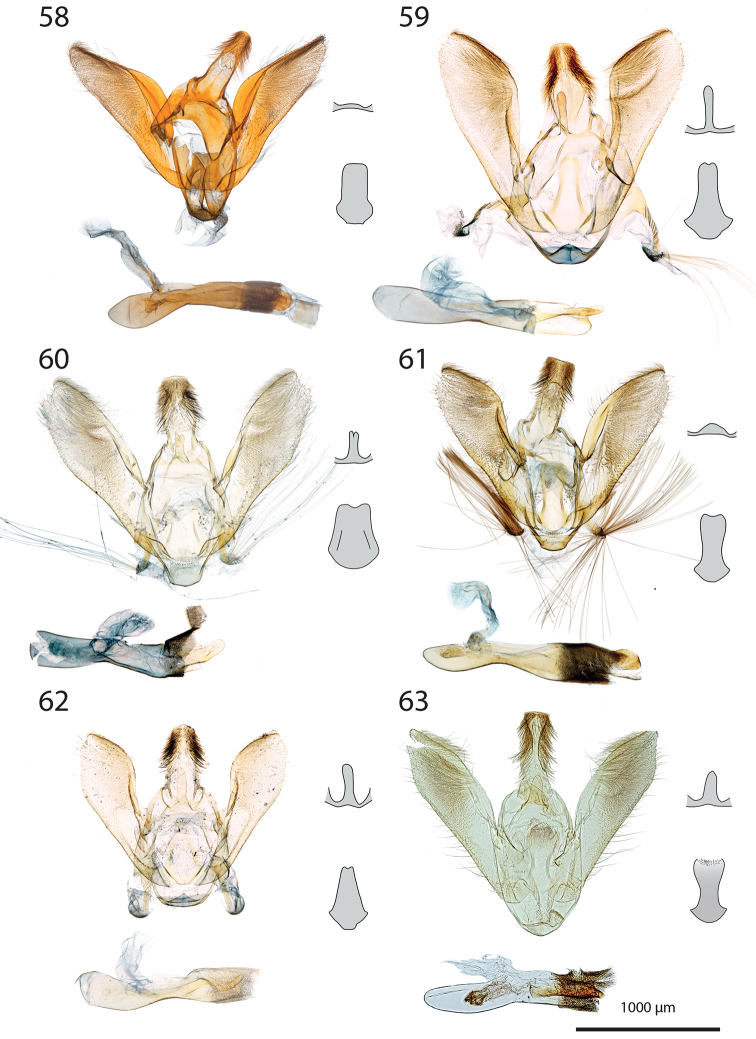
Male genitalia features of *Hoploscopa*. **58***Hoploscopa titika* sp. nov., holotype, TL505 ♂ (apical part of juxta attached to phallus) **59***Hoploscopa pangrangoensis* sp. nov., paratype, TL659 ♂ **60***Hoploscopa isarogensis* sp. nov., paratype, TL762 ♂ **61***Hoploscopa ypsilon* sp. nov., paratype, TL624 ♂ **62***Hoploscopa danaoensis* sp. nov., holotype, TL632 ♂ **63***Hoploscopa quadripuncta*, holotype, Pyralidae Brit. Mus. Slide N° BMNH20257 ♂.

#### Hoploscopa gracilis

Taxon classificationAnimaliaLepidopteraCrambidae

Léger & Nuss sp. nov.

E5D04B10-3A69-529D-8730-D7B1A1540D44

http://zoobank.org/CDC1F535-1AB1-469F-B6DE-082810E8E28F

Figs 38 , 77 , 117

##### Material examined.

***Holotype***: ♂, with labels: “Sumatra-Holzweg | 25km SSW-Pematangsiantar | Straße nach Prapat [road to Prapat], L[icht]F[ang] [light trap] | 13.ii.1996, leg. A. Kallies”; “coll[ection]. M. Nuss | Geschenk 2000 | Museum für Tier- | kunde Dresden”; “DNA voucher | Lepidoptera | date: xi.2018 | MTD8259 | [vertically written:] DNA- | voucher”; “TL754 | ♂”. Deposited in MTD.

***Paratypes***: 4 ♂, 2 ♀. Indonesia: 2 ♂ (1 with DNA voucher MTD7876 & genitalia on slide TL664 ♂, 1 with DNA voucher MTD LEP3203 & genitalia on slide), North Sumatra, Sipirok, 1450 m, 27–28.i.1995, leg. A. Kallies; 1 ♂ (DNA voucher MTD8256 & genitalia on slide TL751 ♂), North Sumatra, Mount Sibayak, 03°14'19"N, 98°29'52"E, 1900 m, 02.iii.2002, leg. U. Buchsbaum; 1 ♂ (genitalia on slide TL539 ♂), 1 ♀ (DNA voucher MTD8263 & genitalia on slide TL758 ♀), North Sumatra, Mount Sibayak, 03°14'13"N, 98°29'41"E, 1750 m, 07.vii.2000, leg. U. Buchsbaum; 1 ♀ (DNA voucher MTD LEP3204 & genitalia on slide TL527 ♀), North Sumatra, Dairi, near Sumbul, 2°46'N, 98°32'E, 1670 m, 20.ii.1999, leg. U. Buchsbaum.

##### Other specimens investigated.

1 ♀. Malaysia: 1 ♀ (NHMUK010923415, DNA voucher MTD8239 & genitalia on slide TL737 ♀), West Malaysia, Cameron Highlands, Gunung Brinchang, 1980 m, 23–31.x.1989 (G. S. Robinson & M. A. Tobin) (NHMUK).

##### Diagnosis.

*Hoploscopa gracilis* sp. nov. displays pale yellow crescent-shaped median discoidal stigma filled with reddish brown, together with median cubital patch forming a Y. In male genitalia, uncus is long and slender, valva is slender with pointed apex, and juxta displays a broad duck beak-shaped apex. In female genitalia, the ductus bursae is long, forming one loop on its middle, and the corpus bursae is small, globular, with one short slightly curved thorn.

##### Similar species.

*Hoploscopa agtuuganonensis* sp. nov., *H. mallyi* sp. nov. (q.v.). Median markings of the forewing form a band progressively narrowing toward dorsum in *H. agtuuganonensis* sp. nov. In male genitalia, uncus of *H. agtuuganonensis* sp. nov. is larger, valva has a rounded apex and juxta apex is tongue-shaped. Female genitalia of *H. agtuuganonensis* sp. nov. are very similar to those of *H. gracilis* sp. nov. but differ by a more marked corpus sclerotisation and a thicker thorn.

##### Description.

***Head*.** Antennae dorsally ochreous to brown. Proboscis pale yellow. Maxillary palpi brown, base and inner side pale yellow. Labial palpi brown, ventral base and inner side pale yellow.

***Thorax*** (Fig. 38). Collar pale yellow. Forewing length: 9 mm (♂), 11 mm (♀); forewing ground colour brown; broad elongated basal patch reddish brown with basal edge pale yellow, distally running up to median cubital patch; costal field reddish brown; median discoidal stigma crescent-shaped, pale yellow, filled with reddish brown; median cubital patch streak-like, pale yellow, disrupted at 1A+2A, together with median discoidal stigma forming a Y; postmedian triangular patch reddish brown, speckled with pale yellow, with pale yellow blotch at costa; postmedian area suffused with a mix of pale yellow and iridescent scales; subterminal field marked with reddish brown; fringes brown, with pale yellow dots. Hindwing pale yellow. Forelegs brown. Midlegs brown; tibia distally pale yellow. Hindlegs brown, tibia pale yellow speckled with brown.

***Abdomen*.** Male sternum A8 posterior margin notched, with short, rounded lateral projections.

***Male genitalia*** (*N* = 4) (Fig. 77). Uncus long and slender, gently tapering toward apex, apex spatulate. Gnathos projection slender, ca. 2/3 of uncus length, with truncate apex. Valva slender, ventral margin nearly straight, gently bent dorsad on distal 1/4, dorsal margin convex, apex pointed. Juxta with base rounded, medially straight, widening at distal 1/3 onto duck beak-shaped apex. Saccus triangular, conspicuously pointing dorsad.

***Female genitalia*** (*N* = 2) (Fig. 117). Anterior apophyses with dorsal bump at posterior 1/3. Antrum sclerotisation as long as wide. Ductus bursae long, with one loop, curved before corpus opening. Corpus bursae small, globular, reticulated, with sclerotisation between thorn and corpus opening and faintly sclerotised band medially. Thorn small, plump, slightly curved, with small dents pointing toward thorn base, basally with small outwardly projected extension.

##### Distribution.

Known from North Sumatra, at altitudes between ca. 1,200 m to 1,900 m.

##### DNA barcoding.

The species shows an intraspecific variation of 0.9%

##### Phylogenetic relationships.

See *H. mallyi* sp. nov.

##### Etymology.

From the Latin *gracilis*, slender, narrow, referring to the slender shape of the uncus, the gnathos projection, and the valva in male genitalia.

**Figures 64–69.**
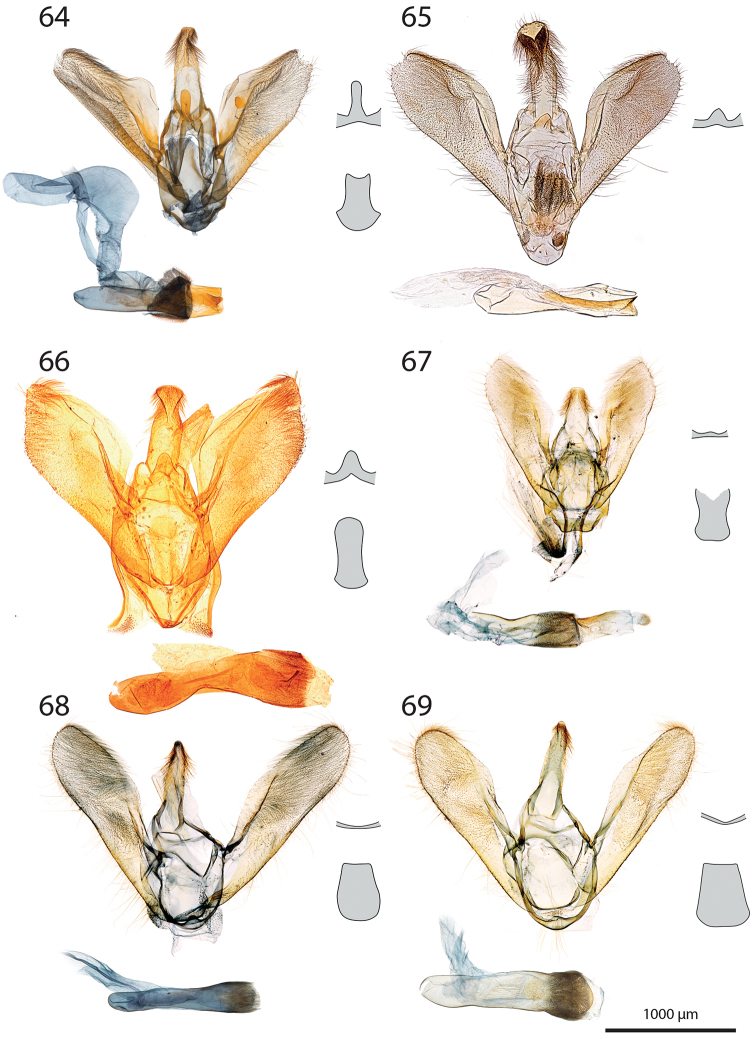
male genitalia features of *Hoploscopa*. **64***Hoploscopa subvariegata*, TL534 ♂ **65***Hoploscopa persimilis*, lectotype, Pyralidae Brit. Mus. Slide N° BMNH20255 ♂ (no juxta drawing) **66***Hoploscopa diffusa*, holotype, Pyralidae Brit. Mus. Slide N° BMNH1014 ♂ **67***Hoploscopa triangulifera*, TL711 ♂ **68***Hoploscopa astrapias*, TL724 ♂ **69***Hoploscopa anamesa*, TL727 ♂.

#### Hoploscopa agtuuganonensis

Taxon classificationAnimaliaLepidopteraCrambidae

Léger & Nuss sp. nov.

5CD9D333-3091-550D-89A4-C0BC4535151D

http://zoobank.org/EA83FE68-F42D-4481-AB44-D1258E5DEBD4

Figs 39 , 78 , 118

##### Material examined.

***Holotype***: ♀, with labels: “Philippinen | Mindanao, 1050m | Mt Agtuuganon | 28.5–7.6.[19]96, leg. MEY”; “DNA voucher | Lepidoptera | date: i.2018 | MTD7416 | [vertically written:] DNA-voucher”; “TL | 616 ♀”. Deposited in MFNB.

***Paratypes***: 12 ♂ , 4 ♀. Philippines: 12 ♂(1 with DNA voucher MTD8149 & genitalia on slide TL708 ♂, 2 with genitalia on slide TL675 ♂, TL676 ♂), 4 ♀ (1 with DNA voucher MTD7415 & genitalia on slide TL617 ♀), same data as holotype (MFNB).

##### Diagnosis.

In *H. agtuuganonensis* sp. nov., median markings of the forewing form together a pale yellow band progressively narrowing toward dorsum, faintly disrupted with red at veins. In male genitalia, uncus is long, slender, narrowed at apical 1/4 and gnathos projection is long and slender. In female genitalia, ductus bursae is long, broad, with one loop, and corpus bursae is small, globular, with one short plump thorn.

##### Similar species.

*Hoploscopa isarogensis* sp. nov. (q.v.), *H. mallyi* sp. nov. (q.v.), *H. gracilis* sp. nov. (q.v.).

##### Description.

***Head*.** Antennae dorsally striped with brown and pale yellow scales. Proboscis pale yellow to pale brown. Maxillary palpi brown, base and inner side pale yellow. Labial palpi brown, ventral base and inner side pale yellow.

***Thorax*** (Fig. 39). Collar pale yellow to white. Forewing length: 8–9 mm (♂), 9 mm (♀); forewing ground colour brown; basal reddish brown quadrangular patch basally edged with pale yellow, crossed by longitudinal pale yellow streak, with median cubital and dorsal patches abutting dorsally; costal field reddish brown; median discoidal stigma trapezoid, pale yellow, filled with reddish brown, basal and distal edges marked with reddish brown, together with two pale yellow cubital patches forming a broad band narrowing toward dorsum; postmedian patch triangular, reddish brown, speckled with pale yellow, with pale yellow blotch at costa; postmedian area suffused with pale yellow; subterminal line pale yellow; subterminal field reddish brown; fringes brown, with large pale yellow spots. Hindwing pale brown. Fore- and midlegs brown. Hindlegs with femur brown; tibia pale yellow speckled with brown; tarsi brown.

***Abdomen*.** Male sternum A8 posterior margin bilobed.

***Male genitalia*** (*N* = 2) (Fig. 78). Uncus long and slender, gently tapering toward apex, narrowed at apical 1/4, apex spatulate. Gnathos projection slender, ca. 2/3 of uncus length, apex blunt. Valva ventral margin straight, bent dorsad on distal 1/3, dorsal margin convex, apex rounded. Juxta with base quadrangular, with straight lateral margin, apex blunt. Saccus triangular, conspicuously pointing dorsad.

***Female genitalia*** (*N* = 3) (Fig. 118). Anterior apophyses with dorsal tip at basal 1/3. Antrum sclerotisation short, ca. as long as broad. Ductus bursae long, broad, with one loop. Corpus bursae small, globular, reticulated, with roughly defined sclerotisation between thorn and corpus opening. Thorn plump, straight, with small dents pointing toward thorn base, basally with small outwardly projected extension.

##### Distribution.

Known from the slopes of Mount Agtuuganon (1,660 m) on Mindanao Island (Philippines), at an altitude of 1,050 m.

##### Phylogenetic relationships.

See *H. mallyi* sp. nov.

##### Etymology.

Named after Mount Agtuuganon on Mindanao Island (Philippines), where the specimens were collected.

**Figures 70–75.**
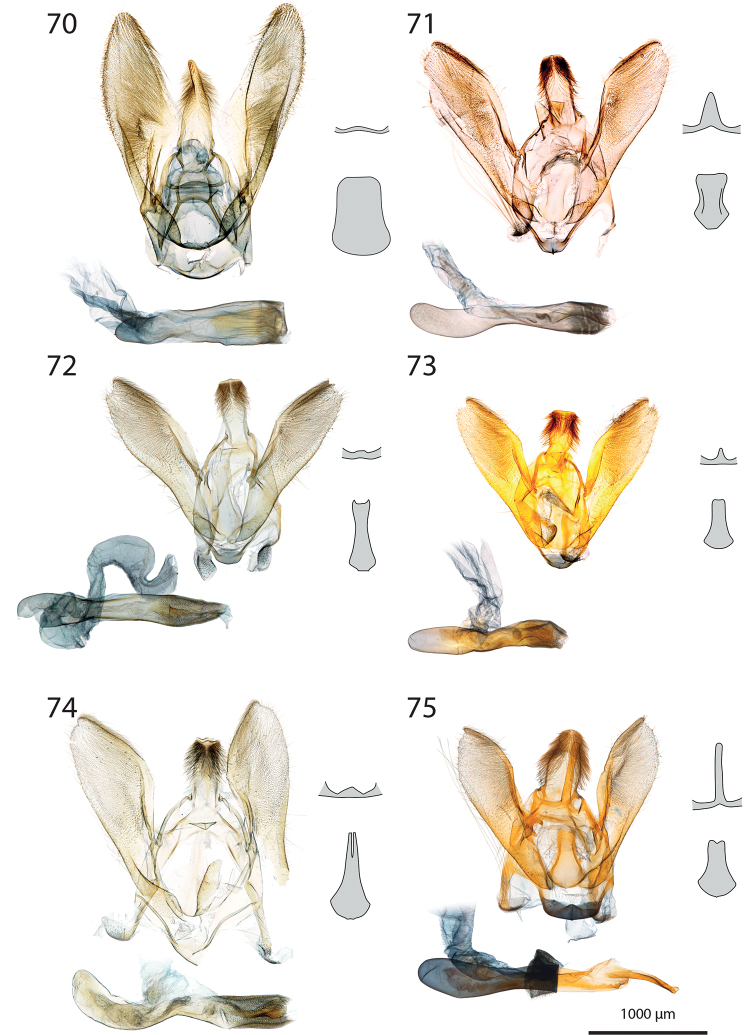
Male genitalia features of *Hoploscopa*. **70***Hoploscopa nauticorum*, TL725 ♂ **71***Hoploscopa anacantha* sp. nov., holotype, TL759 ♂ **72***Hoploscopa kelama* sp. nov., paratype, TL511 ♂ **73***Hoploscopa ignitamaculae* sp. nov., paratype, TL545 ♂ **74***Hoploscopa albomaculata* sp. nov., paratype, TL363 ♂ **75***Hoploscopa sumatrensis* sp. nov., paratype, TL538 ♂.

#### Hoploscopa boleta

Taxon classificationAnimaliaLepidopteraCrambidae

Léger & Nuss sp. nov.

97E5D53C-F33B-507E-BABA-20E71E1E28E0

http://zoobank.org/D717911D-F973-4401-BC2B-873E5ACED283

Figs 40 , 79 , 119

##### Material examined.

***Holotype***: ♂, with labels: “Col[lection] BECKER | PNG 1562”, “ Papua New Guinea | Morobe, M[oun]t Kaindi | 2360m 3.x.1992 | V. O. Becker Col[lection]; “ DNA voucher | Lepidoptera | MTD2016 | [vertically written:] no. 3160”; “TL440 | ♂”. Deposited in USNM.

***Paratypes***: 2 ♀. Papua New Guinea: 1 ♀ (DNA voucher MTD LEP3168, genitalia on slide TL448 ♀), same data as holotype; 1 ♀ (DNA voucher MTD LEP3159), same locality as holotype, 27–28.vii.1983, leg. S.E. & P. M. Miller (USNM).

##### Diagnosis.

The forewings of *H. boleta* sp. nov. display median discoidal stigma and postmedian patch of a lighter brown, the latter with a well-marked pale yellow blotch at costa. In male genitalia, the conspicuous basal lateral projections of the juxta resemble the shape of a mushroom. Female genitalia are somewhat similar to those of other *Hoploscopa* species, e.g., *H. parvimacula* sp. nov., and *H. danaoensis* sp. nov., with a short straight ductus bursae, a pear-shaped corpus bursae, and a small straight thorn.

##### Similar species.

*Hoploscopa pseudometacrossa* sp. nov., to a lesser extent *H. kelama* sp. nov. (q.v.). In *H. pseudometacrossa* sp. nov., forewing median discoidal stigma and postmedian patch are barely marked, and postmedian pale yellow blotch at costa is much smaller. In male genitalia, gnathos of *H. pseudometacrossa* sp. nov. displays a thumb-like projection, juxta shows less prominent lateral projections and an indented apex. In female genitalia, corpus bursae is larger than that of *H. boleta* sp. nov.

##### Description.

***Head*.** Antenna dorsally striped with ochreous and bronze scales. Proboscis pale yellow, basally brown. Maxillary palpi brown, base and inner side pale yellow. Labial palpi brown, ventro-basally pale yellow.

***Thorax*** (Fig. 40). Collar pale yellow. Forewing length: 9–10 mm (♂ & ♀); forewing ground colour brown; basal dash dark brown, distally pale yellow; basal and distal discoidal patches quadrangular, dark brown, basally and distally edged with pale yellow; median discoidal stigma there between trapezoid, light brown; dorsal median patch broad, pale yellow; postmedian patch light brown, with pale yellow blotch at costa; postmedian area suffused with pale yellow near costa; subterminal line dark brown, diffuse; fringe brown, with pale yellow dots. Hindwing pale yellow, darker at apex. Forelegs brown, tarsi bronze. Mid- and hindlegs with femur brown, tibia pale yellow, speckled with brown, tarsi bronze.

***Abdomen*.** Male sternum A8 posterior margin broadly indented.

***Male genitalia*** (*N* = 1) (Fig. 79). Uncus slender, conspicuously narrowed at apical 1/4, apex duck beak-shaped, bearing thick setae, ventrally with three small ridges. Gnathos projection ca. 1/3 of the uncus length, gently narrowing toward apex, apex tongue-shaped. Valva ventral margin nearly straight, dorsal margin slightly convex, apex slightly blunt. Juxta mushroom-shaped, with base rounded, laterally with two conspicuous projections, abruptly narrowed at basal 1/3, apex spatulate. Saccus small, pointing dorsad.

***Female genitalia*** (*N* = 2) (Fig. 119). Anterior apophyses with dorsal bump at posterior 1/3. Antrum sclerotisation twice as long as broad. Ductus bursae short, nearly straight. Corpus bursae globular, posterior half reticulated, medially with erect acanthae, anterior half membranous, with sclerotisation between thorn and corpus opening, medially with a sclerotised band. Thorn straight, with small dents pointing toward thorn base.

##### Distribution.

Known from Mount Kaindi in the Morobe Province (Papua New Guinea), at an altitude of 2,360 m.

##### Phylogenetic relationships.

This species displays an uncus apex similar to those of *H. jubata* sp. nov., *H. metacrossa*, and *H. pseudometacrossa* sp. nov. and is possibly related to them.

##### Etymology.

From the Latin *boletus*, in reference to the mushroom shape of the juxta.

**Figures 76–81.**
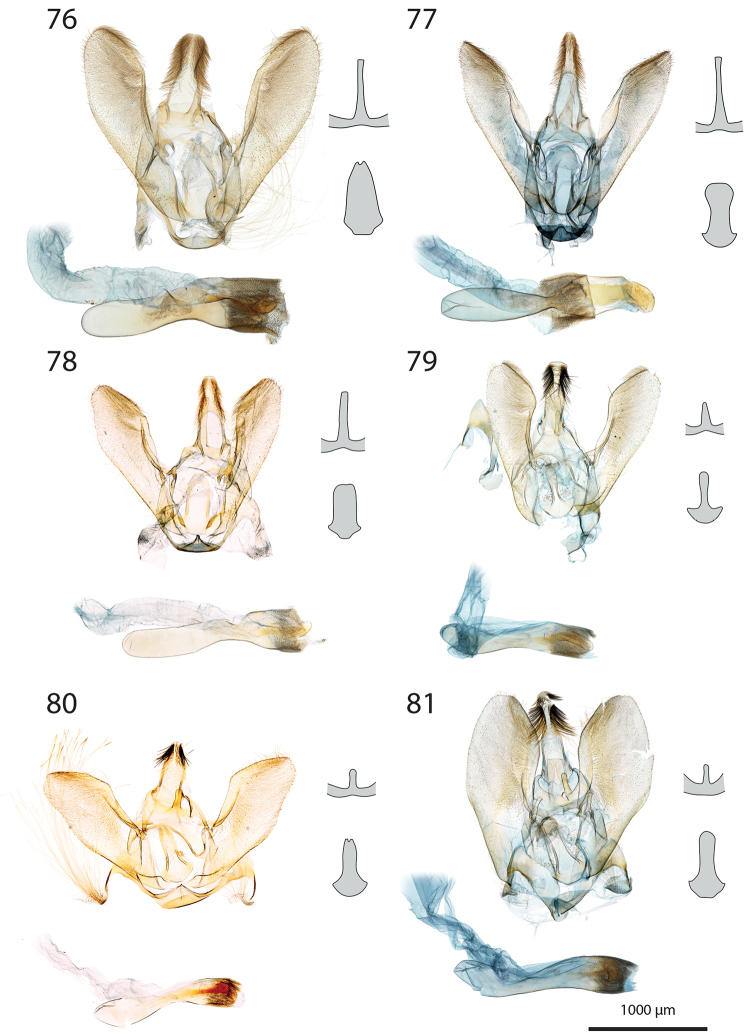
Male genitalia features of *Hoploscopa*. **76***Hoploscopa mallyi* sp. nov., paratype, TL339 ♂ **77***Hoploscopa gracilis* sp. nov., paratype, TL539 ♂ **78***Hoploscopa agtuuganonensis* sp. nov., paratype, TL675 ♂ **79***Hoploscopa boleta* sp. nov., holotype, TL440 ♂ **80***Hoploscopa pseudometacrossa* sp. nov., paratype, TL441 ♂ (uncus apex missing) **81***Hoploscopa metacrossa*, TL443 ♂.

#### Hoploscopa pseudometacrossa

Taxon classificationAnimaliaLepidopteraCrambidae

Léger & Nuss sp. nov.

FF5566C9-BDAA-55E9-BE38-EB600D4BC284

http://zoobank.org/E21452AB-8784-4A67-B270-1802A4D7293A

Figs 41 , 80 , 120

##### Material examined.

***Holotype***: ♀, with labels: “Papua New Guinea: W[estern]. H[ig]hl[and] | Pr[o]v[ince], n[ea]r Mt. Hagen, Kuk Ag | Res[earch]. Sta[tion]., 1600m, UV Lite | 19–20 August 1983 | Scott E. & Pamela Miller”; “DNA voucher | Lepidoptera | date: i.2018 | MTD 7898 | [vertically written:] DNA-voucher”; “TL689 | ♀”. Deposited in USNM.

***Paratypes***: 1 ♂, 1 ♀. Papua New Guinea: 1 ♂, 1 ♀ (♂ with DNA voucher MTD LEP3161 & genitalia on slide TL441 ♂; ♀ with DNA voucher MTD LEP3155 & genitalia on slide TL435 ♀), same data as holotype (USNM).

##### Diagnosis.

The forewings of *H. pseudometacrossa* sp. nov. display barely marked median discoidal stigma and postmedian patch, while basal and distal discoidal patches are dark brown. Male hindwing displays an androconial organ on the dorsum. In male genitalia, the gnathos shows a short, thumb-like projection, and the juxta is slender, with notched apex. In female genitalia, the ductus bursae is relatively short-sized, the corpus bursae is large, spherical, with a small straight thorn.

##### Similar species.

*Hoploscopa boleta* sp. nov. (q.v.), *H. metacrossa*, *H. kelama* sp. nov. (q.v.). Forewing median discoidal stigma forms a pale yellow Y with median cubital patch in *H. metacrossa*. In male genitalia, gnathos projection of *H. metacrossa* is thinner, valva is larger, and juxta apex is rounded. Female genitalia are very similar, but the antrum sclerotisation is shorter, and the corpus bursae smaller in *H. metacrossa*.

##### Description.

***Head*.** Antennae dorsally with bronze to brown scales. Proboscis pale yellow to brown. Maxillary palpi brown, base and inner side pale yellow. Labial palpi brown, ventro-basally pale yellow.

***Thorax*** (Fig. 41). Collar pale yellow. Forewing length: 9–10 mm (♂ & ♀); forewing ground colour brown; basal dash dark brown, distally pale yellow; basal and distal discoidal stigma quadrangular, dark brown; median discoidal stigma faded, basally and distally thinly edged with pale yellow; postmedian patch faded, with distal edge dark brown, at costa with pale yellow blotch; postmedian line marked on costal half; postmedian area suffused with pale yellow near costa; subterminal line pale yellow; fringe brown, with pale yellow dots. Hindwing pale brown; in males, presence of an androconial organ on the dorsum of the hindwing. Forelegs brown. Mid- and hindlegs with femur brown, tibia brown speckled with pale yellow, tarsi bronze.

***Abdomen*.** Male sternum A8 posterior margin broadly indented, with short, rounded lateral projections.

***Male genitalia*** (*N* = 1) (Fig. 80). Uncus slender, narrowed on apical 1/4 which bears thick setae, apex missing on slide examinated. Gnathos projection thumb-shaped, ca. 1/4 of uncus length. Valva ventral margin bent dorsad on distal half, dorsal margin medially angled, apex roughly rounded. Juxta with base rounded, medially narrowed, apex notched. Saccus broad, triangular, pointing dorsad.

***Female genitalia*** (*N* = 2) (Fig. 120). Anterior apophyses with dorsal bump at posterior 1/3. Antrum sclerotisation twice as long as broad. Ductus bursae short, more or less straight, bent before corpus opening. Corpus bursae large, posterior half reticulated, medially covered with erect acanthae, anterior half membranous, with weak sclerotisation at thorn base. Thorn straight, with small dents pointing toward thorn base, basally with small outwardly projected extension.

##### Distribution.

Known from Mount Hagen in the Morobe Province (Papua New Guinea), at an altitude of 1,600 m.

##### Phylogenetic relationships.

The hindwing scent scales observed in males of *H. jubata* sp. nov., *H. pseudometacrossa* sp. nov., and *H. metacrossa* suggest a close relationship between these three species.

##### Etymology.

The name is made by the apposition of the prefix *pseudo*- from the Greek *pseudes*, false and *metacrossa*, referring to the resemblance of this species with *H. metacrossa*.

**Figures 82–86.**
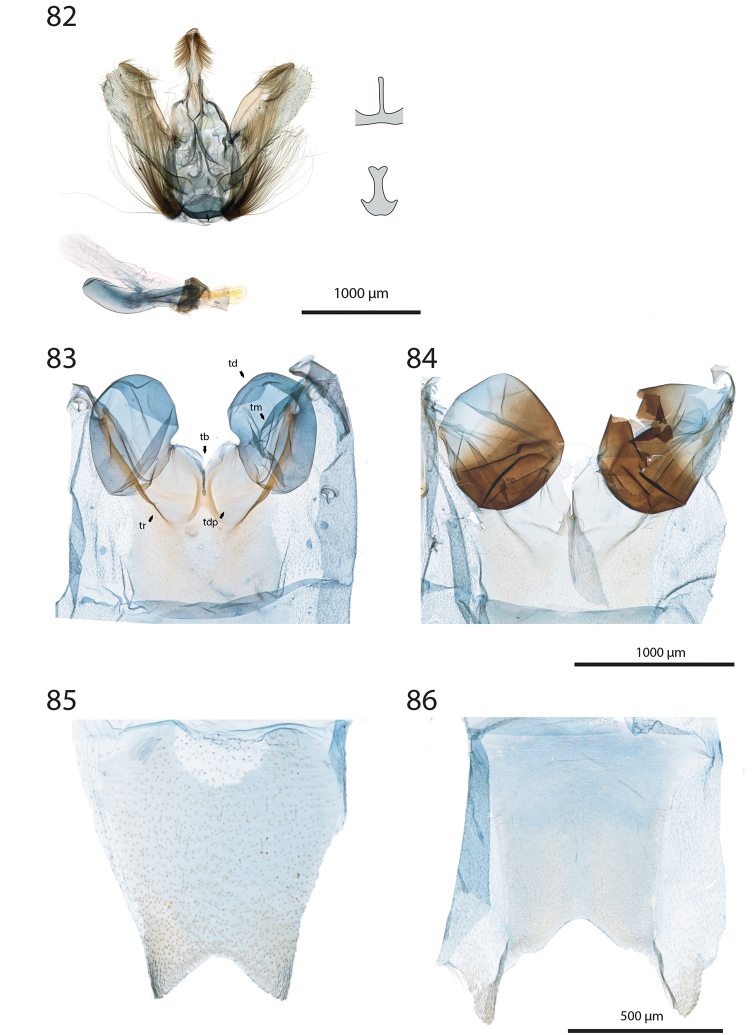
Male genitalia and abdomen features of *Hoploscopa*. **82***Hoploscopa jubata* sp. nov., paratype, TL542 ♂ **83** Tympanal organs of *Hoploscopa sumatrensis* sp. nov., paratype, TL538 ♂ **84** Male tympanal organs of *Hoploscopa metacrossa*, TL472 ♂ **85** Sternite VIII of Hoploscopa sp. near isarogensis, TL626 ♂ **86** Sternite VIII of *Hoploscopa metacrossa*, TL443 ♂. Abbreviations: tb (tympanic bridge), td (tympanic drum), tdp (tympanic depression), tm (tympanum), tr (transverse ridge).

#### Hoploscopa metacrossa

Taxon classificationAnimaliaLepidopteraCrambidae

(Hampson, 1917)

1B9223FE-1873-5141-B0C5-A32F4C91B1FD

Figs 42 , 45 , 81 , 84 , 86 , 121

##### Material examined.

***Holotype***: ♂, with labels: “Holo- | type” [round label, red ringed]; “Fak-Fak | Dutch New Guinea | Dec’[19]07 | 1700f[ee]t | (Pratt)”; “1913-216”; “Scoparia | metacrossa | type ♂. H[a]mps[o]n.” [handwritten]; “Pyralidae | Brit[ish].Mus[eum]. | Slide N°. | 3612”; "NHMUK 010923297” [barcode appended]. Deposited in NHMUK.

##### Other specimens examined.

12 ♂, 38 ♀. Papua New Guinea: 10 ♂ (3 with DNA vouchers MTD7899, MTD7900, MTD7901 and genitalia on slides TL690 ♂, TL691 ♂, TL692 ♂ respectively; 1 with genitalia on slide TL482 ♂), 25 ♀ (1 with genitalia on slide TL481 ♀), Morobe Province, near Bulolo, Mt Susu National Reserve, *Araucaria* forest, 975m, 27–28.viii.1983, leg. S. Miller; 1 ♂ (genitalia on slide TL472 ♂), 9 ♀ (2 with DNA vouchers MTD LEP3166, MTD LEP3167 and genitalia on slides TL446 ♀, TL447 ♀), Morobe Province, Wau, Wau Ecological Institute, montane forest, 1200 m, 12–24.vii.1983 (2 ♀), 25–31.vii.1983 (1 ♂, 3 ♀), 1–10.viii.1983 (1 ♀), 23–31.viii.1983 (3 ♀), leg. S. E. & P. M. Miller; 4 ♀, same collecting data except “trap at zoo pond”, 25–27.vii.1983; 1 ♂ (DNA voucher MTD LEP3163 & genitalia on slide TL443 ♂), Morobe Province, Wau, 1200 m, 8–14.xii.1976, leg. G. F. Hevel & R. E. Dietz (USNM).

##### Diagnosis.

The forewings of *H. metacrossa* display Y-shaped median and a postmedian pale yellow patches observed in several other *Hoploscopa* species (e.g., *H. danaoensis* sp. nov.). Male hindwing displays an androconial organ on the dorsum. The strongly sclerotised tympanic drum in tympanal organs of males is only observed in this species. In male genitalia, gnathos is projected into a small, slender, tongue-shaped tip, and valva is broad, with a rounded ventral margin. In female genitalia, antrum sclerotisation is as long as wide, ductus bursae is short, straight and the corpus bursae is large.

##### Similar species.

*Hoploscopa brunnealis*, *H. danaoensis* sp. nov., *H. kinabaluensis* sp. nov. (q.v.), *H. pseudometacrossa* sp. nov. (q.v.). Males of *H. metacrossa* are easily separated from similar species (except *H. pseudometacrossa* sp. nov.) by the presence of scent organs on the hindwing, the sclerotised tympanic drum, and the characteristic spatulate uncus apex with ventral ridges. Female genitalia of the four above-mentioned species are similar, but ductus bursae is bent before corpus bursae in these species (nearly straight in *H. metacrossa*) and corpus bursae are smaller than that of *H. metacrossa*.

##### Description.

***Head*.** Antennae dorsally brown. Proboscis pale yellow to brown. Maxillary palpi brown, base and inner side pale yellow. Labial palpi brown, ventral base and inner side pale yellow.

***Thorax*** (Fig. 42, 45). Collar pale yellow. Forewing length: 9–10 mm (♂ & ♀); forewing ground colour brown; basal dark brown spot distally pale yellow; median discoidal stigma trapezoid, pale yellow, together with cubital pale yellow patches forming a Y; postmedian patch pale yellow speckled with brown, distal edge dark brown; postmedian line marked on costal half; postmedian area variously suffused with pale yellow; subterminal line pale yellow; fringes brown with pale yellow dots. Hindwing pale brown; in males, presence of an androconial organ on the dorsum of the hindwing. Forelegs brown. Mid- and hindlegs with femur brown; tibia pale yellow, speckled with brown; tarsi brown.

***Abdomen*** (Fig. 84, 86). Male sternum A8 posterior margin broadly indented, with short, rounded lateral projections.

***Male genitalia*** (*N* = 3) (Fig. 81). Uncus slender, narrowed at apical 1/4, apex spatulate, bearing thick setae, ventrally with five small ridges. Gnathos projection ca. 1/4 of uncus length, slender, tongue-shaped. Valva ventral margin curved dorsad on distal half, dorsal margin conspicuously convex, apex rounded. Juxta with base rounded or slightly quadrangular, narrowing at basal 1/4, apex tongue-shaped. Saccus triangular, conspicuously pointing dorsad. Phallus with elongated, flat, spatula-shaped cornutus with subapical tip.

***Female genitalia*** (*N* = 3) (Fig. 121). Anterior apophyses with dorsal bump at posterior 1/3. Antrum sclerotisation short, as long as wide. Ductus bursae short, nearly straight. Corpus bursae spherical, reticulated, with sclerotisation between thorn and corpus opening and faintly marked sclerotisation medially. Thorn straight, with small dents pointing toward thorn base, basally with small outwardly projected extension.

##### Distribution.

Known from the Papua (Indonesia) and the Morobe Provinces (Papua New Guinea) in New Guinea, at altitudes between 600 and 1,200 m.

##### Phylogenetic relationships.

See *H. boleta* sp. nov. and *H. pseudometacrossa* sp. nov.

##### Remarks.

Nuss (1998) transferred this species from *Eudorina* to *Hoploscopa*.

**Figures 87–90.**
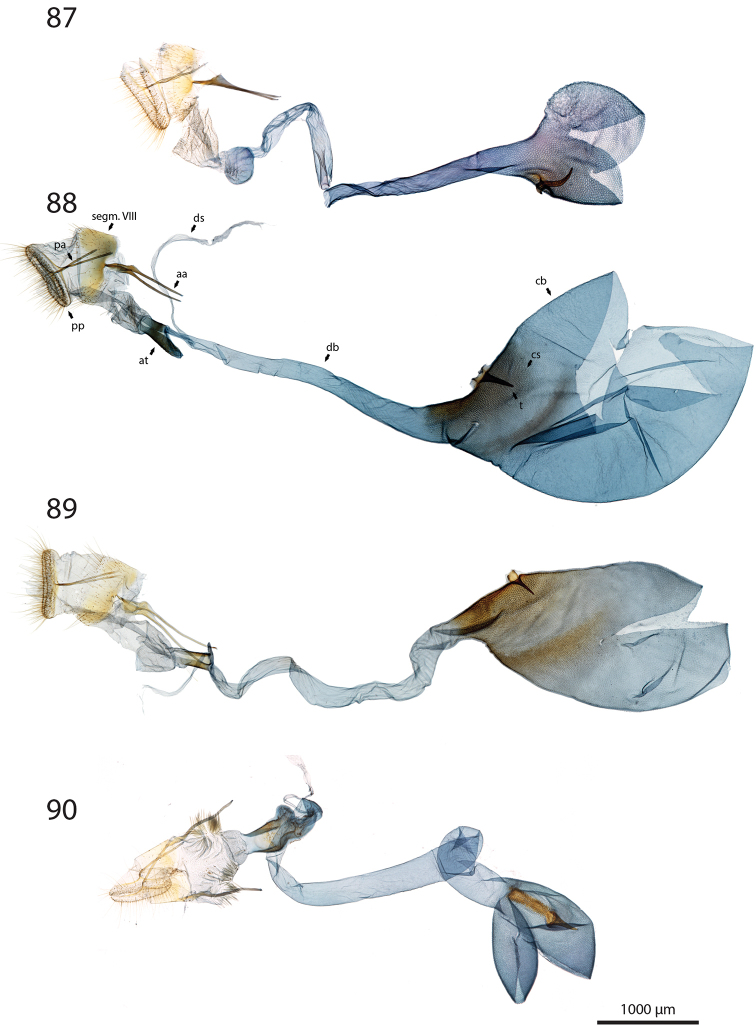
Female genitalia features of *Hoploscopa*. **87***Hoploscopa albipuncta* sp. nov., paratype, TL609 ♀ **88***Hoploscopa matheae* sp. nov., paratype, TL599 ♀ **89***Hoploscopa sepanggi* sp. nov., paratype, TL547 ♀ **90***Hoploscopa cynodonta* sp. nov., paratype, TL625 ♀. Abbreviations: aa (anterior apophyses), at (antrum), cb (corpus bursae), cs (corpus sclerotization), db (ductus bursae), ds (ductus seminalis), pa (posterior apophyses), pp (papillae anales), segm. VIII (segment VIII), t (thorn).

#### Hoploscopa jubata

Taxon classificationAnimaliaLepidopteraCrambidae

Léger & Nuss sp. nov.

006C0C0D-B5CB-54BB-AC61-F308D63360E5

http://zoobank.org/F81F0837-5324-4B81-AC72-B0519F850301

Figs 2 , 43 , 44 , 82 , 122

##### Material examined.

***Holotype***: ♂, with labels: “Papua New Guinea | Morobe Prov[ince]., n[ea]r Bulolo | Mt Susu Nat[ional]. Res[erve]., 975m | 27–28 Aug.1983, S. Miller | UV Lite, Araucaria For[est].”; “ DNA voucher | Lepidoptera | MTD2016 | [vertically written:] no. 3164”; “TL444 | ♂”. Deposited in USNM.

***Paratype*s**: 4 ♂, 24 ♀. Papua New Guinea. 4 ♂ (1 with genitalia on slide TL447 ♂, 1 with genitalia on slide TL542 ♂, 1 with wing preparation TL706), 21 ♀ (1 with DNA voucher MTD LEP3165 & genitalia on slide TL445 ♂, 2 with genitalia on slide TL476 ♀ & TL541 ♀), same data as holotype. 1 ♀, Morobe Province, Wau, Wau Ecological Institute, 12–24.vii.1983; 1 ♀, same data except 1–10.viii.1983; 1 ♀, Morobe Province, Wau, 1200 m, 8–14.xii.1976, at black light, leg. G. F. Hevel & R. E. Dietz (USNM).

##### Diagnosis.

This species shows a strong sexual dimorphism. In males, median and postmedian markings are white, and postmedian area is broadly suffused with white. In females, basal patch, median discoidal stigma and postmedian patch are reddish orange, and subterminal area is orange. Male hindwing displays an androconial organ on the dorsum. In male genitalia, gnathos projection is slender, ca. 1/3 of the uncus length, and juxta displays two conspicuous lateral projections at base, and a deeply indented apex. In female genitalia, the four loops formed by the ductus bursae is unique to this species.

##### Similar species.

No similar species known.

##### Description.

***Head*.** Antennae dorsally brown. Proboscis brown. Maxillary palpi brown, base and inner side pale yellow. Labial palpi brown, ventral base and inner side pale yellow.

***Thorax*** (Figs 2, 43, 44). Collar brown. Forewing length: 9–10 mm (♂ & ♀); forewing ♂ ground colour brown; basal narrow white streak, distally marked with yellow and orange; median discoidal stigma white, filled with few red and yellow scales, together with cubital and dorsal patch forming a white Y; postmedian patch brown speckled with red, more or less marked, distally with costal white streak; postmedian line marked on costal half; postmedian area with broad white suffusion; subterminal line conspicuously incurved inward at CuA2, white; fringe chequered brown and pale yellow; forewing ♀ (Fig. 44) ground colour brown; yellow basal patch crossed by orange streak; median trapezoid white patch, filled to a various extend with yellow and red scales, together with cubital and dorsal white patches forming a Y; postmedian patch roughly oval, reddish orange, basally edged with yellow, distally with costal white streak; postmedian line marked on costal half; postmedian area faintly speckled with white between postmedian patch and subterminal line; subterminal line white, incurved inwards at CuA2; subterminal field marked with reddish orange and yellow; fringe brown with pale yellow dots. Hindwing pale yellow; in males, presence of an androconial organ on the dorsum of the hindwing. Forelegs bronze. Midlegs with femur brown; tibia brown, distally pale yellow; tarsi bronze. Hindlegs with femur brown; tibia pale yellow, speckled with brown; tarsi bronze.

***Abdomen*.** Male tergite I, II partially sclerotised; tergite III sclerotised, with two shallow depressions on each side of middle, bearing patches of modified scales. Sternum A8 posterior margin straight.

***Male genitalia*** (*N* = 3) (Fig. 82). Uncus long and slender, basally narrow, medially slightly widened, narrowed at apical 1/4, apex spatula-shaped, ventrally with eight small ridges. Gnathos projection slender, ca. 1/3 of the uncus length. Valva gently bending dorsad from distal 1/3, dorsal margin slightly convex, apex roughly truncate, forming a furrow ring. Juxta mushroom-shaped, with base roughly rounded, slightly concave on its middle, with two conspicuous lateral extensions pointing outward, conspicuously narrowed at basal 1/4, apex bilobed. Saccus broad, triangular, pointing dorsad.

***Female genitalia*** (*N* = 3) (Fig. 122). Anterior apophyses with dorsal bump at posterior 1/3. Antrum sclerotisation short. Ductus bursae very long, forming four conspicuous loops bent before corpus opening. Corpus bursae small, globular, reticulated, medially with light broad sclerotised patch, with weak rounded sclerotisation at corpus opening. Thorn gently curved, with small dents pointing toward thorn base, basally with small outwardly projected extension.

##### Distribution.

Known from Wau and from Mount Susu in the Morobe Province (Papua New Guinea), at altitudes between 950 m and 1,200 m.

##### Phylogenetic relationships.

See *H. boleta* sp. nov. and *H. pseudometacrossa* sp. nov.

##### Etymology.

From the Latin *jubatus*, having a mane, referring to the dense hair covering on the inner side of the valva in male genitalia.

##### Remarks.

This species is the only one known from the genus to exhibit a pronounced sexual dimorphism.

**Figures 91–94.**
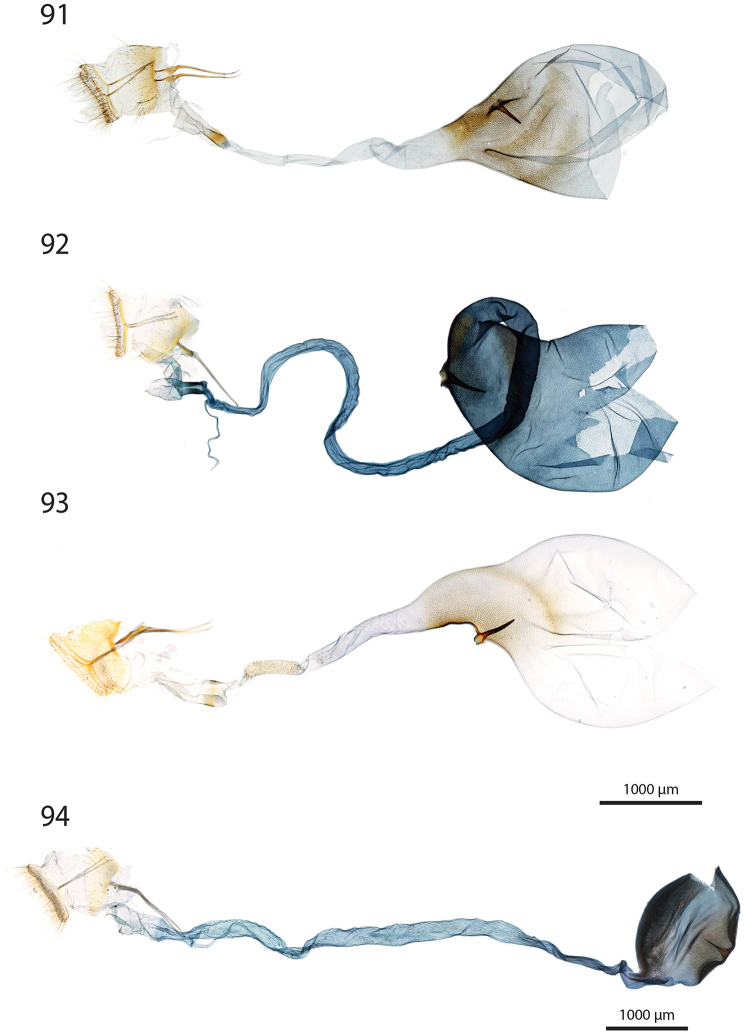
Female genitalia features of *Hoploscopa*. **91***Hoploscopa parvimacula* sp. nov., paratype, TL551 ♀ **92***Hoploscopa kinabaluensis* sp. nov., TL674 ♀ **93***Hoploscopa luteomacula*, paratype, GU prep. Nuss 743 ♀ **94***Hoploscopa obliqua*, paratype, TL656 ♀.

### Misplaced species

*Hoploscopa mediobrunnea* (De Joannis, 1929) from Vietnam has been provisionally placed in *Hoploscopa* by Nuss (1998). He justified his choice as follows: ““Since *Eudorina* Snellen is a homonym of *Eudorina* Ehrenberg, 1832 (Protozoa), I place *E. mediobrunnea* here preliminarily in *Hoploscopa*. With the white discocellular stigma and the white fasciata adjacent to the median space, the wing pattern of this species look similar to the Musotiminae genus *Uthinia*. In contrast to *Uthinia*, *H. mediobrunnea* shows porrect labial palpi, a long ductus bursae, and the ductus seminalis originates near the antrum. The corpus bursae shows three round signa, with inwardly directed tiny spines arising from their edges.” (Nuss 1998). Its wing shape and the female genitalia are different from all *Hoploscopa* species we have examined to date, and we therefore assume that *H. mediobrunnea* belongs to a different genus.

**Figures 95–98.**
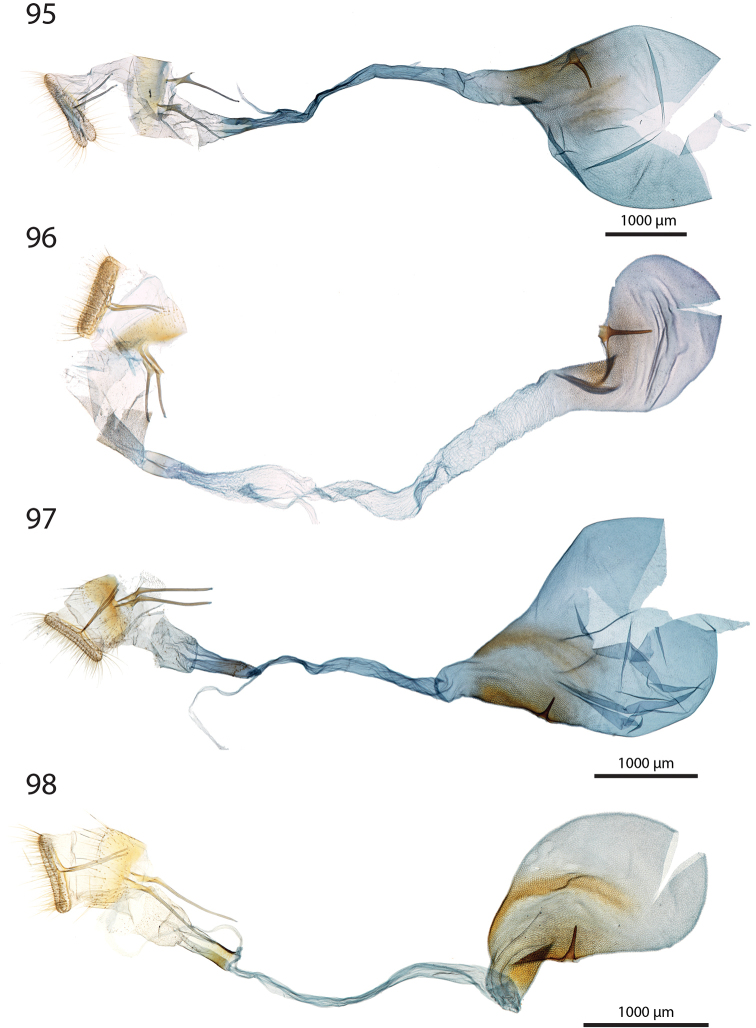
Female genitalia features of *Hoploscopa*. **95***Hoploscopa marijoweissae* sp. nov., paratype, TL709 ♀ **96***Hoploscopa gombongi* sp. nov., paratype, TL654 ♀ **97***Hoploscopa pangrangoensis* sp. nov., paratype, TL627 ♀ **98***Hoploscopa ypsilon* sp. nov., paratype, TL620 ♀.

## Discussion

Our iterative taxonomic revision resulted in the discovery and description of 26 new species as well as the redescription of 15 species. Thirty of 38 morphospecies were concordant with species delimitation based on the COI barcode. Distinct genetic divergence (i.e., more than 2%) among populations of different islands, with minor morphological differences in wing pattern or male genitalia, suggests reproductive isolation through allopatric effects. In *H. matheae* sp. nov. and *H. parvimacula* sp. nov., specimens from Borneo and from Malaysia Peninsula form two distinct MOTUs that deserve closer examination. Cryptic divergence between Borneo and Malayan Peninsula has been found in other studies, e.g., ants (Quek et al. 2007, Feldhaar et al. 2010), bats (Francis et al. 2010), and birds (Lohman et al. 2011). Similarly, specimens of *H. danaoensis* sp. nov. and *H. isarogensis* sp. nov. from different Philippines islands display distinct genetic differentiation suggesting allopatric differentiation. Cryptic diversity among populations of different Philippines islands has also been reported in birds (Lohman et al. 2010). We found specimens from Eastern New Guinea resembling the type specimens of *H. quadripuncta* and *H. semifascia* from Western New Guinea, but displaying minor differences in male genitalia in each case. This suggests potential east-west differentiation along the Cordillera, as observed in birds (Joseph et al. 2001). Molecular investigations of specimens from Western New Guinea should be performed to test this hypothesis. High DNA barcode divergence in geographically distant species reveals the need for a better sampling with higher geographical coverage to investigate both intraspecific and interspecific variation (Bergsten et al. 2012, Talavera et al. 2013).

**Figures 99–102.**
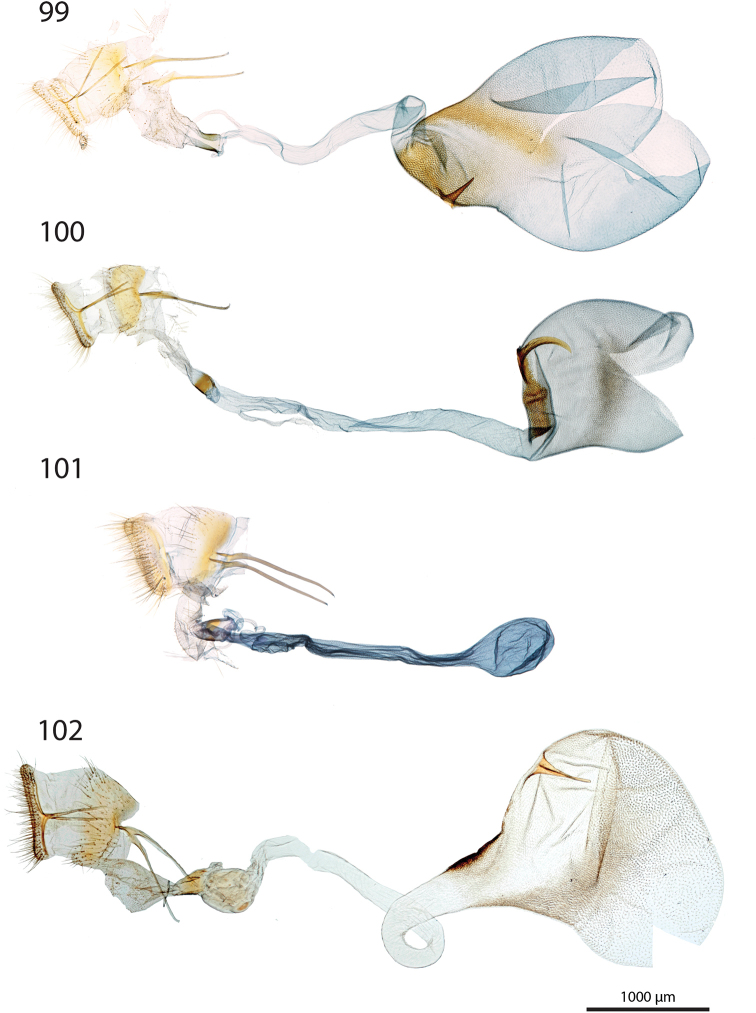
Female genitalia features of *Hoploscopa*. **99***Hoploscopa danaoensis* sp. nov., paratype, TL618 ♀ **100***Hoploscopa isarogensis* sp. nov., holotype, TL514 ♀ **101***Hoploscopa tonsepi* sp. nov., paratype, TL658 ♀ **102***Hoploscopa aurantiacalis*, lectotype, Pyralidae Brit. Mus. Slide N° BMNH20246 ♀.

Two cases of high genetic divergence found in sympatric populations deserve closer attention. The first case involves *H. sumatrensis* sp. nov. from north Sumatra, where two mitochondrial lineages differ by 4.1–6%. In the second case, a divergence of 1.7–2.2% is observed in sympatric specimens of *H. kinabaluensis* sp. nov. on Mount Kinabalu. Closer examination of the morphology did not reveal any differences in either case. Inclusion of nuclear markers is needed in order to test if these mitochondrial lineages reflect different species or cases of introgression, symbiont infection, or maternal lineage sorting (Funk and Omland 2003, Hurst and Jiggins 2005, Harrison and Larson 2014). Notably, introgression has been reported in Lepidoptera (Zakharov et al. 2009; Cong et al. 2017), including Crambidae (Mally et al. 2018).

**Figures 103–106.**
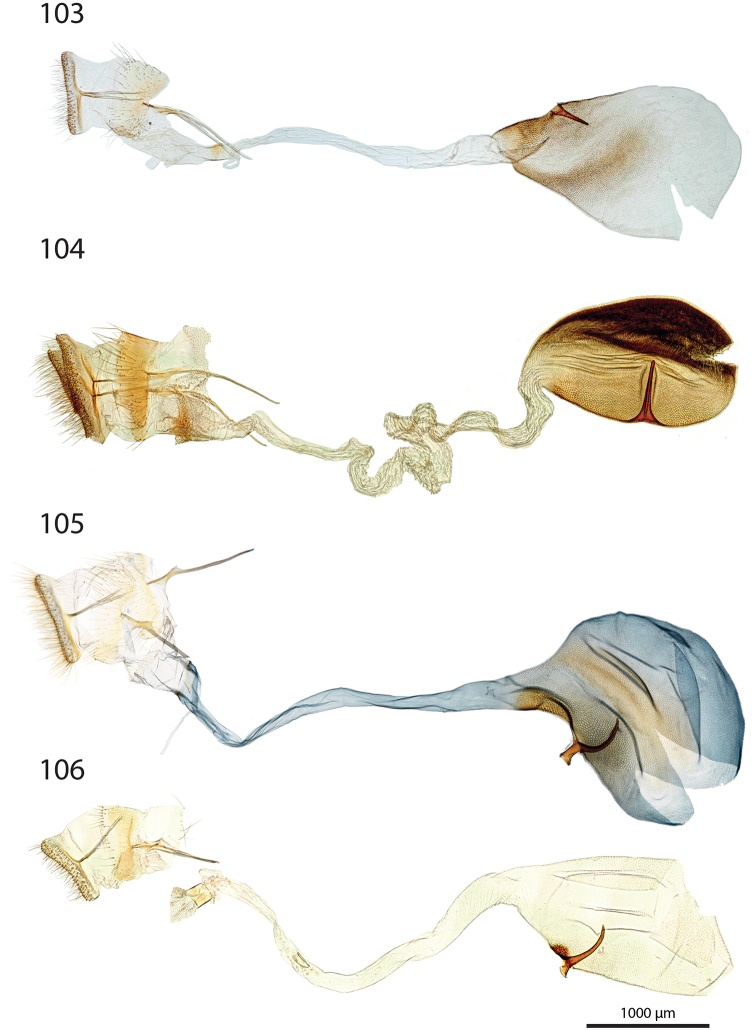
Female genitalia features of *Hoploscopa*. **103***Hoploscopa brunnealis*, lectotype, Pyralidae Brit. Mus. Slide N° BMNH20247 ♀ **104***Hoploscopa ocellata*, holotype, Pyralidae Brit. Mus. Slide N° BMNH20258 ♀ **105***Hoploscopa quadripuncta*, TL713 ♀ **106***Hoploscopa semifascia*, holotype, Pyralidae Brit. Mus. Slide N° BMNH20253 ♀.

The investigated material more than doubled the number of *Hoploscopa* species. With 41 species now described, we still have not reached the saturation phase in species discovery for this group. For example, morphology of the female genitalia and molecular data support specimens MTD8238 and MTD8243 as different species, for which male specimens remain to be discovered. Similarly, molecular data and images of the habitus support specimen USNM_ENT_00739239 as a new but yet undescribed species. Unfortunately, this specimen could not be located (Scott Miller, pers. comm.). Additionally, the nine cases of morphospecies split into two or more MOTUs suggest putative new cryptic species and require further examination. In the NHMUK collection, we estimate at least a further 30 species await description. The presumed sister-group *Perimeceta* is probably less diverse, with four described species to date and an estimated twelve undescribed species in the collections of the NHMUK.

**Figures 107–110.**
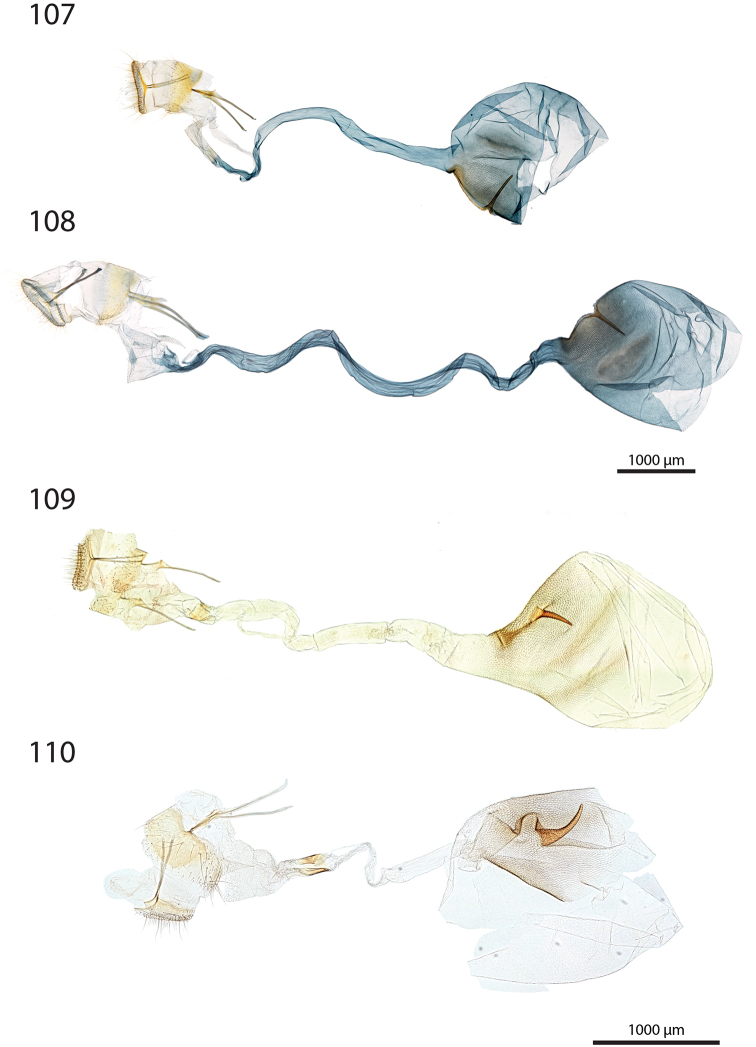
Female genitalia features of *Hoploscopa*. **107***Hoploscopa astrapias*, TL23 ♀ **108***Hoploscopa anamesa*, TL718 ♀ **109***Hoploscopa subvariegata*, lectotype, Pyralidae Brit. Mus. Slide N° BMNH20254 ♀ **110***Hoploscopa triangulifera*, holotype, Pyralidae Brit. Mus. Slide N° BMNH20256 ♀.

*Hoploscopa* is predominantly found in montane wet forests of South-East Asia at altitudes above 1,000 m, while species from higher latitudes tend to stretch their altitudinal distribution down to the lowlands, e.g., on the Philippines (*H. danaoensis* sp. nov., *H. isarogensis* sp. nov.) and the Melanesian islands (*H. astrapias*, *H. anamesa*, *H. nauticorum*). Mountain of South-East Asia are also home to other species-rich crambid groups, e.g., *Glaucocharis*, *Micraglossa*, and *Scoparia* (Gaskin 1985, Nuss 1998). Based on our data, we hypothesise isolation through elevation, higher specialisation, and seasonality as three potential factors driving the diversification of *Hoploscopa*. Isolation through elevation promotes species diversity and endemism (Steinbauer et al. 2016). The study of Merckx et al. (2015) on a broad range of taxonomic groups from Mount Kinabalu suggested endemic species on tropical mountains to originate either from long-distance dispersal (eccentric species) or from colonisation from local lowlands (centric species). All seven new species from Mount Kinabalu described in this paper do not share morphological similarities and no close relationships was recovered among them. On the contrary, *H. mallyi* sp. nov. from Mt Kinabalu forms a well-supported clade with *H. agtuuganonensis* sp. nov. from Mindanao and *H. gracilis* sp. nov. from Sumatra and represents a possible case of long-distance dispersal across islands. Colonisation from local lowland is also conceivable in *Hoploscopa*: *H. cynodonta* sp. nov. is reported at an altitude of 300 m in Brunei, and other *Hoploscopa* specimens have been collected at altitudes of 500 m at the foot of Mount Kinabalu (Schulze 2000). Secondly, on-site specialisation on host plant is another possible driver of speciation. Moths were shown to be high specialists in tropical mountains (Rodríguez-Castañeda et al. 2010). Larvae of *Hoploscopa* feed on ferns, which show a high species diversity in montane wet forests of South-East Asia (Ebihara and Kuo 2012). Current host plant records report five *Hoploscopa* species to feed on ferns belonging to three distinct genera (Miller et al. 2015; Mally et al. 2017). Ferns are scarcely used by Lepidoptera (Weintraub et al. 1995) and thus represent a large palette of potential larval food. Finally, there is a possible seasonality occurring in *Hoploscopa* species. The *Hoploscopa* larva described by Mally et al. (2017) and collected during a field trip on Mount Kinabalu did not match any of the eight species we collected there, although we cannot rule out the possibility that we simply did not collect it. Seasonality is observed in Pyraloidea from lowlands surrounding Mount Kinabalu (Schulze and Fiedler 2003) but no studies are available on the seasonality of moths at higher elevations in South-East Asia. Further systematic work on *Hoploscopa*, phylogeographic investigations, as well as accumulation of host plant records will assist in completing a more comprehensive picture of its diversity.

**Figures 111–114.**
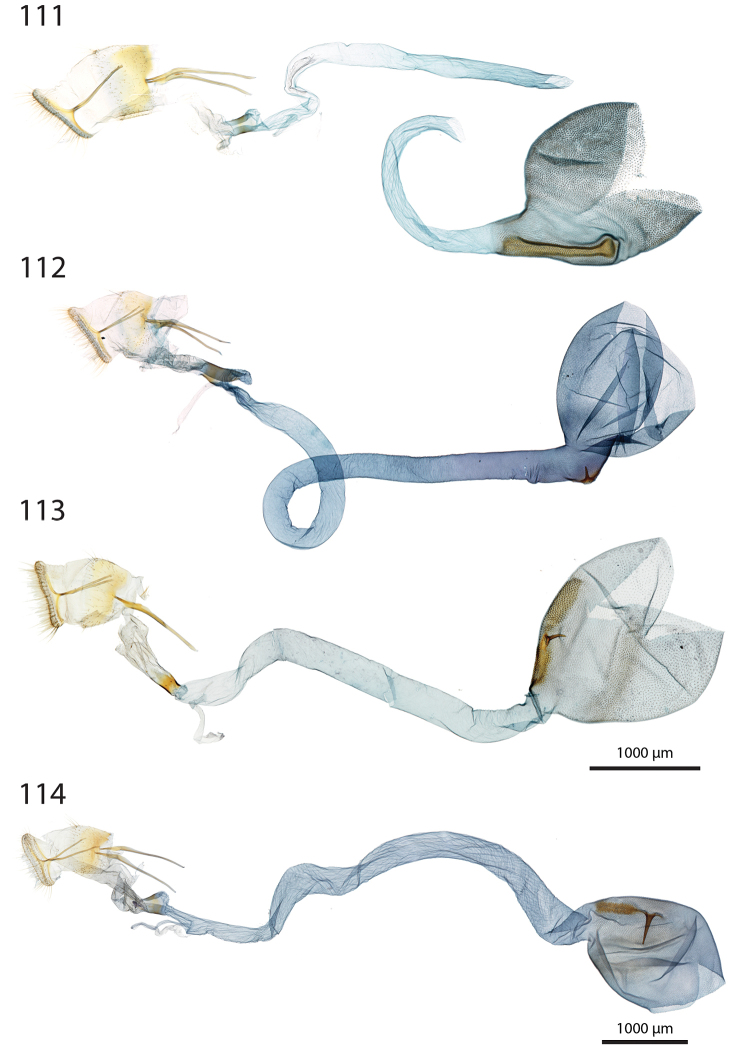
Female genitalia features of *Hoploscopa*. **111***Hoploscopa anacantha* sp. nov., paratype, TL732 ♀ **112***Hoploscopa kelama* sp. nov., paratype, TL665 ♀ **113***Hoploscopa ignitamaculae* sp. nov., paratype, TL364 ♀ **114***Hoploscopa albomaculata* sp. nov., paratype, TL529 ♀.

**Figures 115–118.**
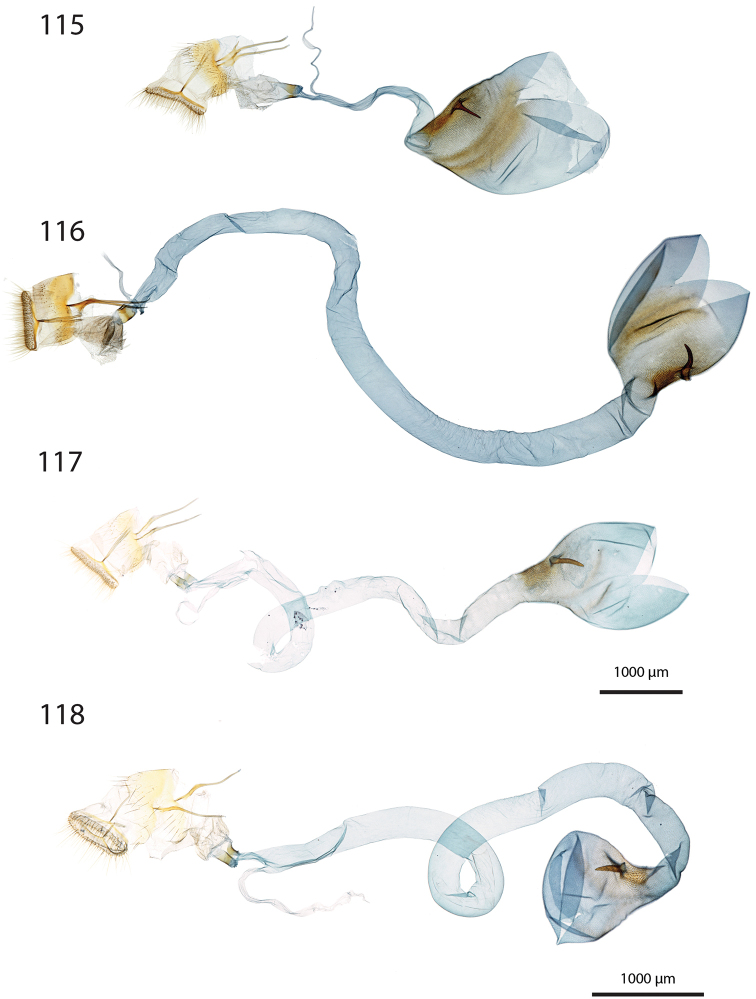
Female genitalia features of *Hoploscopa*. **115***Hoploscopa sumatrensis* sp. nov., paratype, TL530 ♀ **116***Hoploscopa mallyi* sp. nov., paratype, TL513 ♀ **117***Hoploscopa gracilis* sp. nov., paratype, TL527 ♀ **118***Hoploscopa agtuuganonensis* sp. nov., holotype, TL616 ♀.

**Figures 119–122.**
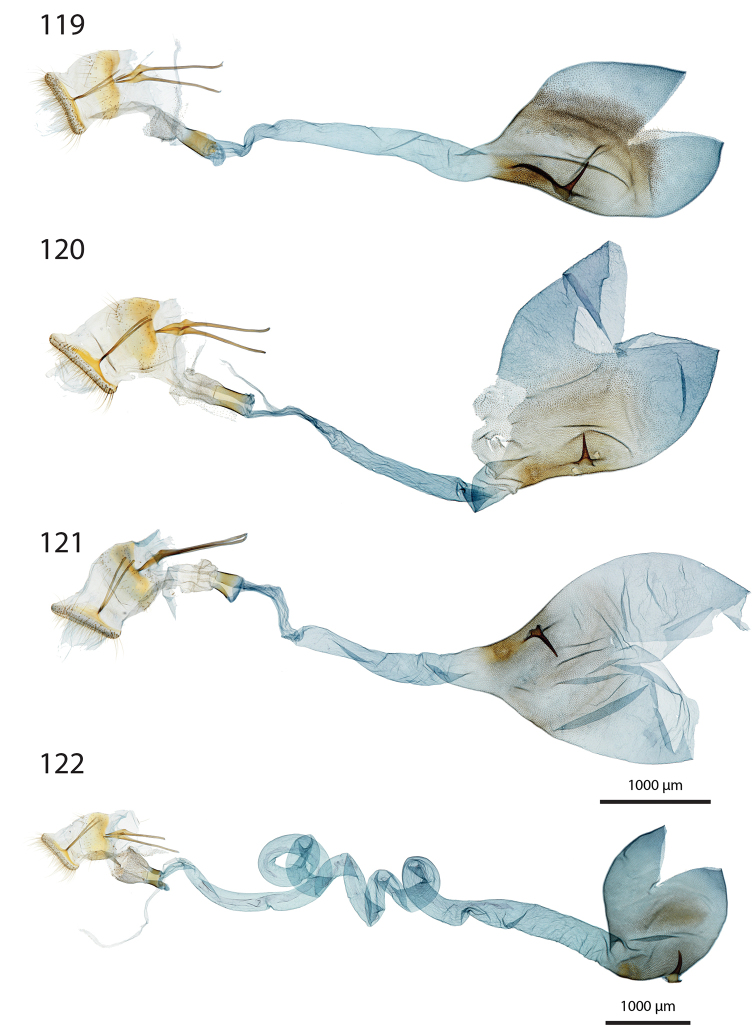
Female genitalia features of *Hoploscopa*. **119***Hoploscopa boleta* sp. nov., paratype, TL448 ♀ **120***Hoploscopa pseudometacrossa* sp. nov., paratype, TL435 ♀ **121***Hoploscopa metacrossa*, paratype, TL446 ♀ **122***Hoploscopa jubata* sp. nov., paratype, TL445 ♀.

**Figure 123.**
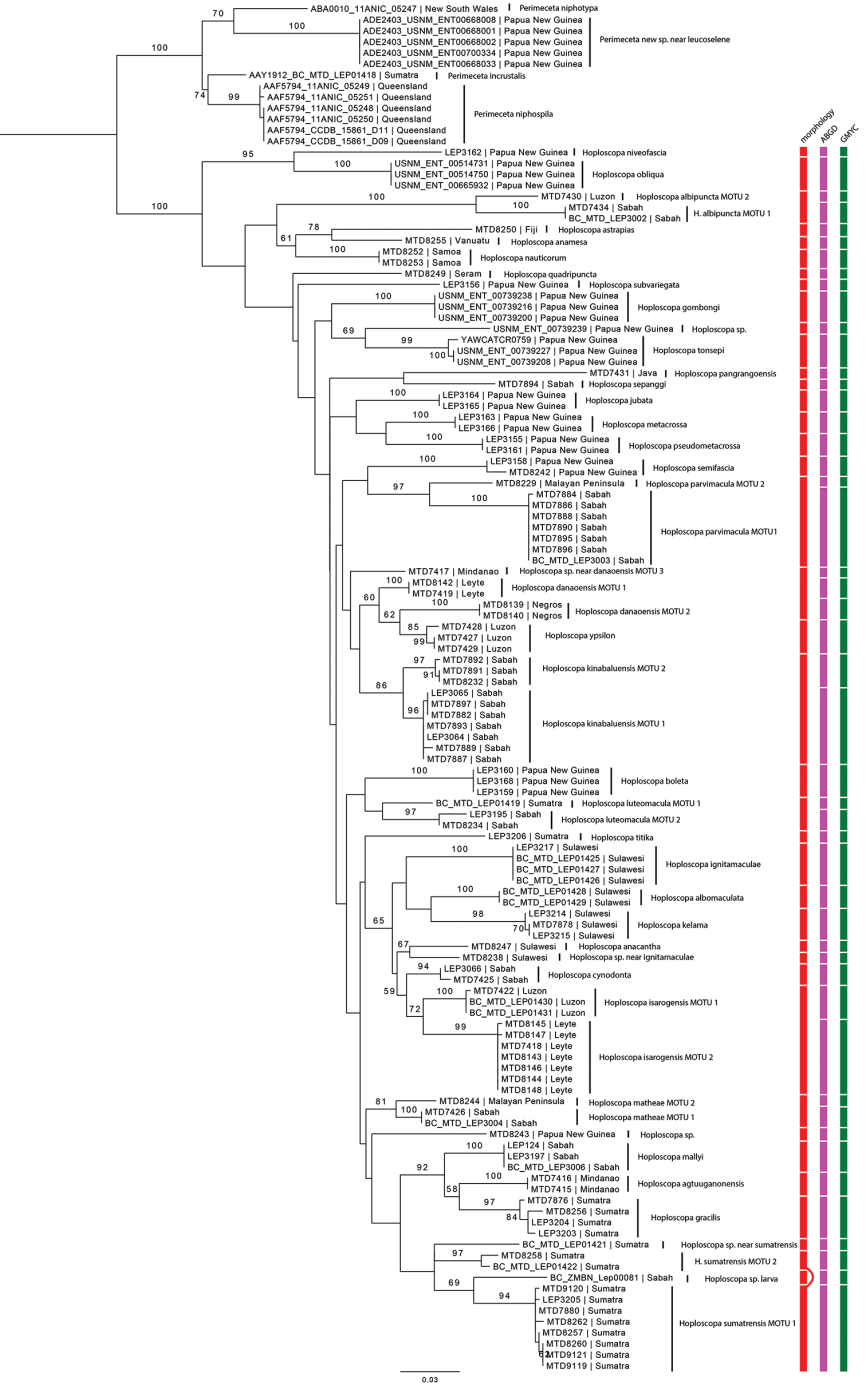
RaxML analysis of the COI barcode dataset partitionned after codon positions. Node support estimated with 1000 thorough bootstrap replicates using the GTR+GAMMA substitution model. Columns on the right display species delimitation inferred from the morphology (red), the ABGD (pink), and GMYC (green) methods.

## Supplementary Material

XML Treatment for Hoploscopa

XML Treatment for Hoploscopa albipuncta

XML Treatment for Hoploscopa matheae

XML Treatment for Hoploscopa sepanggi

XML Treatment for Hoploscopa cynodonta

XML Treatment for Hoploscopa parvimacula

XML Treatment for Hoploscopa kinabaluensis

XML Treatment for Hoploscopa luteomacula

XML Treatment for Hoploscopa obliqua

XML Treatment for Hoploscopa niveofascia

XML Treatment for Hoploscopa gombongi

XML Treatment for Hoploscopa tonsepi

XML Treatment for Hoploscopa marijoweissae

XML Treatment for Hoploscopa titika

XML Treatment for Hoploscopa pangrangoensis

XML Treatment for Hoploscopa isarogensis

XML Treatment for Hoploscopa ypsilon

XML Treatment for Hoploscopa danaoensis

XML Treatment for Hoploscopa aurantiacalis

XML Treatment for Hoploscopa brunnealis

XML Treatment for Hoploscopa ocellata

XML Treatment for Hoploscopa quadripuncta

XML Treatment for Hoploscopa semifascia

XML Treatment for Hoploscopa subvariegata

XML Treatment for Hoploscopa persimilis

XML Treatment for Hoploscopa diffusa

XML Treatment for Hoploscopa triangulifera

XML Treatment for Hoploscopa astrapias

XML Treatment for Hoploscopa anamesa

XML Treatment for Hoploscopa nauticorum

XML Treatment for Hoploscopa anacantha

XML Treatment for Hoploscopa kelama

XML Treatment for Hoploscopa ignitamaculae

XML Treatment for Hoploscopa albomaculata

XML Treatment for Hoploscopa sumatrensis

XML Treatment for Hoploscopa mallyi

XML Treatment for Hoploscopa gracilis

XML Treatment for Hoploscopa agtuuganonensis

XML Treatment for Hoploscopa boleta

XML Treatment for Hoploscopa pseudometacrossa

XML Treatment for Hoploscopa metacrossa

XML Treatment for Hoploscopa jubata

## Appendix 1

**Table A1.** Collecting localities of the *Hoploscopa* specimens examined. GPS coordinates for historical localities were approximated with help of the online gazetteer https://geographic.org/geographic_names/index.html.

Species	Latitude, Longitude	Geographical location	Country	Detailed locality	Remarks
*H. agtuuganonensis*	7.78, 126.21	Mindanao	Philippines	Mount Agtuuganon	
*H. albipuncta*	6.042, 116.596	Borneo	Malaysia	Kopogon	
	6.045, 116.595	Borneo	Malaysia	Mesilau Nature Resort	
	6.028, 116.5483	Borneo	Malaysia	Timpohon Gate, 300m after Ligawu trail start	
	6.0277, 116.5497	Borneo	Malaysia	Timpohon Gate, 700m after Ligawu trail start	
*H. albomaculata*	0.76, 124.46	North Sulawesi	Indonesia	Lake Danau Mooat	
*H. anacantha*	0.56, 123.68	North Sulawesi	Indonesia	Dumoga-Bone NP [Bogani Nani Wartabone NP]	
	0.76, 124.46	North Sulawesi	Indonesia	Lake Danau Mooat	
	0.45, 123.93	North Sulawesi	Indonesia	Mount Mogogonipa	
*H. anamesa*	-20.2, 169.81	Aneityum	Vanuatu	Aneityum	
	-20.23, 169.76	Aneityum	Vanuatu	Anelgauhat	
	-19.5, 169.3	Tanna	Vanuatu	Tanna	
	-17.56, 177.96	Fiji	Fiji	Nandarivatu	
	-17.82, 178.32	Fiji	Fiji	Vunîdawa	
*H. aurantiacalis*	-7.175, 107.57	Pengaleng	Indonesia	Pengaleng	This place could not be assessed with confidence. We refer here to West Java
*H. boleta*	-7.35, 146.68	Papua	Papua New Guinea	Mount Kaindi	
*H. brunnealis*	-7.175, 107.57	Pengaleng	Indonesia	Pengaleng	This place could not be assessed with confidence. We refer here to West Java
*H. cynodonta*	4.555, 115.154	Borneo	Brunei	Ulu Temburong	
	6.009, 116.537	Borneo	Malaysia	Kiau View-Pandanus Trail	
	5.48, 116.56	Borneo	Malaysia	Mount Monkobo	
	4.467, 117.917	Borneo	Malaysia	Tawau Hills HQ	
	6.0277, 116.549	Borneo	Malaysia	Timpohon Gate, 700m after Ligawu trail start	
*H. danaoensis*	11.07, 124.69	Leyte	Philippines	Lake Danao	
*H. diffusa*	-9.5, 150.66	Papua	Papua New Guinea	Fergusson Island	
*H. gombongi*	-6.2, 146.1	Papua	Papua New Guinea	Yawan village	
*H. gracilis*	2.77, 98.53	Sumatra	Indonesia	Dairi	
	3.24, 98.51	Sumatra	Indonesia	Mount Sibayak	
	2.755, 99	Sumatra	Indonesia	Road from Pematangsiantar to Prapat	original label: “Holzweg 2, 25km SSW-Pematangsiantar, Straße nach Prapat”
*H. gracilis*	1.617, 99.267	Sumatra	Indonesia	Sipirok	
*H. ignitamaculae*	0.56, 123.68	North Sulawesi	Indonesia	Dumoga-Bone NP [Bogani Nani Wartabone NP]	
	0.76, 124.46	North Sulawesi	Indonesia	Lake Danau Mooat	
	0.45, 123.93	North Sulawesi	Indonesia	Mount Mogogonipa	
	1.51, 125.18	North Sulawesi	Indonesia	Tangkoko Batuangus Nature Reserve	
*H. isarogensis*	13.66, 123.33	Luzon	Philippines	Mount Isarog	
*H. jubata*	-7.22, 146.61	Papua	Papua New Guinea	Mount Susu NP	
	-7, 146	Papua	Papua New Guinea	Wau Ecology Institute	
*H. kelama*	0.56, 123.68	North Sulawesi	Indonesia	Dumoga-Bone NP [Bogani Nani Wartabone NP]	
	0.76, 124.46	North Sulawesi	Indonesia	Lake Danau Mooat	
*H. kinabaluensis*	6.009, 116.537	Borneo	Malaysia	Kiau View-Pandanus Trail	
	6.0116, 116.5344	Borneo	Malaysia	Kinabalu Mountain Lodge	
	6.028, 116.5483	Borneo	Malaysia	Timpohon Gate, 300m after Ligawu trail start	
	6.0277, 116.5497	Borneo	Malaysia	Timpohon Gate, 700m after Ligawu trail start	
*H. luteomacula*	-0.4, 100.35	Sumatra	Indonesia	Mount Singgalang	
	2.755, 99	Sumatra	Indonesia	Road from Pematangsiantar to Prapat	original label: “Holzweg 2, 25km SSW-Pematangsiantar, Straße nach Prapat”
*H. mallyi*	6.0394, 116.598	Borneo	Malaysia	Mesilau logging site	
	6.045, 116.595	Borneo	Malaysia	Mesilau Nature Resort	
	6.009, 16.5388	Borneo	Malaysia	Pandanus trail, 250m after start	
	6.01, 116.54	Borneo	Malaysia	Sabah, Kundasang, Kinabalu NP Headquarters	
	6.028, 116.5483	Borneo	Malaysia	Timpohon Gate, 300m after Ligawu trail start	
*H. marijoweissae*	-4.4, 139.7	Papua	Indonesia	Mount Goliath [Mount Yamin]	
*H. matheae*	4.33, 115.29	Borneo	Brunei	Bukit Retak	
	6.0116, 116.5344	Borneo	Malaysia	Kinabalu Mountain Lodge	
	4.467, 117.917	Borneo	Malaysia	Tawau Hills HQ	
*H. metacrossa*	-2.91, 132.3	Papua	Indonesia	Fak-Fak	
	-7.22, 146.61	Papua	Papua New Guinea	Mount Susu NP	
	-7, 146	Papua	Papua New Guinea	Wau Ecology Institute	
*H. nauticorum*	-13.9, -171.75	Upolu	Samoa	Malololelei	
	-13.92, 171.75	Upolu	Samoa	Tiavi	
*H. niveofascia*	-7.22, 146.61	Papua	Papua New Guinea	Mount Susu NP	
*H. obliqua*	-4.48, 137.34	Papua	Indonesia	Utakwa [Oetakwa] river	
	-5.25, 145.283	Papua	Papua New Guinea	Wanang village	
	-0.61, 127.3	Moluccas	Indonesia	Batchian [Bacan islands]	
*H. pangrangoensis*	-6.77, 106.96	Java	Indonesia	Mount Pangrango	
*H. parvimacula*	6.0116, 116.5344	Borneo	Malaysia	Kinabalu Mountain Lodge	
	6.0394, 116.598	Borneo	Malaysia	Mesilau logging site	
	6.045, 116.595	Borneo	Malaysia	Mesilau Nature Resort	
	6.028, 116.5483	Borneo	Malaysia	Timpohon Gate, 300m after Ligawu trail start	
*H. persimilis*	-4.48, 137.34	Papua	Indonesia	Utakwa [Oetakwa] river	
*H. pseudometacrossa*	-5.58, 144.07	Papua	Papua New Guinea	Mount Hagen	
*H. quadripuncta*	-3.08, 129.53	Moluccas	Indonesia	Mount Kobipoto	
	-4.48, 137.34	Papua	Indonesia	Utakwa [Oetakwa] river	
	-9, 148.37	Papua	Papua New Guinea	Hydrographers Range	
*H. semifascia*	-2.91, 132.3	Papua	Indonesia	Fak-Fak	
	-6.58, 142.83	Papua	Papua New Guinea	Bosavi (Mount)	
	-7, 146	Papua	Papua New Guinea	Wau Ecology Institute	
*H. sepanggi*	6.0394, 116.598	Borneo	Malaysia	Mesilau logging site	
	6.045, 116.595	Borneo	Malaysia	Mesilau Nature Resort	
	6.028, 116.5483	Borneo	Malaysia	Timpohon Gate, 300m after Ligawu trail start	
*H. subvariegata*	-8.8, 146.5	Papua	Papua New Guinea	Angabunga river	
	-7.35, 146.68	Papua	Papua New Guinea	Mount Kaindi	
*H. sumatrensis*	2.755, 99	Sumatra	Indonesia	Road from Pematangsiantar to Prapat	original label: “Holzweg 2, 25km SSW-Pematangsiantar, Straße nach Prapat”
*H. titika*	2.755, 99	Sumatra	Indonesia	Road from Pematangsiantar to Prapat	original label: “Holzweg 2, 25km SSW-Pematangsiantar, Straße nach Prapat”
*H. tonsepi*	-6.2, 146.1	Papua	Papua New Guinea	Yawan village	
*H. triangulifera*	-4.63, 145.97	Papua	Papua New Guinea	Dampier Island [Karkar]	
	-9.5, 150.66	Papua	Papua New Guinea	Fergusson Island	
	-8.45, 147.81	Papua	Papua New Guinea	Mambare River	
*H. ypsilon*	17.04, 121.1	Luzon	Philippines	Barlig (Chatol)	
	16.97, 121.03	Luzon	Philippines	Mount Polis	
	16.16, 120.94	Luzon	Philippines	Santa Fe	

## Supplementary materials

Supplementary material 1 Figure S1. Scheme summarising the approach followed for amplification of the COI barcode from old material Data type: molecular data https://binary.pensoft.net/file/375777This dataset is made available under the Open Database License (http://opendatacommons.org/licenses/odbl/1.0/). The Open Database License (ODbL) is a license agreement intended to allow users to freely share, modify, and use this Dataset while maintaining this same freedom for others, provided that the original source and author(s) are credited.Théo Léger, Christian Kehlmaier, Charles S. Vairappan, Matthias Nuss

Supplementary material 2 Table S1. Primers sequences with characteristics and chemical properties Data type: molecular data https://binary.pensoft.net/file/375778This dataset is made available under the Open Database License (http://opendatacommons.org/licenses/odbl/1.0/). The Open Database License (ODbL) is a license agreement intended to allow users to freely share, modify, and use this Dataset while maintaining this same freedom for others, provided that the original source and author(s) are credited.Théo Léger, Christian Kehlmaier, Charles S. Vairappan, Matthias Nuss

Supplementary material 3 Table S2. List of DNA samples of *Hoploscopa* moths and allies used in this study Data type: molecular data https://binary.pensoft.net/file/375779This dataset is made available under the Open Database License (http://opendatacommons.org/licenses/odbl/1.0/). The Open Database License (ODbL) is a license agreement intended to allow users to freely share, modify, and use this Dataset while maintaining this same freedom for others, provided that the original source and author(s) are credited.Théo Léger, Christian Kehlmaier, Charles S. Vairappan, Matthias Nuss

Supplementary material 4 Table S3. K2P-distances calculated on the ABGD online server (https://bioinfo.mnhn.fr/abi/public/abgd/abgdweb.html) using the Kimura 2-parameters algorithm Data type: molecular data https://binary.pensoft.net/file/375780This dataset is made available under the Open Database License (http://opendatacommons.org/licenses/odbl/1.0/). The Open Database License (ODbL) is a license agreement intended to allow users to freely share, modify, and use this Dataset while maintaining this same freedom for others, provided that the original source and author(s) are credited.Théo Léger, Christian Kehlmaier, Charles S. Vairappan, Matthias Nuss
