# Supplementary material for: Twenty-six new species of Hoploscopa (Lepidoptera, Crambidae) from South-East Asia revealed by morphology and DNA barcoding
Source: Zookeys. 2020 Jan 29;907:1–99. doi: 10.3897/zookeys.907.36563 (PMC7002455; doi:10.3897/zookeys.907.36563)
Supplement: Supplementary material 1 [file zookeys-907-001-s001.pdf]

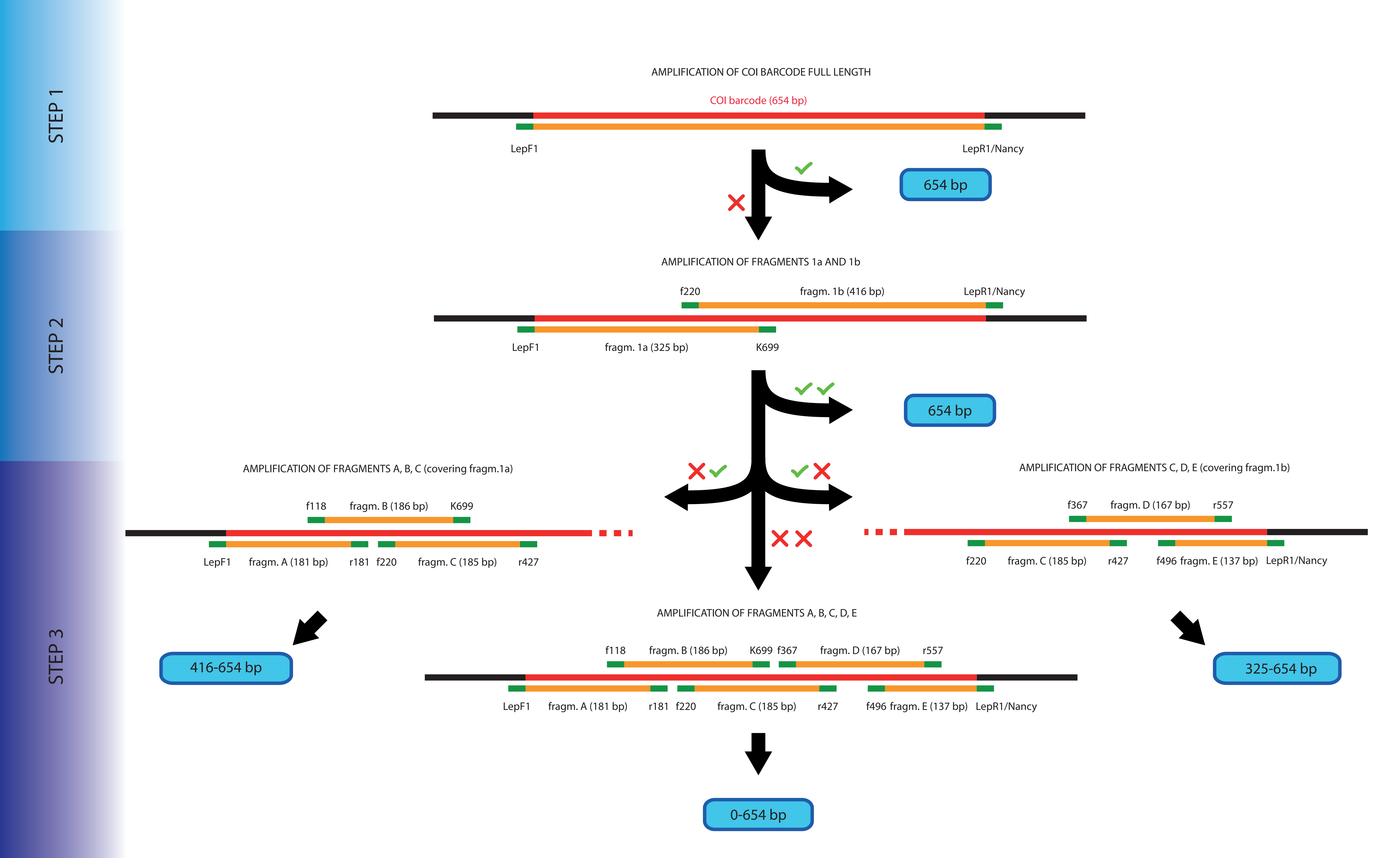
STEP 1
STEP 2
STEP 3
AMPLIFICATION OF COI BARCODE FULL LENGTH
COI barcode (654 bp)
LepF1
LepR1/Nancy
654 bp
AMPLIFICATION OF FRAGMENTS 1a AND 1b
f220
fragm. 1b (416 bp)
LepR1/Nancy
LepF1
fragm. 1a (325 bp)
K699
654 bp
AMPLIFICATION OF FRAGMENTS A, B, C (covering fragm.1a)
f118
fragm. B (186 bp)
K699
LepF1
fragm. A (181 bp)
r181
f220
fragm. C (185 bp)
r427
AMPLIFICATION OF FRAGMENTS C, D, E (covering fragm.1b)
f367
fragm. D (167 bp)
r557
f220
fragm. C (185 bp)
r427
f496 fragm. E (137 bp)
LepR1/Nancy
416-654 bp
325-654 bp
AMPLIFICATION OF FRAGMENTS A, B, C, D, E
f118
fragm. B (186 bp)
K699
f367
fragm. D (167 bp)
r557
LepF1
fragm. A (181 bp)
r181
f220
fragm. C (185 bp)
r427
f496 fragm. E (137 bp)
LepR1/Nancy
0-654 bp
